# Improved luminosity determination in *pp* collisions at $\sqrt {s} = 7\ \mathrm{TeV}$ using the ATLAS detector at the LHC

**DOI:** 10.1140/epjc/s10052-013-2518-3

**Published:** 2013-08-14

**Authors:** The ATLAS Collaboration, G. Aad, T. Abajyan, B. Abbott, J. Abdallah, S. Abdel Khalek, A. A. Abdelalim, O. Abdinov, R. Aben, B. Abi, M. Abolins, O. S. AbouZeid, H. Abramowicz, H. Abreu, E. Acerbi, B. S. Acharya, L. Adamczyk, D. L. Adams, T. N. Addy, J. Adelman, S. Adomeit, P. Adragna, T. Adye, S. Aefsky, J. A. Aguilar-Saavedra, M. Agustoni, M. Aharrouche, S. P. Ahlen, F. Ahles, A. Ahmad, M. Ahsan, G. Aielli, T. Akdogan, T. P. A. Åkesson, G. Akimoto, A. V. Akimov, M. S. Alam, M. A. Alam, J. Albert, S. Albrand, M. Aleksa, I. N. Aleksandrov, F. Alessandria, C. Alexa, G. Alexander, G. Alexandre, T. Alexopoulos, M. Alhroob, M. Aliev, G. Alimonti, J. Alison, B. M. M. Allbrooke, P. P. Allport, S. E. Allwood-Spiers, J. Almond, A. Aloisio, R. Alon, A. Alonso, F. Alonso, B. Alvarez Gonzalez, M. G. Alviggi, K. Amako, C. Amelung, V. V. Ammosov, S. P. Amor Dos Santos, A. Amorim, N. Amram, C. Anastopoulos, L. S. Ancu, N. Andari, T. Andeen, C. F. Anders, G. Anders, K. J. Anderson, A. Andreazza, V. Andrei, M-L. Andrieux, X. S. Anduaga, P. Anger, A. Angerami, F. Anghinolfi, A. Anisenkov, N. Anjos, A. Annovi, A. Antonaki, M. Antonelli, A. Antonov, J. Antos, F. Anulli, M. Aoki, S. Aoun, L. Aperio Bella, R. Apolle, G. Arabidze, I. Aracena, Y. Arai, A. T. H. Arce, S. Arfaoui, J-F. Arguin, E. Arik, M. Arik, A. J. Armbruster, O. Arnaez, V. Arnal, C. Arnault, A. Artamonov, G. Artoni, D. Arutinov, S. Asai, R. Asfandiyarov, S. Ask, B. Åsman, L. Asquith, K. Assamagan, A. Astbury, M. Atkinson, B. Aubert, E. Auge, K. Augsten, M. Aurousseau, G. Avolio, R. Avramidou, D. Axen, G. Azuelos, Y. Azuma, M. A. Baak, G. Baccaglioni, C. Bacci, A. M. Bach, H. Bachacou, K. Bachas, M. Backes, M. Backhaus, E. Badescu, P. Bagnaia, S. Bahinipati, Y. Bai, D. C. Bailey, T. Bain, J. T. Baines, O. K. Baker, M. D. Baker, S. Baker, E. Banas, P. Banerjee, Sw. Banerjee, D. Banfi, A. Bangert, V. Bansal, H. S. Bansil, L. Barak, S. P. Baranov, A. Barbaro Galtieri, T. Barber, E. L. Barberio, D. Barberis, M. Barbero, D. Y. Bardin, T. Barillari, M. Barisonzi, T. Barklow, N. Barlow, B. M. Barnett, R. M. Barnett, A. Baroncelli, G. Barone, A. J. Barr, F. Barreiro, J. Barreiro Guimarães da Costa, P. Barrillon, R. Bartoldus, A. E. Barton, V. Bartsch, A. Basye, R. L. Bates, L. Batkova, J. R. Batley, A. Battaglia, M. Battistin, F. Bauer, H. S. Bawa, S. Beale, T. Beau, P. H. Beauchemin, R. Beccherle, P. Bechtle, H. P. Beck, K. Becker, S. Becker, M. Beckingham, K. H. Becks, A. J. Beddall, A. Beddall, S. Bedikian, V. A. Bednyakov, C. P. Bee, L. J. Beemster, M. Begel, S. Behar Harpaz, P. K. Behera, M. Beimforde, C. Belanger-Champagne, P. J. Bell, W. H. Bell, G. Bella, L. Bellagamba, F. Bellina, M. Bellomo, A. Belloni, O. Beloborodova, K. Belotskiy, O. Beltramello, O. Benary, D. Benchekroun, K. Bendtz, N. Benekos, Y. Benhammou, E. Benhar Noccioli, J. A. Benitez Garcia, D. P. Benjamin, M. Benoit, J. R. Bensinger, K. Benslama, S. Bentvelsen, D. Berge, E. Bergeaas Kuutmann, N. Berger, F. Berghaus, E. Berglund, J. Beringer, P. Bernat, R. Bernhard, C. Bernius, T. Berry, C. Bertella, A. Bertin, F. Bertolucci, M. I. Besana, G. J. Besjes, N. Besson, S. Bethke, W. Bhimji, R. M. Bianchi, M. Bianco, O. Biebel, S. P. Bieniek, K. Bierwagen, J. Biesiada, M. Biglietti, H. Bilokon, M. Bindi, S. Binet, A. Bingul, C. Bini, C. Biscarat, B. Bittner, K. M. Black, R. E. Blair, J.-B. Blanchard, G. Blanchot, T. Blazek, I. Bloch, C. Blocker, J. Blocki, A. Blondel, W. Blum, U. Blumenschein, G. J. Bobbink, V. S. Bobrovnikov, S. S. Bocchetta, A. Bocci, C. R. Boddy, M. Boehler, J. Boek, T. T. Boek, N. Boelaert, J. A. Bogaerts, A. Bogdanchikov, A. Bogouch, C. Bohm, J. Bohm, V. Boisvert, T. Bold, V. Boldea, N. M. Bolnet, M. Bomben, M. Bona, M. Boonekamp, C. N. Booth, S. Bordoni, C. Borer, A. Borisov, G. Borissov, I. Borjanovic, M. Borri, S. Borroni, V. Bortolotto, K. Bos, D. Boscherini, M. Bosman, H. Boterenbrood, J. Bouchami, J. Boudreau, E. V. Bouhova-Thacker, D. Boumediene, C. Bourdarios, N. Bousson, A. Boveia, J. Boyd, I. R. Boyko, I. Bozovic-Jelisavcic, J. Bracinik, P. Branchini, G. W. Brandenburg, A. Brandt, G. Brandt, O. Brandt, U. Bratzler, B. Brau, J. E. Brau, H. M. Braun, S. F. Brazzale, B. Brelier, J. Bremer, K. Brendlinger, R. Brenner, S. Bressler, D. Britton, F. M. Brochu, I. Brock, R. Brock, F. Broggi, C. Bromberg, J. Bronner, G. Brooijmans, T. Brooks, W. K. Brooks, G. Brown, H. Brown, P. A. Bruckman de Renstrom, D. Bruncko, R. Bruneliere, S. Brunet, A. Bruni, G. Bruni, M. Bruschi, T. Buanes, Q. Buat, F. Bucci, J. Buchanan, P. Buchholz, R. M. Buckingham, A. G. Buckley, S. I. Buda, I. A. Budagov, B. Budick, L. Bugge, O. Bulekov, A. C. Bundock, M. Bunse, T. Buran, H. Burckhart, S. Burdin, T. Burgess, S. Burke, E. Busato, V. Büscher, P. Bussey, C. P. Buszello, B. Butler, J. M. Butler, C. M. Buttar, J. M. Butterworth, W. Buttinger, M. Byszewski, S. Cabrera Urbán, D. Caforio, O. Cakir, P. Calafiura, G. Calderini, P. Calfayan, R. Calkins, L. P. Caloba, R. Caloi, D. Calvet, S. Calvet, R. Camacho Toro, P. Camarri, D. Cameron, L. M. Caminada, R. Caminal Armadans, S. Campana, M. Campanelli, V. Canale, F. Canelli, A. Canepa, J. Cantero, R. Cantrill, L. Capasso, M. D. M. Capeans Garrido, I. Caprini, M. Caprini, D. Capriotti, M. Capua, R. Caputo, R. Cardarelli, T. Carli, G. Carlino, L. Carminati, B. Caron, S. Caron, E. Carquin, G. D. Carrillo-Montoya, A. A. Carter, J. R. Carter, J. Carvalho, D. Casadei, M. P. Casado, M. Cascella, C. Caso, A. M. Castaneda Hernandez, E. Castaneda-Miranda, V. Castillo Gimenez, N. F. Castro, G. Cataldi, P. Catastini, A. Catinaccio, J. R. Catmore, A. Cattai, G. Cattani, S. Caughron, V. Cavaliere, P. Cavalleri, D. Cavalli, M. Cavalli-Sforza, V. Cavasinni, F. Ceradini, A. S. Cerqueira, A. Cerri, L. Cerrito, F. Cerutti, S. A. Cetin, A. Chafaq, D. Chakraborty, I. Chalupkova, K. Chan, P. Chang, B. Chapleau, J. D. Chapman, J. W. Chapman, E. Chareyre, D. G. Charlton, V. Chavda, C. A. Chavez Barajas, S. Cheatham, S. Chekanov, S. V. Chekulaev, G. A. Chelkov, M. A. Chelstowska, C. Chen, H. Chen, S. Chen, X. Chen, Y. Chen, A. Cheplakov, R. Cherkaoui El Moursli, V. Chernyatin, E. Cheu, S. L. Cheung, L. Chevalier, G. Chiefari, L. Chikovani, J. T. Childers, A. Chilingarov, G. Chiodini, A. S. Chisholm, R. T. Chislett, A. Chitan, M. V. Chizhov, G. Choudalakis, S. Chouridou, I. A. Christidi, A. Christov, D. Chromek-Burckhart, M. L. Chu, J. Chudoba, G. Ciapetti, A. K. Ciftci, R. Ciftci, D. Cinca, V. Cindro, C. Ciocca, A. Ciocio, M. Cirilli, P. Cirkovic, Z. H. Citron, M. Citterio, M. Ciubancan, A. Clark, P. J. Clark, R. N. Clarke, W. Cleland, J. C. Clemens, B. Clement, C. Clement, Y. Coadou, M. Cobal, A. Coccaro, J. Cochran, J. G. Cogan, J. Coggeshall, E. Cogneras, J. Colas, S. Cole, A. P. Colijn, N. J. Collins, C. Collins-Tooth, J. Collot, T. Colombo, G. Colon, P. Conde Muiño, E. Coniavitis, M. C. Conidi, S. M. Consonni, V. Consorti, S. Constantinescu, C. Conta, G. Conti, F. Conventi, M. Cooke, B. D. Cooper, A. M. Cooper-Sarkar, K. Copic, T. Cornelissen, M. Corradi, F. Corriveau, A. Cortes-Gonzalez, G. Cortiana, G. Costa, M. J. Costa, D. Costanzo, D. Côté, L. Courneyea, G. Cowan, C. Cowden, B. E. Cox, K. Cranmer, S. Crépé-Renaudin, F. Crescioli, M. Cristinziani, G. Crosetti, C.-M. Cuciuc, C. Cuenca Almenar, T. Cuhadar Donszelmann, M. Curatolo, C. J. Curtis, C. Cuthbert, P. Cwetanski, H. Czirr, P. Czodrowski, Z. Czyczula, S. D’Auria, M. D’Onofrio, A. D’Orazio, M. J. Da Cunha Sargedas De Sousa, C. Da Via, W. Dabrowski, A. Dafinca, T. Dai, C. Dallapiccola, M. Dam, M. Dameri, D. S. Damiani, H. O. Danielsson, V. Dao, G. Darbo, G. L. Darlea, J. A. Dassoulas, W. Davey, T. Davidek, N. Davidson, R. Davidson, E. Davies, M. Davies, O. Davignon, A. R. Davison, Y. Davygora, E. Dawe, I. Dawson, R. K. Daya-Ishmukhametova, K. De, R. de Asmundis, S. De Castro, S. De Cecco, J. de Graat, N. De Groot, P. de Jong, C. De La Taille, H. De la Torre, F. De Lorenzi, L. de Mora, L. De Nooij, D. De Pedis, A. De Salvo, U. De Sanctis, A. De Santo, J. B. De Vivie De Regie, G. De Zorzi, W. J. Dearnaley, R. Debbe, C. Debenedetti, B. Dechenaux, D. V. Dedovich, J. Degenhardt, C. Del Papa, J. Del Peso, T. Del Prete, T. Delemontex, M. Deliyergiyev, A. Dell’Acqua, L. Dell’Asta, M. Della Pietra, D. della Volpe, M. Delmastro, P. A. Delsart, C. Deluca, S. Demers, M. Demichev, B. Demirkoz, J. Deng, S. P. Denisov, D. Derendarz, J. E. Derkaoui, F. Derue, P. Dervan, K. Desch, E. Devetak, P. O. Deviveiros, A. Dewhurst, B. DeWilde, S. Dhaliwal, R. Dhullipudi, A. Di Ciaccio, L. Di Ciaccio, A. Di Girolamo, B. Di Girolamo, S. Di Luise, A. Di Mattia, B. Di Micco, R. Di Nardo, A. Di Simone, R. Di Sipio, M. A. Diaz, E. B. Diehl, J. Dietrich, T. A. Dietzsch, S. Diglio, K. Dindar Yagci, J. Dingfelder, F. Dinut, C. Dionisi, P. Dita, S. Dita, F. Dittus, F. Djama, T. Djobava, M. A. B. do Vale, A. Do Valle Wemans, T. K. O. Doan, M. Dobbs, R. Dobinson, D. Dobos, E. Dobson, J. Dodd, C. Doglioni, T. Doherty, T. Dohmae, Y. Doi, J. Dolejsi, I. Dolenc, Z. Dolezal, B. A. Dolgoshein, M. Donadelli, J. Donini, J. Dopke, A. Doria, A. Dos Anjos, A. Dotti, M. T. Dova, A. D. Doxiadis, A. T. Doyle, N. Dressnandt, M. Dris, J. Dubbert, S. Dube, E. Duchovni, G. Duckeck, D. Duda, A. Dudarev, F. Dudziak, I. P. Duerdoth, L. Duflot, M-A. Dufour, L. Duguid, M. Dührssen, M. Dunford, H. Duran Yildiz, M. Düren, R. Duxfield, M. Dwuznik, F. Dydak, W. L. Ebenstein, J. Ebke, S. Eckweiler, K. Edmonds, W. Edson, C. A. Edwards, N. C. Edwards, W. Ehrenfeld, T. Eifert, G. Eigen, K. Einsweiler, E. Eisenhandler, T. Ekelof, M. El Kacimi, M. Ellert, S. Elles, F. Ellinghaus, K. Ellis, N. Ellis, J. Elmsheuser, M. Elsing, D. Emeliyanov, R. Engelmann, A. Engl, B. Epp, J. Erdmann, A. Ereditato, D. Eriksson, J. Ernst, M. Ernst, J. Ernwein, D. Errede, S. Errede, E. Ertel, M. Escalier, H. Esch, C. Escobar, X. Espinal Curull, B. Esposito, F. Etienne, A. I. Etienvre, E. Etzion, D. Evangelakou, H. Evans, L. Fabbri, C. Fabre, R. M. Fakhrutdinov, S. Falciano, Y. Fang, M. Fanti, A. Farbin, A. Farilla, J. Farley, T. Farooque, S. Farrell, S. M. Farrington, P. Farthouat, F. Fassi, P. Fassnacht, D. Fassouliotis, B. Fatholahzadeh, A. Favareto, L. Fayard, S. Fazio, R. Febbraro, P. Federic, O. L. Fedin, W. Fedorko, M. Fehling-Kaschek, L. Feligioni, D. Fellmann, C. Feng, E. J. Feng, A. B. Fenyuk, J. Ferencei, W. Fernando, S. Ferrag, J. Ferrando, V. Ferrara, A. Ferrari, P. Ferrari, R. Ferrari, D. E. Ferreira de Lima, A. Ferrer, D. Ferrere, C. Ferretti, A. Ferretto Parodi, M. Fiascaris, F. Fiedler, A. Filipčič, F. Filthaut, M. Fincke-Keeler, M. C. N. Fiolhais, L. Fiorini, A. Firan, G. Fischer, M. J. Fisher, M. Flechl, I. Fleck, J. Fleckner, P. Fleischmann, S. Fleischmann, T. Flick, A. Floderus, L. R. Flores Castillo, M. J. Flowerdew, T. Fonseca Martin, A. Formica, A. Forti, D. Fortin, D. Fournier, A. J. Fowler, H. Fox, P. Francavilla, M. Franchini, S. Franchino, D. Francis, T. Frank, S. Franz, M. Fraternali, S. Fratina, S. T. French, C. Friedrich, F. Friedrich, R. Froeschl, D. Froidevaux, J. A. Frost, C. Fukunaga, E. Fullana Torregrosa, B. G. Fulsom, J. Fuster, C. Gabaldon, O. Gabizon, T. Gadfort, S. Gadomski, G. Gagliardi, P. Gagnon, C. Galea, B. Galhardo, E. J. Gallas, V. Gallo, B. J. Gallop, P. Gallus, K. K. Gan, Y. S. Gao, A. Gaponenko, F. Garberson, C. García, J. E. García Navarro, M. Garcia-Sciveres, R. W. Gardner, N. Garelli, H. Garitaonandia, V. Garonne, C. Gatti, G. Gaudio, B. Gaur, L. Gauthier, P. Gauzzi, I. L. Gavrilenko, C. Gay, G. Gaycken, E. N. Gazis, P. Ge, Z. Gecse, C. N. P. Gee, D. A. A. Geerts, Ch. Geich-Gimbel, K. Gellerstedt, C. Gemme, A. Gemmell, M. H. Genest, S. Gentile, M. George, S. George, P. Gerlach, A. Gershon, C. Geweniger, H. Ghazlane, N. Ghodbane, B. Giacobbe, S. Giagu, V. Giakoumopoulou, V. Giangiobbe, F. Gianotti, B. Gibbard, A. Gibson, S. M. Gibson, M. Gilchriese, D. Gillberg, A. R. Gillman, D. M. Gingrich, J. Ginzburg, N. Giokaris, M. P. Giordani, R. Giordano, F. M. Giorgi, P. Giovannini, P. F. Giraud, D. Giugni, M. Giunta, P. Giusti, B. K. Gjelsten, L. K. Gladilin, C. Glasman, J. Glatzer, A. Glazov, K. W. Glitza, G. L. Glonti, J. R. Goddard, J. Godfrey, J. Godlewski, M. Goebel, C. Goeringer, S. Goldfarb, T. Golling, A. Gomes, L. S. Gomez Fajardo, R. Gonçalo, J. Goncalves Pinto Firmino Da Costa, L. Gonella, S. Gonzalez, S. González de la Hoz, G. Gonzalez Parra, M. L. Gonzalez Silva, S. Gonzalez-Sevilla, J. J. Goodson, L. Goossens, T. Göpfert, P. A. Gorbounov, H. A. Gordon, I. Gorelov, G. Gorfine, B. Gorini, E. Gorini, A. Gorišek, E. Gornicki, B. Gosdzik, A. T. Goshaw, M. Gosselink, C. Gössling, M. I. Gostkin, I. Gough Eschrich, M. Gouighri, D. Goujdami, M. P. Goulette, A. G. Goussiou, C. Goy, S. Gozpinar, I. Grabowska-Bold, P. Grafström, K-J. Grahn, F. Grancagnolo, S. Grancagnolo, V. Grassi, V. Gratchev, N. Grau, H. M. Gray, J. A. Gray, E. Graziani, O. G. Grebenyuk, T. Greenshaw, Z. D. Greenwood, K. Gregersen, I. M. Gregor, P. Grenier, J. Griffiths, N. Grigalashvili, A. A. Grillo, S. Grinstein, Ph. Gris, Y. V. Grishkevich, J.-F. Grivaz, E. Gross, J. Grosse-Knetter, J. Groth-Jensen, K. Grybel, D. Guest, C. Guicheney, S. Guindon, U. Gul, H. Guler, J. Gunther, B. Guo, J. Guo, P. Gutierrez, N. Guttman, O. Gutzwiller, C. Guyot, C. Gwenlan, C. B. Gwilliam, A. Haas, S. Haas, C. Haber, H. K. Hadavand, D. R. Hadley, P. Haefner, F. Hahn, S. Haider, Z. Hajduk, H. Hakobyan, D. Hall, J. Haller, K. Hamacher, P. Hamal, K. Hamano, M. Hamer, A. Hamilton, S. Hamilton, L. Han, K. Hanagaki, K. Hanawa, M. Hance, C. Handel, P. Hanke, J. R. Hansen, J. B. Hansen, J. D. Hansen, P. H. Hansen, P. Hansson, K. Hara, G. A. Hare, T. Harenberg, S. Harkusha, D. Harper, R. D. Harrington, O. M. Harris, J. Hartert, F. Hartjes, T. Haruyama, A. Harvey, S. Hasegawa, Y. Hasegawa, S. Hassani, S. Haug, M. Hauschild, R. Hauser, M. Havranek, C. M. Hawkes, R. J. Hawkings, A. D. Hawkins, D. Hawkins, T. Hayakawa, T. Hayashi, D. Hayden, C. P. Hays, H. S. Hayward, S. J. Haywood, M. He, S. J. Head, V. Hedberg, L. Heelan, S. Heim, B. Heinemann, S. Heisterkamp, L. Helary, C. Heller, M. Heller, S. Hellman, D. Hellmich, C. Helsens, R. C. W. Henderson, M. Henke, A. Henrichs, A. M. Henriques Correia, S. Henrot-Versille, C. Hensel, T. Henß, C. M. Hernandez, Y. Hernández Jiménez, R. Herrberg, G. Herten, R. Hertenberger, L. Hervas, G. G. Hesketh, N. P. Hessey, E. Higón-Rodriguez, J. C. Hill, K. H. Hiller, S. Hillert, S. J. Hillier, I. Hinchliffe, E. Hines, M. Hirose, F. Hirsch, D. Hirschbuehl, J. Hobbs, N. Hod, M. C. Hodgkinson, P. Hodgson, A. Hoecker, M. R. Hoeferkamp, J. Hoffman, D. Hoffmann, M. Hohlfeld, M. Holder, S. O. Holmgren, T. Holy, J. L. Holzbauer, T. M. Hong, L. Hooft van Huysduynen, S. Horner, J-Y. Hostachy, S. Hou, A. Hoummada, J. Howard, J. Howarth, I. Hristova, J. Hrivnac, T. Hryn’ova, P. J. Hsu, S.-C. Hsu, D. Hu, Z. Hubacek, F. Hubaut, F. Huegging, A. Huettmann, T. B. Huffman, E. W. Hughes, G. Hughes, M. Huhtinen, M. Hurwitz, U. Husemann, N. Huseynov, J. Huston, J. Huth, G. Iacobucci, G. Iakovidis, M. Ibbotson, I. Ibragimov, L. Iconomidou-Fayard, J. Idarraga, P. Iengo, O. Igonkina, Y. Ikegami, M. Ikeno, D. Iliadis, N. Ilic, T. Ince, J. Inigo-Golfin, P. Ioannou, M. Iodice, K. Iordanidou, V. Ippolito, A. Irles Quiles, C. Isaksson, M. Ishino, M. Ishitsuka, R. Ishmukhametov, C. Issever, S. Istin, A. V. Ivashin, W. Iwanski, H. Iwasaki, J. M. Izen, V. Izzo, B. Jackson, J. N. Jackson, P. Jackson, M. R. Jaekel, V. Jain, K. Jakobs, S. Jakobsen, T. Jakoubek, J. Jakubek, D. K. Jana, E. Jansen, H. Jansen, A. Jantsch, M. Janus, R. C. Jared, G. Jarlskog, L. Jeanty, I. Jen-La Plante, D. Jennens, P. Jenni, P. Jež, S. Jézéquel, M. K. Jha, H. Ji, W. Ji, J. Jia, Y. Jiang, M. Jimenez Belenguer, S. Jin, O. Jinnouchi, M. D. Joergensen, D. Joffe, M. Johansen, K. E. Johansson, P. Johansson, S. Johnert, K. A. Johns, K. Jon-And, G. Jones, R. W. L. Jones, T. J. Jones, C. Joram, P. M. Jorge, K. D. Joshi, J. Jovicevic, T. Jovin, X. Ju, C. A. Jung, R. M. Jungst, V. Juranek, P. Jussel, A. Juste Rozas, S. Kabana, M. Kaci, A. Kaczmarska, P. Kadlecik, M. Kado, H. Kagan, M. Kagan, E. Kajomovitz, S. Kalinin, L. V. Kalinovskaya, S. Kama, N. Kanaya, M. Kaneda, S. Kaneti, T. Kanno, V. A. Kantserov, J. Kanzaki, B. Kaplan, A. Kapliy, J. Kaplon, D. Kar, M. Karagounis, K. Karakostas, M. Karnevskiy, V. Kartvelishvili, A. N. Karyukhin, L. Kashif, G. Kasieczka, R. D. Kass, A. Kastanas, Y. Kataoka, E. Katsoufis, J. Katzy, V. Kaushik, K. Kawagoe, T. Kawamoto, G. Kawamura, M. S. Kayl, S. Kazama, V. F. Kazanin, M. Y. Kazarinov, R. Keeler, P. T. Keener, R. Kehoe, M. Keil, G. D. Kekelidze, J. S. Keller, M. Kenyon, O. Kepka, N. Kerschen, B. P. Kerševan, S. Kersten, K. Kessoku, J. Keung, F. Khalil-zada, H. Khandanyan, A. Khanov, D. Kharchenko, A. Khodinov, A. Khomich, T. J. Khoo, G. Khoriauli, A. Khoroshilov, V. Khovanskiy, E. Khramov, J. Khubua, H. Kim, S. H. Kim, N. Kimura, O. Kind, B. T. King, M. King, R. S. B. King, J. Kirk, A. E. Kiryunin, T. Kishimoto, D. Kisielewska, T. Kitamura, T. Kittelmann, K. Kiuchi, E. Kladiva, M. Klein, U. Klein, K. Kleinknecht, M. Klemetti, A. Klier, P. Klimek, A. Klimentov, R. Klingenberg, J. A. Klinger, E. B. Klinkby, T. Klioutchnikova, P. F. Klok, S. Klous, E.-E. Kluge, T. Kluge, P. Kluit, S. Kluth, N. S. Knecht, E. Kneringer, E. B. F. G. Knoops, A. Knue, B. R. Ko, T. Kobayashi, M. Kobel, M. Kocian, P. Kodys, S. Koenig, F. Koetsveld, P. Koevesarki, T. Koffas, E. Koffeman, L. A. Kogan, S. Kohlmann, F. Kohn, Z. Kohout, T. Kohriki, T. Koi, G. M. Kolachev, H. Kolanoski, V. Kolesnikov, I. Koletsou, J. Koll, M. Kollefrath, A. A. Komar, Y. Komori, T. Kondo, K. Köneke, A. C. König, T. Kono, A. I. Kononov, R. Konoplich, N. Konstantinidis, S. Koperny, L. Köpke, K. Korcyl, K. Kordas, A. Korn, A. Korol, I. Korolkov, E. V. Korolkova, V. A. Korotkov, O. Kortner, S. Kortner, V. V. Kostyukhin, S. Kotov, V. M. Kotov, A. Kotwal, C. Kourkoumelis, V. Kouskoura, A. Koutsman, R. Kowalewski, T. Z. Kowalski, W. Kozanecki, A. S. Kozhin, V. Kral, V. A. Kramarenko, G. Kramberger, M. W. Krasny, A. Krasznahorkay, J. K. Kraus, S. Kreiss, F. Krejci, J. Kretzschmar, N. Krieger, P. Krieger, K. Kroeninger, H. Kroha, J. Kroll, J. Kroseberg, J. Krstic, U. Kruchonak, H. Krüger, T. Kruker, N. Krumnack, Z. V. Krumshteyn, T. Kubota, S. Kuday, S. Kuehn, A. Kugel, T. Kuhl, D. Kuhn, V. Kukhtin, Y. Kulchitsky, S. Kuleshov, C. Kummer, M. Kuna, J. Kunkle, A. Kupco, H. Kurashige, M. Kurata, Y. A. Kurochkin, V. Kus, E. S. Kuwertz, M. Kuze, J. Kvita, R. Kwee, A. La Rosa, L. La Rotonda, L. Labarga, J. Labbe, S. Lablak, C. Lacasta, F. Lacava, H. Lacker, D. Lacour, V. R. Lacuesta, E. Ladygin, R. Lafaye, B. Laforge, T. Lagouri, S. Lai, E. Laisne, M. Lamanna, L. Lambourne, C. L. Lampen, W. Lampl, E. Lancon, U. Landgraf, M. P. J. Landon, J. L. Lane, V. S. Lang, C. Lange, A. J. Lankford, F. Lanni, K. Lantzsch, A. Lanza, S. Laplace, C. Lapoire, J. F. Laporte, T. Lari, A. Larner, M. Lassnig, P. Laurelli, V. Lavorini, W. Lavrijsen, P. Laycock, O. Le Dortz, E. Le Guirriec, C. Le Maner, E. Le Menedeu, T. LeCompte, F. Ledroit-Guillon, H. Lee, J. S. H. Lee, S. C. Lee, L. Lee, M. Lefebvre, M. Legendre, F. Legger, C. Leggett, M. Lehmacher, G. Lehmann Miotto, M. A. L. Leite, R. Leitner, D. Lellouch, B. Lemmer, V. Lendermann, K. J. C. Leney, T. Lenz, G. Lenzen, B. Lenzi, K. Leonhardt, S. Leontsinis, F. Lepold, C. Leroy, J-R. Lessard, C. G. Lester, C. M. Lester, J. Levêque, D. Levin, L. J. Levinson, A. Lewis, G. H. Lewis, A. M. Leyko, M. Leyton, B. Li, H. Li, S. Li, X. Li, Z. Liang, H. Liao, B. Liberti, P. Lichard, M. Lichtnecker, K. Lie, W. Liebig, C. Limbach, A. Limosani, M. Limper, S. C. Lin, F. Linde, J. T. Linnemann, E. Lipeles, A. Lipniacka, T. M. Liss, D. Lissauer, A. Lister, A. M. Litke, C. Liu, D. Liu, H. Liu, J. B. Liu, L. Liu, M. Liu, Y. Liu, M. Livan, S. S. A. Livermore, A. Lleres, J. Llorente Merino, S. L. Lloyd, F. Lo Sterzo, E. Lobodzinska, P. Loch, W. S. Lockman, T. Loddenkoetter, F. K. Loebinger, A. E. Loevschall-Jensen, A. Loginov, C. W. Loh, T. Lohse, K. Lohwasser, M. Lokajicek, V. P. Lombardo, R. E. Long, L. Lopes, D. Lopez Mateos, J. Lorenz, N. Lorenzo Martinez, M. Losada, P. Loscutoff, M. J. Losty, X. Lou, A. Lounis, K. F. Loureiro, J. Love, P. A. Love, A. J. Lowe, F. Lu, H. J. Lubatti, C. Luci, A. Lucotte, A. Ludwig, D. Ludwig, I. Ludwig, J. Ludwig, F. Luehring, G. Luijckx, W. Lukas, D. Lumb, L. Luminari, E. Lund, B. Lundberg, J. Lundberg, O. Lundberg, B. Lund-Jensen, J. Lundquist, M. Lungwitz, D. Lynn, E. Lytken, H. Ma, L. L. Ma, G. Maccarrone, A. Macchiolo, B. Maček, J. Machado Miguens, R. Mackeprang, R. J. Madaras, H. J. Maddocks, W. F. Mader, R. Maenner, M. Maeno, T. Maeno, L. Magnoni, E. Magradze, K. Mahboubi, S. Mahmoud, G. Mahout, C. Maiani, C. Maidantchik, A. Maio, S. Majewski, Y. Makida, N. Makovec, P. Mal, B. Malaescu, Pa. Malecki, P. Malecki, V. P. Maleev, F. Malek, U. Mallik, D. Malon, C. Malone, S. Maltezos, V. Malyshev, S. Malyukov, R. Mameghani, J. Mamuzic, A. Manabe, L. Mandelli, I. Mandić, R. Mandrysch, J. Maneira, A. Manfredini, P. S. Mangeard, L. Manhaes de Andrade Filho, J. A. Manjarres Ramos, A. Mann, P. M. Manning, A. Manousakis-Katsikakis, B. Mansoulie, A. Mapelli, L. Mapelli, L. March, J. F. Marchand, F. Marchese, G. Marchiori, M. Marcisovsky, C. P. Marino, F. Marroquim, Z. Marshall, F. K. Martens, L. F. Marti, S. Marti-Garcia, B. Martin, B. Martin, J. P. Martin, T. A. Martin, V. J. Martin, B. Martin dit Latour, M. Martinez, V. Martinez Outschoorn, S. Martin-Haugh, A. C. Martyniuk, M. Marx, F. Marzano, A. Marzin, L. Masetti, T. Mashimo, R. Mashinistov, J. Masik, A. L. Maslennikov, I. Massa, G. Massaro, N. Massol, P. Mastrandrea, A. Mastroberardino, T. Masubuchi, P. Matricon, H. Matsunaga, T. Matsushita, P. Mättig, S. Mättig, C. Mattravers, J. Maurer, S. J. Maxfield, A. Mayne, R. Mazini, M. Mazur, L. Mazzaferro, M. Mazzanti, J. Mc Donald, S. P. Mc Kee, A. McCarn, R. L. McCarthy, T. G. McCarthy, N. A. McCubbin, K. W. McFarlane, J. A. Mcfayden, G. Mchedlidze, T. Mclaughlan, S. J. McMahon, R. A. McPherson, A. Meade, J. Mechnich, M. Mechtel, M. Medinnis, R. Meera-Lebbai, T. Meguro, R. Mehdiyev, S. Mehlhase, A. Mehta, K. Meier, B. Meirose, C. Melachrinos, B. R. Mellado Garcia, F. Meloni, L. Mendoza Navas, Z. Meng, A. Mengarelli, S. Menke, E. Meoni, K. M. Mercurio, P. Mermod, L. Merola, C. Meroni, F. S. Merritt, H. Merritt, A. Messina, J. Metcalfe, A. S. Mete, C. Meyer, C. Meyer, J-P. Meyer, J. Meyer, J. Meyer, T. C. Meyer, J. Miao, S. Michal, L. Micu, R. P. Middleton, S. Migas, L. Mijović, G. Mikenberg, M. Mikestikova, M. Mikuž, D. W. Miller, R. J. Miller, W. J. Mills, C. Mills, A. Milov, D. A. Milstead, D. Milstein, A. A. Minaenko, M. Miñano Moya, I. A. Minashvili, A. I. Mincer, B. Mindur, M. Mineev, Y. Ming, L. M. Mir, G. Mirabelli, J. Mitrevski, V. A. Mitsou, S. Mitsui, P. S. Miyagawa, J. U. Mjörnmark, T. Moa, V. Moeller, S. Mohapatra, W. Mohr, R. Moles-Valls, A. Molfetas, K. Mönig, J. Monk, E. Monnier, J. Montejo Berlingen, F. Monticelli, S. Monzani, R. W. Moore, G. F. Moorhead, C. Mora Herrera, A. Moraes, N. Morange, J. Morel, G. Morello, D. Moreno, M. Moreno Llácer, P. Morettini, M. Morgenstern, M. Morii, A. K. Morley, G. Mornacchi, J. D. Morris, L. Morvaj, N. Möser, H. G. Moser, M. Mosidze, J. Moss, R. Mount, E. Mountricha, S. V. Mouraviev, E. J. W. Moyse, F. Mueller, J. Mueller, K. Mueller, T. Mueller, D. Muenstermann, T. A. Müller, Y. Munwes, W. J. Murray, I. Mussche, E. Musto, A. G. Myagkov, M. Myska, J. Nadal, K. Nagai, R. Nagai, K. Nagano, A. Nagarkar, Y. Nagasaka, M. Nagel, A. M. Nairz, Y. Nakahama, K. Nakamura, T. Nakamura, I. Nakano, G. Nanava, A. Napier, R. Narayan, M. Nash, T. Nattermann, T. Naumann, G. Navarro, H. A. Neal, P. Yu. Nechaeva, T. J. Neep, A. Negri, G. Negri, M. Negrini, S. Nektarijevic, A. Nelson, T. K. Nelson, S. Nemecek, P. Nemethy, A. A. Nepomuceno, M. Nessi, M. S. Neubauer, M. Neumann, A. Neusiedl, R. M. Neves, P. Nevski, F. M. Newcomer, P. R. Newman, V. Nguyen Thi Hong, R. B. Nickerson, R. Nicolaidou, B. Nicquevert, F. Niedercorn, J. Nielsen, N. Nikiforou, A. Nikiforov, V. Nikolaenko, I. Nikolic-Audit, K. Nikolics, K. Nikolopoulos, H. Nilsen, P. Nilsson, Y. Ninomiya, A. Nisati, R. Nisius, T. Nobe, L. Nodulman, M. Nomachi, I. Nomidis, S. Norberg, M. Nordberg, P. R. Norton, J. Novakova, M. Nozaki, L. Nozka, I. M. Nugent, A.-E. Nuncio-Quiroz, G. Nunes Hanninger, T. Nunnemann, E. Nurse, B. J. O’Brien, S. W. O’Neale, D. C. O’Neil, V. O’Shea, L. B. Oakes, F. G. Oakham, H. Oberlack, J. Ocariz, A. Ochi, S. Oda, S. Odaka, J. Odier, H. Ogren, A. Oh, S. H. Oh, C. C. Ohm, T. Ohshima, H. Okawa, Y. Okumura, T. Okuyama, A. Olariu, A. G. Olchevski, S. A. Olivares Pino, M. Oliveira, D. Oliveira Damazio, E. Oliver Garcia, D. Olivito, A. Olszewski, J. Olszowska, A. Onofre, P. U. E. Onyisi, C. J. Oram, M. J. Oreglia, Y. Oren, D. Orestano, N. Orlando, I. Orlov, C. Oropeza Barrera, R. S. Orr, B. Osculati, R. Ospanov, C. Osuna, G. Otero y Garzon, J. P. Ottersbach, M. Ouchrif, E. A. Ouellette, F. Ould-Saada, A. Ouraou, Q. Ouyang, A. Ovcharova, M. Owen, S. Owen, V. E. Ozcan, N. Ozturk, A. Pacheco Pages, C. Padilla Aranda, S. Pagan Griso, E. Paganis, C. Pahl, F. Paige, P. Pais, K. Pajchel, G. Palacino, C. P. Paleari, S. Palestini, D. Pallin, A. Palma, J. D. Palmer, Y. B. Pan, E. Panagiotopoulou, P. Pani, N. Panikashvili, S. Panitkin, D. Pantea, A. Papadelis, Th. D. Papadopoulou, A. Paramonov, D. Paredes Hernandez, W. Park, M. A. Parker, F. Parodi, J. A. Parsons, U. Parzefall, S. Pashapour, E. Pasqualucci, S. Passaggio, A. Passeri, F. Pastore, Fr. Pastore, G. Pásztor, S. Pataraia, N. D. Patel, J. R. Pater, S. Patricelli, T. Pauly, M. Pecsy, S. Pedraza Lopez, M. I. Pedraza Morales, S. V. Peleganchuk, D. Pelikan, H. Peng, B. Penning, A. Penson, J. Penwell, M. Perantoni, K. Perez, T. Perez Cavalcanti, E. Perez Codina, M. T. Pérez García-Estañ, V. Perez Reale, L. Perini, H. Pernegger, R. Perrino, P. Perrodo, V. D. Peshekhonov, K. Peters, B. A. Petersen, J. Petersen, T. C. Petersen, E. Petit, A. Petridis, C. Petridou, E. Petrolo, F. Petrucci, D. Petschull, M. Petteni, R. Pezoa, A. Phan, P. W. Phillips, G. Piacquadio, A. Picazio, E. Piccaro, M. Piccinini, S. M. Piec, R. Piegaia, D. T. Pignotti, J. E. Pilcher, A. D. Pilkington, J. Pina, M. Pinamonti, A. Pinder, J. L. Pinfold, B. Pinto, C. Pizio, M. Plamondon, M.-A. Pleier, E. Plotnikova, A. Poblaguev, S. Poddar, F. Podlyski, L. Poggioli, D. Pohl, M. Pohl, G. Polesello, A. Policicchio, A. Polini, J. Poll, V. Polychronakos, D. Pomeroy, K. Pommès, L. Pontecorvo, B. G. Pope, G. A. Popeneciu, D. S. Popovic, A. Poppleton, X. Portell Bueso, G. E. Pospelov, S. Pospisil, I. N. Potrap, C. J. Potter, C. T. Potter, G. Poulard, J. Poveda, V. Pozdnyakov, R. Prabhu, P. Pralavorio, A. Pranko, S. Prasad, R. Pravahan, S. Prell, K. Pretzl, D. Price, J. Price, L. E. Price, D. Prieur, M. Primavera, K. Prokofiev, F. Prokoshin, S. Protopopescu, J. Proudfoot, X. Prudent, M. Przybycien, H. Przysiezniak, S. Psoroulas, E. Ptacek, E. Pueschel, J. Purdham, M. Purohit, P. Puzo, Y. Pylypchenko, J. Qian, A. Quadt, D. R. Quarrie, W. B. Quayle, F. Quinonez, M. Raas, V. Radeka, V. Radescu, P. Radloff, T. Rador, F. Ragusa, G. Rahal, A. M. Rahimi, D. Rahm, S. Rajagopalan, M. Rammensee, M. Rammes, A. S. Randle-Conde, K. Randrianarivony, F. Rauscher, T. C. Rave, M. Raymond, A. L. Read, D. M. Rebuzzi, A. Redelbach, G. Redlinger, R. Reece, K. Reeves, E. Reinherz-Aronis, A. Reinsch, I. Reisinger, C. Rembser, Z. L. Ren, A. Renaud, M. Rescigno, S. Resconi, B. Resende, P. Reznicek, R. Rezvani, R. Richter, E. Richter-Was, M. Ridel, M. Rijpstra, M. Rijssenbeek, A. Rimoldi, L. Rinaldi, R. R. Rios, I. Riu, G. Rivoltella, F. Rizatdinova, E. Rizvi, S. H. Robertson, A. Robichaud-Veronneau, D. Robinson, J. E. M. Robinson, A. Robson, J. G. Rocha de Lima, C. Roda, D. Roda Dos Santos, A. Roe, S. Roe, O. Røhne, S. Rolli, A. Romaniouk, M. Romano, G. Romeo, E. Romero Adam, N. Rompotis, L. Roos, E. Ros, S. Rosati, K. Rosbach, A. Rose, M. Rose, G. A. Rosenbaum, E. I. Rosenberg, P. L. Rosendahl, O. Rosenthal, L. Rosselet, V. Rossetti, E. Rossi, L. P. Rossi, M. Rotaru, I. Roth, J. Rothberg, D. Rousseau, C. R. Royon, A. Rozanov, Y. Rozen, X. Ruan, F. Rubbo, I. Rubinskiy, N. Ruckstuhl, V. I. Rud, C. Rudolph, G. Rudolph, F. Rühr, A. Ruiz-Martinez, L. Rumyantsev, Z. Rurikova, N. A. Rusakovich, J. P. Rutherfoord, C. Ruwiedel, P. Ruzicka, Y. F. Ryabov, M. Rybar, G. Rybkin, N. C. Ryder, A. F. Saavedra, I. Sadeh, H. F-W. Sadrozinski, R. Sadykov, F. Safai Tehrani, H. Sakamoto, G. Salamanna, A. Salamon, M. Saleem, D. Salek, D. Salihagic, A. Salnikov, J. Salt, B. M. Salvachua Ferrando, D. Salvatore, F. Salvatore, A. Salvucci, A. Salzburger, D. Sampsonidis, B. H. Samset, A. Sanchez, J. Sánchez, V. Sanchez Martinez, H. Sandaker, H. G. Sander, M. P. Sanders, M. Sandhoff, T. Sandoval, C. Sandoval, R. Sandstroem, D. P. C. Sankey, A. Sansoni, C. Santamarina Rios, C. Santoni, R. Santonico, H. Santos, J. G. Saraiva, T. Sarangi, E. Sarkisyan-Grinbaum, F. Sarri, G. Sartisohn, O. Sasaki, Y. Sasaki, N. Sasao, I. Satsounkevitch, G. Sauvage, E. Sauvan, J. B. Sauvan, P. Savard, V. Savinov, D. O. Savu, L. Sawyer, D. H. Saxon, J. Saxon, C. Sbarra, A. Sbrizzi, D. A. Scannicchio, M. Scarcella, J. Schaarschmidt, P. Schacht, D. Schaefer, S. Schaepe, S. Schaetzel, U. Schäfer, A. C. Schaffer, D. Schaile, R. D. Schamberger, A. G. Schamov, V. Scharf, V. A. Schegelsky, D. Scheirich, M. Schernau, M. I. Scherzer, C. Schiavi, J. Schieck, M. Schioppa, S. Schlenker, E. Schmidt, K. Schmieden, C. Schmitt, S. Schmitt, M. Schmitz, B. Schneider, U. Schnoor, A. Schoening, A. L. S. Schorlemmer, M. Schott, D. Schouten, J. Schovancova, M. Schram, C. Schroeder, N. Schroer, M. J. Schultens, J. Schultes, H.-C. Schultz-Coulon, H. Schulz, M. Schumacher, B. A. Schumm, Ph. Schune, C. Schwanenberger, A. Schwartzman, Ph. Schwegler, Ph. Schwemling, R. Schwienhorst, R. Schwierz, J. Schwindling, T. Schwindt, M. Schwoerer, G. Sciolla, W. G. Scott, J. Searcy, G. Sedov, E. Sedykh, S. C. Seidel, A. Seiden, F. Seifert, J. M. Seixas, G. Sekhniaidze, S. J. Sekula, K. E. Selbach, D. M. Seliverstov, B. Sellden, G. Sellers, M. Seman, N. Semprini-Cesari, C. Serfon, L. Serin, L. Serkin, R. Seuster, H. Severini, A. Sfyrla, E. Shabalina, M. Shamim, L. Y. Shan, J. T. Shank, Q. T. Shao, M. Shapiro, P. B. Shatalov, K. Shaw, D. Sherman, P. Sherwood, A. Shibata, S. Shimizu, M. Shimojima, T. Shin, M. Shiyakova, A. Shmeleva, M. J. Shochet, D. Short, S. Shrestha, E. Shulga, M. A. Shupe, P. Sicho, A. Sidoti, F. Siegert, Dj. Sijacki, O. Silbert, J. Silva, Y. Silver, D. Silverstein, S. B. Silverstein, V. Simak, O. Simard, Lj. Simic, S. Simion, E. Simioni, B. Simmons, R. Simoniello, M. Simonyan, P. Sinervo, N. B. Sinev, V. Sipica, G. Siragusa, A. Sircar, A. N. Sisakyan, S. Yu. Sivoklokov, J. Sjölin, T. B. Sjursen, L. A. Skinnari, H. P. Skottowe, K. Skovpen, P. Skubic, M. Slater, T. Slavicek, K. Sliwa, V. Smakhtin, B. H. Smart, L. Smestad, S. Yu. Smirnov, Y. Smirnov, L. N. Smirnova, O. Smirnova, B. C. Smith, D. Smith, K. M. Smith, M. Smizanska, K. Smolek, A. A. Snesarev, S. W. Snow, J. Snow, S. Snyder, R. Sobie, J. Sodomka, A. Soffer, D. A. Soh, C. A. Solans, M. Solar, J. Solc, E. Yu. Soldatov, U. Soldevila, E. Solfaroli Camillocci, A. A. Solodkov, O. V. Solovyanov, V. Solovyev, N. Soni, V. Sopko, B. Sopko, M. Sosebee, R. Soualah, A. Soukharev, S. Spagnolo, F. Spanò, R. Spighi, G. Spigo, R. Spiwoks, M. Spousta, T. Spreitzer, B. Spurlock, R. D. St. Denis, J. Stahlman, R. Stamen, E. Stanecka, R. W. Stanek, C. Stanescu, M. Stanescu-Bellu, M. M. Stanitzki, S. Stapnes, E. A. Starchenko, J. Stark, P. Staroba, P. Starovoitov, R. Staszewski, A. Staude, P. Stavina, G. Steele, P. Steinbach, P. Steinberg, I. Stekl, B. Stelzer, H. J. Stelzer, O. Stelzer-Chilton, H. Stenzel, S. Stern, G. A. Stewart, J. A. Stillings, M. C. Stockton, K. Stoerig, G. Stoicea, S. Stonjek, P. Strachota, A. R. Stradling, A. Straessner, J. Strandberg, S. Strandberg, A. Strandlie, M. Strang, E. Strauss, M. Strauss, P. Strizenec, R. Ströhmer, D. M. Strom, J. A. Strong, R. Stroynowski, J. Strube, B. Stugu, I. Stumer, J. Stupak, P. Sturm, N. A. Styles, D. Su, HS. Subramania, A. Succurro, Y. Sugaya, C. Suhr, M. Suk, V. V. Sulin, S. Sultansoy, T. Sumida, X. Sun, J. E. Sundermann, K. Suruliz, G. Susinno, M. R. Sutton, Y. Suzuki, Y. Suzuki, M. Svatos, S. Swedish, I. Sykora, T. Sykora, D. Ta, K. Tackmann, A. Taffard, R. Tafirout, N. Taiblum, Y. Takahashi, H. Takai, R. Takashima, H. Takeda, T. Takeshita, Y. Takubo, M. Talby, A. Talyshev, M. C. Tamsett, K. G. Tan, J. Tanaka, R. Tanaka, S. Tanaka, S. Tanaka, A. J. Tanasijczuk, K. Tani, N. Tannoury, S. Tapprogge, D. Tardif, S. Tarem, F. Tarrade, G. F. Tartarelli, P. Tas, M. Tasevsky, E. Tassi, M. Tatarkhanov, Y. Tayalati, C. Taylor, F. E. Taylor, G. N. Taylor, W. Taylor, M. Teinturier, F. A. Teischinger, M. Teixeira Dias Castanheira, P. Teixeira-Dias, K. K. Temming, H. Ten Kate, P. K. Teng, S. Terada, K. Terashi, J. Terron, M. Testa, R. J. Teuscher, J. Therhaag, T. Theveneaux-Pelzer, S. Thoma, J. P. Thomas, E. N. Thompson, P. D. Thompson, P. D. Thompson, A. S. Thompson, L. A. Thomsen, E. Thomson, M. Thomson, W. M. Thong, R. P. Thun, F. Tian, M. J. Tibbetts, T. Tic, V. O. Tikhomirov, Y. A. Tikhonov, S. Timoshenko, P. Tipton, S. Tisserant, T. Todorov, S. Todorova-Nova, B. Toggerson, J. Tojo, S. Tokár, K. Tokushuku, K. Tollefson, L. Tomlinson, M. Tomoto, L. Tompkins, K. Toms, A. Tonoyan, C. Topfel, N. D. Topilin, I. Torchiani, E. Torrence, H. Torres, E. Torró Pastor, J. Toth, F. Touchard, D. R. Tovey, T. Trefzger, L. Tremblet, A. Tricoli, I. M. Trigger, S. Trincaz-Duvoid, M. F. Tripiana, N. Triplett, W. Trischuk, B. Trocmé, C. Troncon, M. Trottier-McDonald, M. Trzebinski, A. Trzupek, C. Tsarouchas, J. C-L. Tseng, M. Tsiakiris, P. V. Tsiareshka, D. Tsionou, G. Tsipolitis, S. Tsiskaridze, V. Tsiskaridze, E. G. Tskhadadze, I. I. Tsukerman, V. Tsulaia, J.-W. Tsung, S. Tsuno, D. Tsybychev, A. Tua, A. Tudorache, V. Tudorache, J. M. Tuggle, M. Turala, D. Turecek, I. Turk Cakir, E. Turlay, R. Turra, P. M. Tuts, A. Tykhonov, M. Tylmad, M. Tyndel, G. Tzanakos, K. Uchida, I. Ueda, R. Ueno, M. Ugland, M. Uhlenbrock, M. Uhrmacher, F. Ukegawa, G. Unal, A. Undrus, G. Unel, Y. Unno, D. Urbaniec, G. Usai, M. Uslenghi, L. Vacavant, V. Vacek, B. Vachon, S. Vahsen, J. Valenta, S. Valentinetti, A. Valero, S. Valkar, E. Valladolid Gallego, S. Vallecorsa, J. A. Valls Ferrer, R. Van Berg, P. C. Van Der Deijl, R. van der Geer, H. van der Graaf, R. Van Der Leeuw, E. van der Poel, D. van der Ster, N. van Eldik, P. van Gemmeren, I. van Vulpen, M. Vanadia, W. Vandelli, A. Vaniachine, P. Vankov, F. Vannucci, R. Vari, E. W. Varnes, T. Varol, D. Varouchas, A. Vartapetian, K. E. Varvell, V. I. Vassilakopoulos, F. Vazeille, T. Vazquez Schroeder, G. Vegni, J. J. Veillet, F. Veloso, R. Veness, S. Veneziano, A. Ventura, D. Ventura, M. Venturi, N. Venturi, V. Vercesi, M. Verducci, W. Verkerke, J. C. Vermeulen, A. Vest, M. C. Vetterli, I. Vichou, T. Vickey, O. E. Vickey Boeriu, G. H. A. Viehhauser, S. Viel, M. Villa, M. Villaplana Perez, E. Vilucchi, M. G. Vincter, E. Vinek, V. B. Vinogradov, M. Virchaux, J. Virzi, O. Vitells, M. Viti, I. Vivarelli, F. Vives Vaque, S. Vlachos, D. Vladoiu, M. Vlasak, A. Vogel, P. Vokac, G. Volpi, M. Volpi, G. Volpini, H. von der Schmitt, H. von Radziewski, E. von Toerne, V. Vorobel, V. Vorwerk, M. Vos, R. Voss, J. H. Vossebeld, N. Vranjes, M. Vranjes Milosavljevic, V. Vrba, M. Vreeswijk, T. Vu Anh, R. Vuillermet, I. Vukotic, W. Wagner, P. Wagner, H. Wahlen, S. Wahrmund, J. Wakabayashi, S. Walch, J. Walder, R. Walker, W. Walkowiak, R. Wall, P. Waller, B. Walsh, C. Wang, H. Wang, H. Wang, J. Wang, J. Wang, R. Wang, S. M. Wang, T. Wang, A. Warburton, C. P. Ward, M. Warsinsky, A. Washbrook, C. Wasicki, I. Watanabe, P. M. Watkins, A. T. Watson, I. J. Watson, M. F. Watson, G. Watts, S. Watts, A. T. Waugh, B. M. Waugh, M. S. Weber, P. Weber, A. R. Weidberg, P. Weigell, J. Weingarten, C. Weiser, P. S. Wells, T. Wenaus, D. Wendland, Z. Weng, T. Wengler, S. Wenig, N. Wermes, M. Werner, P. Werner, M. Werth, M. Wessels, J. Wetter, C. Weydert, K. Whalen, S. J. Wheeler-Ellis, A. White, M. J. White, S. White, S. R. Whitehead, D. Whiteson, D. Whittington, F. Wicek, D. Wicke, F. J. Wickens, W. Wiedenmann, M. Wielers, P. Wienemann, C. Wiglesworth, L. A. M. Wiik-Fuchs, P. A. Wijeratne, A. Wildauer, M. A. Wildt, I. Wilhelm, H. G. Wilkens, J. Z. Will, E. Williams, H. H. Williams, W. Willis, S. Willocq, J. A. Wilson, M. G. Wilson, A. Wilson, I. Wingerter-Seez, S. Winkelmann, F. Winklmeier, M. Wittgen, S. J. Wollstadt, M. W. Wolter, H. Wolters, W. C. Wong, G. Wooden, B. K. Wosiek, J. Wotschack, M. J. Woudstra, K. W. Wozniak, K. Wraight, M. Wright, B. Wrona, S. L. Wu, X. Wu, Y. Wu, E. Wulf, B. M. Wynne, S. Xella, M. Xiao, S. Xie, C. Xu, D. Xu, B. Yabsley, S. Yacoob, M. Yamada, H. Yamaguchi, A. Yamamoto, K. Yamamoto, S. Yamamoto, T. Yamamura, T. Yamanaka, J. Yamaoka, T. Yamazaki, Y. Yamazaki, Z. Yan, H. Yang, U. K. Yang, Y. Yang, Z. Yang, S. Yanush, L. Yao, Y. Yao, Y. Yasu, G. V. Ybeles Smit, J. Ye, S. Ye, M. Yilmaz, R. Yoosoofmiya, K. Yorita, R. Yoshida, C. Young, C. J. Young, S. Youssef, D. Yu, D. R. Yu, J. Yu, J. Yu, L. Yuan, A. Yurkewicz, B. Zabinski, R. Zaidan, A. M. Zaitsev, Z. Zajacova, L. Zanello, D. Zanzi, A. Zaytsev, C. Zeitnitz, M. Zeman, A. Zemla, C. Zendler, O. Zenin, T. Ženiš, S. Zenz, D. Zerwas, G. Zevi della Porta, Z. Zhan, D. Zhang, H. Zhang, J. Zhang, X. Zhang, Z. Zhang, L. Zhao, T. Zhao, Z. Zhao, A. Zhemchugov, J. Zhong, B. Zhou, N. Zhou, Y. Zhou, C. G. Zhu, H. Zhu, J. Zhu, Y. Zhu, X. Zhuang, V. Zhuravlov, D. Zieminska, N. I. Zimin, R. Zimmermann, S. Zimmermann, S. Zimmermann, Z. Zinonos, M. Ziolkowski, R. Zitoun, L. Živković, V. V. Zmouchko, G. Zobernig, A. Zoccoli, M. zur Nedden, V. Zutshi, L. Zwalinski

**Affiliations:** 1 CERN, 1211 Geneva 23, Switzerland; 2 School of Chemistry and Physics, University of Adelaide, Adelaide, Australia; 3 Physics Department, SUNY Albany, Albany, NY United States of America; 4 Department of Physics, University of Alberta, Edmonton, AB Canada; 5 Department of Physics, Ankara University, Ankara, Turkey; 6 Department of Physics, Dumlupinar University, Kutahya, Turkey; 7 Department of Physics, Gazi University, Ankara, Turkey; 8 Division of Physics, TOBB University of Economics and Technology, Ankara, Turkey; 9 Turkish Atomic Energy Authority, Ankara, Turkey; 10 LAPP, CNRS/IN2P3 and Université de Savoie, Annecy-le-Vieux, France; 11 High Energy Physics Division, Argonne National Laboratory, Argonne, IL United States of America; 12 Department of Physics, University of Arizona, Tucson, AZ United States of America; 13 Department of Physics, The University of Texas at Arlington, Arlington, TX United States of America; 14 Physics Department, University of Athens, Athens, Greece; 15 Physics Department, National Technical University of Athens, Zografou, Greece; 16 Institute of Physics, Azerbaijan Academy of Sciences, Baku, Azerbaijan; 17 Institut de Física d’Altes Energies and Departament de Física de la Universitat Autònoma de Barcelona and ICREA, Barcelona, Spain; 18 Institute of Physics, University of Belgrade, Belgrade, Serbia; 19 Vinca Institute of Nuclear Sciences, University of Belgrade, Belgrade, Serbia; 20 Department for Physics and Technology, University of Bergen, Bergen, Norway; 21 Physics Division, Lawrence Berkeley National Laboratory and University of California, Berkeley, CA United States of America; 22 Department of Physics, Humboldt University, Berlin, Germany; 23 Albert Einstein Center for Fundamental Physics and Laboratory for High Energy Physics, University of Bern, Bern, Switzerland; 24 School of Physics and Astronomy, University of Birmingham, Birmingham, United Kingdom; 25 Department of Physics, Bogazici University, Istanbul, Turkey; 26 Division of Physics, Dogus University, Istanbul, Turkey; 27 Department of Physics Engineering, Gaziantep University, Gaziantep, Turkey; 28 Department of Physics, Istanbul Technical University, Istanbul, Turkey; 29 INFN Sezione di Bologna, Bologna, Italy; 30 Dipartimento di Fisica, Università di Bologna, Bologna, Italy; 31 Physikalisches Institut, University of Bonn, Bonn, Germany; 32 Department of Physics, Boston University, Boston, MA United States of America; 33 Department of Physics, Brandeis University, Waltham, MA United States of America; 34 Universidade Federal do Rio De Janeiro COPPE/EE/IF, Rio de Janeiro, Brazil; 35 Federal University of Juiz de Fora (UFJF), Juiz de Fora, Brazil; 36 Federal University of Sao Joao del Rei (UFSJ), Sao Joao del Rei, Brazil; 37 Instituto de Fisica, Universidade de Sao Paulo, Sao Paulo, Brazil; 38 Physics Department, Brookhaven National Laboratory, Upton, NY United States of America; 39 National Institute of Physics and Nuclear Engineering, Bucharest, Romania; 40 University Politehnica Bucharest, Bucharest, Romania; 41 West University in Timisoara, Timisoara, Romania; 42 Departamento de Física, Universidad de Buenos Aires, Buenos Aires, Argentina; 43 Cavendish Laboratory, University of Cambridge, Cambridge, United Kingdom; 44 Department of Physics, Carleton University, Ottawa, ON Canada; 45 CERN, Geneva, Switzerland; 46 Enrico Fermi Institute, University of Chicago, Chicago, IL United States of America; 47 Departamento de Física, Pontificia Universidad Católica de Chile, Santiago, Chile; 48 Departamento de Física, Universidad Técnica Federico Santa María, Valparaíso, Chile; 49 Institute of High Energy Physics, Chinese Academy of Sciences, Beijing, China; 50 Department of Modern Physics, University of Science and Technology of China, Anhui, China; 51 Department of Physics, Nanjing University, Jiangsu, China; 52 School of Physics, Shandong University, Shandong, China; 53 Physics Department, Shanghai Jiao Tong University, Shanghai, China; 54 Laboratoire de Physique Corpusculaire, Clermont Université and Université Blaise Pascal and CNRS/IN2P3, Clermont-Ferrand, France; 55 Nevis Laboratory, Columbia University, Irvington, NY United States of America; 56 Niels Bohr Institute, University of Copenhagen, Kobenhavn, Denmark; 57 INFN Gruppo Collegato di Cosenza, Rende, Italy; 58 Dipartimento di Fisica, Università della Calabria, Rende, Italy; 59 AGH University of Science and Technology, Faculty of Physics and Applied Computer Science, Krakow, Poland; 60 The Henryk Niewodniczanski Institute of Nuclear Physics, Polish Academy of Sciences, Krakow, Poland; 61 Physics Department, Southern Methodist University, Dallas, TX United States of America; 62 Physics Department, University of Texas at Dallas, Richardson, TX United States of America; 63 DESY, Hamburg and Zeuthen, Germany; 64 Institut für Experimentelle Physik IV, Technische Universität Dortmund, Dortmund, Germany; 65 Institut für Kern-und Teilchenphysik, Technical University Dresden, Dresden, Germany; 66 Department of Physics, Duke University, Durham, NC United States of America; 67 SUPA - School of Physics and Astronomy, University of Edinburgh, Edinburgh, United Kingdom; 68 INFN Laboratori Nazionali di Frascati, Frascati, Italy; 69 Fakultät für Mathematik und Physik, Albert-Ludwigs-Universität, Freiburg, Germany; 70 Section de Physique, Université de Genève, Geneva, Switzerland; 71 INFN Sezione di Genova, Genova, Italy; 72 Dipartimento di Fisica, Università di Genova, Genova, Italy; 73 E. Andronikashvili Institute of Physics, Iv. Javakhishvili Tbilisi State University, Tbilisi, Georgia; 74 High Energy Physics Institute, Tbilisi State University, Tbilisi, Georgia; 75 II Physikalisches Institut, Justus-Liebig-Universität Giessen, Giessen, Germany; 76 SUPA - School of Physics and Astronomy, University of Glasgow, Glasgow, United Kingdom; 77 II Physikalisches Institut, Georg-August-Universität, Göttingen, Germany; 78 Laboratoire de Physique Subatomique et de Cosmologie, Université Joseph Fourier and CNRS/IN2P3 and Institut National Polytechnique de Grenoble, Grenoble, France; 79 Department of Physics, Hampton University, Hampton, VA United States of America; 80 Laboratory for Particle Physics and Cosmology, Harvard University, Cambridge, MA United States of America; 81 Kirchhoff-Institut für Physik, Ruprecht-Karls-Universität Heidelberg, Heidelberg, Germany; 82 Physikalisches Institut, Ruprecht-Karls-Universität Heidelberg, Heidelberg, Germany; 83 ZITI Institut für technische Informatik, Ruprecht-Karls-Universität Heidelberg, Mannheim, Germany; 84 Faculty of Applied Information Science, Hiroshima Institute of Technology, Hiroshima, Japan; 85 Department of Physics, Indiana University, Bloomington, IN United States of America; 86 Institut für Astro-und Teilchenphysik, Leopold-Franzens-Universität, Innsbruck, Austria; 87 University of Iowa, Iowa City, IA United States of America; 88 Department of Physics and Astronomy, Iowa State University, Ames, IA United States of America; 89 Joint Institute for Nuclear Research, JINR Dubna, Dubna, Russia; 90 KEK, High Energy Accelerator Research Organization, Tsukuba, Japan; 91 Graduate School of Science, Kobe University, Kobe, Japan; 92 Faculty of Science, Kyoto University, Kyoto, Japan; 93 Kyoto University of Education, Kyoto, Japan; 94 Department of Physics, Kyushu University, Fukuoka, Japan; 95 Instituto de Física La Plata, Universidad Nacional de La Plata and CONICET, La Plata, Argentina; 96 Physics Department, Lancaster University, Lancaster, United Kingdom; 97 INFN Sezione di Lecce, Lecce, Italy; 98 Dipartimento di Matematica e Fisica, Università del Salento, Lecce, Italy; 99 Oliver Lodge Laboratory, University of Liverpool, Liverpool, United Kingdom; 100 Department of Physics, Jožef Stefan Institute and University of Ljubljana, Ljubljana, Slovenia; 101 School of Physics and Astronomy, Queen Mary University of London, London, United Kingdom; 102 Department of Physics, Royal Holloway University of London, Surrey, United Kingdom; 103 Department of Physics and Astronomy, University College London, London, United Kingdom; 104 Laboratoire de Physique Nucléaire et de Hautes Energies, UPMC and Université Paris-Diderot and CNRS/IN2P3, Paris, France; 105 Fysiska institutionen, Lunds universitet, Lund, Sweden; 106 Departamento de Fisica Teorica C-15, Universidad Autonoma de Madrid, Madrid, Spain; 107 Institut für Physik, Universität Mainz, Mainz, Germany; 108 School of Physics and Astronomy, University of Manchester, Manchester, United Kingdom; 109 CPPM, Aix-Marseille Université and CNRS/IN2P3, Marseille, France; 110 Department of Physics, University of Massachusetts, Amherst, MA United States of America; 111 Department of Physics, McGill University, Montreal, QC Canada; 112 School of Physics, University of Melbourne, Victoria, Australia; 113 Department of Physics, The University of Michigan, Ann Arbor, MI United States of America; 114 Department of Physics and Astronomy, Michigan State University, East Lansing, MI United States of America; 115 INFN Sezione di Milano, Milano, Italy; 116 Dipartimento di Fisica, Università di Milano, Milano, Italy; 117 B.I. Stepanov Institute of Physics, National Academy of Sciences of Belarus, Minsk, Republic of Belarus; 118 National Scientific and Educational Centre for Particle and High Energy Physics, Minsk, Republic of Belarus; 119 Department of Physics, Massachusetts Institute of Technology, Cambridge, MA United States of America; 120 Group of Particle Physics, University of Montreal, Montreal, QC Canada; 121 P.N. Lebedev Institute of Physics, Academy of Sciences, Moscow, Russia; 122 Institute for Theoretical and Experimental Physics (ITEP), Moscow, Russia; 123 Moscow Engineering and Physics Institute (MEPhI), Moscow, Russia; 124 D.V. Skobeltsyn Institute of Nuclear Physics, M.V. Lomonosov Moscow State University, Moscow, Russia; 125 Fakultät für Physik, Ludwig-Maximilians-Universität München, München, Germany; 126 Max-Planck-Institut für Physik (Werner-Heisenberg-Institut), München, Germany; 127 Nagasaki Institute of Applied Science, Nagasaki, Japan; 128 Graduate School of Science and Kobayashi-Maskawa Institute, Nagoya University, Nagoya, Japan; 129 INFN Sezione di Napoli, Napoli, Italy; 130 Dipartimento di Scienze Fisiche, Università di Napoli, Napoli, Italy; 131 Department of Physics and Astronomy, University of New Mexico, Albuquerque, NM United States of America; 132 Institute for Mathematics, Astrophysics and Particle Physics, Radboud University Nijmegen/Nikhef, Nijmegen, Netherlands; 133 Nikhef National Institute for Subatomic Physics and University of Amsterdam, Amsterdam, Netherlands; 134 Department of Physics, Northern Illinois University, DeKalb, IL United States of America; 135 Budker Institute of Nuclear Physics, SB RAS, Novosibirsk, Russia; 136 Department of Physics, New York University, New York, NY United States of America; 137 Ohio State University, Columbus, OH United States of America; 138 Faculty of Science, Okayama University, Okayama, Japan; 139 Homer L. Dodge Department of Physics and Astronomy, University of Oklahoma, Norman, OK United States of America; 140 Department of Physics, Oklahoma State University, Stillwater, OK United States of America; 141 RCPTM, Palacký University, Olomouc, Czech Republic; 142 Center for High Energy Physics, University of Oregon, Eugene, OR United States of America; 143 LAL, Université Paris-Sud and CNRS/IN2P3, Orsay, France; 144 Graduate School of Science, Osaka University, Osaka, Japan; 145 Department of Physics, University of Oslo, Oslo, Norway; 146 Department of Physics, Oxford University, Oxford, United Kingdom; 147 INFN Sezione di Pavia, Pavia, Italy; 148 Dipartimento di Fisica, Università di Pavia, Pavia, Italy; 149 Department of Physics, University of Pennsylvania, Philadelphia, PA United States of America; 150 Petersburg Nuclear Physics Institute, Gatchina, Russia; 151 INFN Sezione di Pisa, Pisa, Italy; 152 Dipartimento di Fisica E. Fermi, Università di Pisa, Pisa, Italy; 153 Department of Physics and Astronomy, University of Pittsburgh, Pittsburgh, PA United States of America; 154 Laboratorio de Instrumentacao e Fisica Experimental de Particulas - LIP, Lisboa, Portugal; 155 Departamento de Fisica Teorica y del Cosmos and CAFPE, Universidad de Granada, Granada, Spain; 156 Institute of Physics, Academy of Sciences of the Czech Republic, Praha, Czech Republic; 157 Czech Technical University in Prague, Praha, Czech Republic; 158 Faculty of Mathematics and Physics, Charles University in Prague, Praha, Czech Republic; 159 State Research Center Institute for High Energy Physics, Protvino, Russia; 160 Particle Physics Department, Rutherford Appleton Laboratory, Didcot, United Kingdom; 161 Physics Department, University of Regina, Regina, SK Canada; 162 Ritsumeikan University, Kusatsu, Shiga Japan; 163 INFN Sezione di Roma I, Roma, Italy; 164 Dipartimento di Fisica, Università La Sapienza, Roma, Italy; 165 INFN Sezione di Roma Tor Vergata, Roma, Italy; 166 Dipartimento di Fisica, Università di Roma Tor Vergata, Roma, Italy; 167 INFN Sezione di Roma Tre, Roma, Italy; 168 Dipartimento di Fisica, Università Roma Tre, Roma, Italy; 169 Faculté des Sciences Ain Chock, Réseau Universitaire de Physique des Hautes Energies - Université Hassan II, Casablanca, Morocco; 170 Centre National de l’Energie des Sciences Techniques Nucleaires, Rabat, Morocco; 171 Faculté des Sciences Semlalia, Université Cadi Ayyad, LPHEA, Marrakech, Morocco; 172 Faculté des Sciences, Université Mohamed Premier and LPTPM, Oujda, Morocco; 173 Faculté des sciences, Université Mohammed V-Agdal, Rabat, Morocco; 174 DSM/IRFU (Institut de Recherches sur les Lois Fondamentales de l’Univers), CEA Saclay (Commissariat à l’Energie Atomique et aux Energies Alternatives), Gif-sur-Yvette, France; 175 Santa Cruz Institute for Particle Physics, University of California Santa Cruz, Santa Cruz, CA United States of America; 176 Department of Physics, University of Washington, Seattle, WA United States of America; 177 Department of Physics and Astronomy, University of Sheffield, Sheffield, United Kingdom; 178 Department of Physics, Shinshu University, Nagano, Japan; 179 Fachbereich Physik, Universität Siegen, Siegen, Germany; 180 Department of Physics, Simon Fraser University, Burnaby, BC Canada; 181 SLAC National Accelerator Laboratory, Stanford, CA United States of America; 182 Faculty of Mathematics, Physics & Informatics, Comenius University, Bratislava, Slovak Republic; 183 Department of Subnuclear Physics, Institute of Experimental Physics of the Slovak Academy of Sciences, Kosice, Slovak Republic; 184 Department of Physics, University of Johannesburg, Johannesburg, South Africa; 185 School of Physics, University of the Witwatersrand, Johannesburg, South Africa; 186 Department of Physics, Stockholm University, Stockholm, Sweden; 187 The Oskar Klein Centre, Stockholm, Sweden; 188 Physics Department, Royal Institute of Technology, Stockholm, Sweden; 189 Departments of Physics & Astronomy and Chemistry, Stony Brook University, Stony Brook, NY United States of America; 190 Department of Physics and Astronomy, University of Sussex, Brighton, United Kingdom; 191 School of Physics, University of Sydney, Sydney, Australia; 192 Institute of Physics, Academia Sinica, Taipei, Taiwan; 193 Department of Physics, Technion: Israel Institute of Technology, Haifa, Israel; 194 Raymond and Beverly Sackler School of Physics and Astronomy, Tel Aviv University, Tel Aviv, Israel; 195 Department of Physics, Aristotle University of Thessaloniki, Thessaloniki, Greece; 196 International Center for Elementary Particle Physics and Department of Physics, The University of Tokyo, Tokyo, Japan; 197 Graduate School of Science and Technology, Tokyo Metropolitan University, Tokyo, Japan; 198 Department of Physics, Tokyo Institute of Technology, Tokyo, Japan; 199 Department of Physics, University of Toronto, Toronto, ON Canada; 200 TRIUMF, Vancouver, BC Canada; 201 Department of Physics and Astronomy, York University, Toronto, ON Canada; 202 Faculty of Pure and Applied Sciences, University of Tsukuba, Tsukuba, Japan; 203 Department of Physics and Astronomy, Tufts University, Medford, MA United States of America; 204 Centro de Investigaciones, Universidad Antonio Narino, Bogota, Colombia; 205 Department of Physics and Astronomy, University of California Irvine, Irvine, CA United States of America; 206 INFN Gruppo Collegato di Udine, Udine, Italy; 207 ICTP, Trieste, Italy; 208 Dipartimento di Chimica, Fisica e Ambiente, Università di Udine, Udine, Italy; 209 Department of Physics, University of Illinois, Urbana, IL United States of America; 210 Department of Physics and Astronomy, University of Uppsala, Uppsala, Sweden; 211 Instituto de Física Corpuscular (IFIC) and Departamento de Física Atómica, Molecular y Nuclear and Departamento de Ingeniería Electrónica and Instituto de Microelectrónica de Barcelona (IMB-CNM), University of Valencia and CSIC, Valencia, Spain; 212 Department of Physics, University of British Columbia, Vancouver, BC Canada; 213 Department of Physics and Astronomy, University of Victoria, Victoria, BC Canada; 214 Department of Physics, University of Warwick, Coventry, United Kingdom; 215 Waseda University, Tokyo, Japan; 216 Department of Particle Physics, The Weizmann Institute of Science, Rehovot, Israel; 217 Department of Physics, University of Wisconsin, Madison, WI United States of America; 218 Fakultät für Physik und Astronomie, Julius-Maximilians-Universität, Würzburg, Germany; 219 Fachbereich C Physik, Bergische Universität Wuppertal, Wuppertal, Germany; 220 Department of Physics, Yale University, New Haven, CT United States of America; 221 Yerevan Physics Institute, Yerevan, Armenia; 222 Centre de Calcul de l’Institut National de Physique Nucléaire et de Physique des Particules (IN2P3), Villeurbanne, France

## Abstract

The luminosity calibration for the ATLAS detector at the LHC during *pp* collisions at $\sqrt{s} = 7~\mathrm{TeV}$ in 2010 and 2011 is presented. Evaluation of the luminosity scale is performed using several luminosity-sensitive detectors, and comparisons are made of the long-term stability and accuracy of this calibration applied to the *pp* collisions at $\sqrt{s} = 7~\mathrm{TeV}$. A luminosity uncertainty of $\delta\mathcal{L}/ \mathcal{L} = \pm 3.5~\%$ is obtained for the 47 pb^−1^ of data delivered to ATLAS in 2010, and an uncertainty of $\delta\mathcal{L}/ \mathcal{L} = \pm1.8~\%$ is obtained for the 5.5 fb^−1^ delivered in 2011.

## Introduction

An accurate measurement of the delivered luminosity is a key component of the ATLAS [1] physics programme. For cross-section measurements, the uncertainty on the delivered luminosity is often one of the major systematic uncertainties. Searches for, and eventual discoveries of, new physical phenomena beyond the Standard Model also rely on accurate information about the delivered luminosity to evaluate background levels and determine sensitivity to the signatures of new phenomena.

**Fig. 1:**
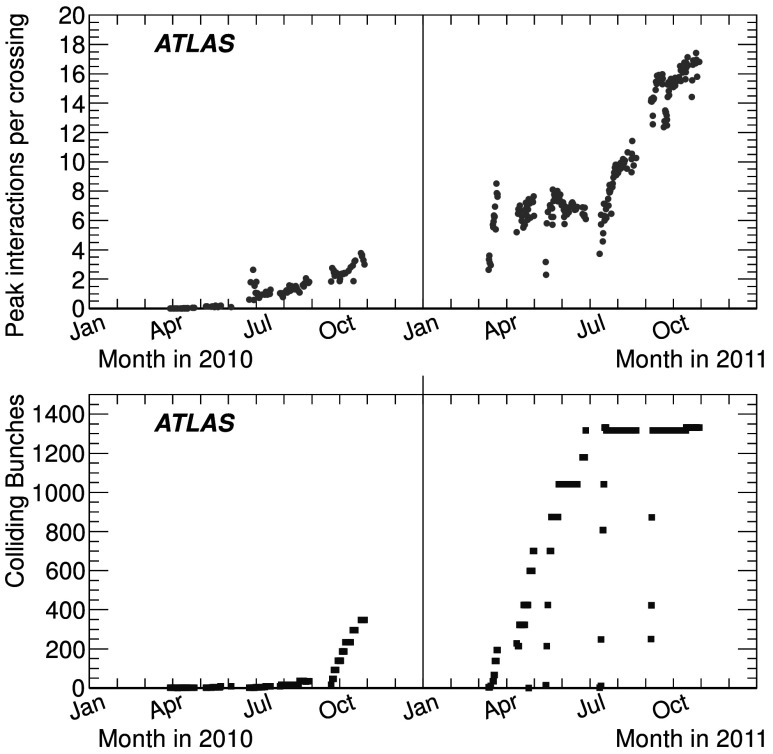
Average number of inelastic *pp* interactions per bunch crossing at the start of each LHC fill (*above*) and number of colliding bunches per LHC fill (*below*) are shown as a function of time in 2010 and 2011. The product of these two quantities is proportional to the peak luminosity at the start of each fill

This paper describes the measurement of the luminosity delivered to the ATLAS detector at the LHC in *pp* collisions at a centre-of-mass energy of $\sqrt{s}=7~\mathrm{TeV}$ during 2010 and 2011. The analysis is an evolution of the process documented in the initial ATLAS luminosity publication [2] and includes an improved determination of the luminosity in 2010 along with a new analysis for 2011. Table 1 highlights the operational conditions of the LHC during 2010 and 2011. The peak instantaneous luminosity delivered by the LHC at the start of a fill increased from $\mathcal{L}_{\mathrm{peak}} = 2.0 \times10^{32}\ \mathrm{cm}^{-2}\,\mathrm{s}^{-1}$ in 2010 to $\mathcal{L}_{\mathrm{peak}} = 3.6 \times10^{33}\ \mathrm{cm}^{-2}\,\mathrm{s}^{-1}$ by the end of 2011. This increase results from both an increased instantaneous luminosity delivered per bunch crossing as well as a significant increase in the total number of bunches colliding. Figure 1 illustrates the evolution of these two parameters as a function of time. As a result of these changes in operating conditions, the details of the luminosity measurement have evolved from 2010 to 2011, although the overall methodology remains largely the same.

**Table 1:** Selected LHC parameters for *pp* collisions at $\sqrt{s} = 7~\mathrm{TeV}$ in 2010 and 2011. Parameters shown are the best achieved for that year in normal physics operations

Parameter	2010	2011
Maximum number of bunch pairs colliding	348	1331
Minimum bunch spacing (ns)	150	50
Typical bunch population (10^11^ protons)	0.9	1.2
Peak luminosity (10^33^ cm^−2^ s^−1^)	0.2	3.6
Maximum inelastic interactions per crossing	∼5	∼20
Total integrated luminosity delivered	47 pb^−1^	5.5 fb^−1^

The strategy for measuring and calibrating the luminosity is outlined in Sect. 2, followed in Sect. 3 by a brief description of the detectors used for luminosity determination. Each of these detectors utilizes one or more luminosity algorithms as described in Sect. 4. The absolute calibration of these algorithms using beam-separation scans is described in Sect. 5, while a summary of the systematic uncertainties on the luminosity calibration as well as the calibration results are presented in Sect. 6. Additional corrections which must be applied over the course of the 2011 data-taking period are described in Sect. 7, while additional uncertainties related to the extrapolation of the absolute luminosity calibration to the full 2010 and 2011 data samples are described in Sect. 8. The final results and uncertainties are summarized in Sect. 9.

## Overview

The luminosity $\mathcal{L}$ of a *pp* collider can be expressed as

1 $$ \mathcal{L} = \frac{R_{\mathrm{inel}}}{\sigma_{\mathrm{inel}}} $$

where *R*_inel_ is the rate of inelastic collisions and *σ*_inel_ is the *pp* inelastic cross-section. For a storage ring, operating at a revolution frequency *f*_r_ and with *n*_b_ bunch pairs colliding per revolution, this expression can be rewritten as

2 $$ \mathcal{L} = \frac{{\mu n_\mathrm{b} f_\mathrm{r} }}{{\sigma_{\mathrm{inel}} }} $$

where *μ* is the average number of inelastic interactions per bunch crossing.

As discussed in Sects. 3 and 4, ATLAS monitors the delivered luminosity by measuring the observed interaction rate per crossing, *μ*_vis_, independently with a variety of detectors and using several different algorithms. The luminosity can then be written as

3 $$ \mathcal{L} = \frac{{\mu_{\mathrm{vis}} n_{\mathrm{b}} f_{\mathrm{r}} }}{{\sigma_{\mathrm{vis}} }} $$

where *σ*_vis_=*εσ*_inel_ is the total inelastic cross-section multiplied by the efficiency *ε* of a particular detector and algorithm, and similarly *μ*_vis_=*εμ*. Since *μ*_vis_ is an experimentally observable quantity, the calibration of the luminosity scale for a particular detector and algorithm is equivalent to determining the visible cross-section *σ*_vis_.

The majority of the algorithms used in the ATLAS luminosity determination are *event counting* algorithms, where each particular bunch crossing is categorized as either passing or not passing a given set of criteria designed to detect the presence of at least one inelastic *pp* collision. In the limit *μ*_vis_≪1, the average number of visible inelastic interactions per bunch crossing is given by the simple expression *μ*_vis_≈*N*/*N*_BC_ where *N* is the number of bunch crossings (or events) passing the selection criteria that are observed during a given time interval, and *N*_BC_ is the total number of bunch crossings in that same interval. As *μ*_vis_ increases, the probability that two or more *pp* interactions occur in the same bunch crossing is no longer negligible (a condition referred to as “pile-up”), and *μ*_vis_ is no longer linearly related to the raw event count *N*. Instead *μ*_vis_ must be calculated taking into account Poisson statistics, and in some cases instrumental or pile-up-related effects. In the limit where all bunch crossings in a given time interval contain an event, the event counting algorithm no longer provides any useful information about the interaction rate.

An alternative approach, which is linear to higher values of *μ*_vis_ but requires control of additional systematic effects, is that of *hit counting* algorithms. Rather than counting how many bunch crossings pass some minimum criteria for containing at least one inelastic interaction, in hit counting algorithms the number of detector readout channels with signals above some predefined threshold is counted. This provides more information per event, and also increases the *μ*_vis_ value at which the algorithm saturates compared to an event-counting algorithm. The extreme limit of hit counting algorithms, achievable only in detectors with very fine segmentation, are *particle counting* algorithms, where the number of individual particles entering a given detector is counted directly. More details on how these different algorithms are defined, as well as the procedures for converting the observed event or hit rate into the visible interaction rate *μ*_vis_, are discussed in Sect. 4.

As described more fully in Sect. 5, the calibration of *σ*_vis_ is performed using dedicated beam-separation scans, also known as van der Meer (*vdM*) scans, where the absolute luminosity can be inferred from direct measurements of the beam parameters [3, 4]. The delivered luminosity can be written in terms of the accelerator parameters as

4 $$ {\mathcal{L}} = \frac{{n_{\mathrm{b}} f_{\mathrm{r}} n_1 n_2 }}{{2\pi \varSigma_x \varSigma_y }} $$

where *n*_1_ and *n*_2_ are the bunch populations (protons per bunch) in beam 1 and beam 2 respectively (together forming the bunch population product), and *Σ*_*x*_ and *Σ*_*y*_ characterize the horizontal and vertical convolved beam widths. In a *vdM* scan, the beams are separated by steps of a known distance, which allows a direct measurement of *Σ*_*x*_ and *Σ*_*y*_. Combining this scan with an external measurement of the bunch population product *n*_1_ *n*_2_ provides a direct determination of the luminosity when the beams are unseparated.

A fundamental ingredient of the ATLAS strategy to assess and control the systematic uncertainties affecting the absolute luminosity determination is to compare the measurements of several luminosity detectors, most of which use more than one algorithm to assess the luminosity. These multiple detectors and algorithms are characterized by significantly different acceptance, response to pile-up, and sensitivity to instrumental effects and to beam-induced backgrounds. In particular, since the calibration of the absolute luminosity scale is established in dedicated *vdM* scans which are carried out relatively infrequently (in 2011 there was only one set of *vdM* scans at $\sqrt{s} = 7~\mathrm{TeV}$ for the entire year), this calibration must be assumed to be constant over long periods and under different machine conditions. The level of consistency across the various methods, over the full range of single-bunch luminosities and beam conditions, and across many months of LHC operation, provides valuable cross-checks as well as an estimate of the detector-related systematic uncertainties. A full discussion of these is presented in Sects. 6–8.

The information needed for most physics analyses is an integrated luminosity for some well-defined data sample. The basic time unit for storing luminosity information for physics use is the Luminosity Block (LB). The boundaries of each LB are defined by the ATLAS Central Trigger Processor (CTP), and in general the duration of each LB is one minute. Trigger configuration changes, such as prescale changes, can only happen at luminosity block boundaries, and data are analysed under the assumption that each luminosity block contains data taken under uniform conditions, including luminosity. The average luminosity for each detector and algorithm, along with a variety of general ATLAS data quality information, is stored for each LB in a relational database. To define a data sample for physics, quality criteria are applied to select LBs where conditions are acceptable, then the average luminosity in that LB is multiplied by the LB duration to provide the integrated luminosity delivered in that LB. Additional corrections can be made for trigger deadtime and trigger prescale factors, which are also recorded on a per-LB basis. Adding up the integrated luminosity delivered in a specific set of luminosity blocks provides the integrated luminosity of the entire data sample.

## Luminosity detectors

This section provides a description of the detector subsystems used for luminosity measurements. The ATLAS detector is discussed in detail in Ref. [1]. The first set of detectors uses either event or hit counting algorithms to measure the luminosity on a bunch-by-bunch basis. The second set infers the total luminosity (summed over all bunches) by monitoring detector currents sensitive to average particle rates over longer time scales. In each case, the detector descriptions are arranged in order of increasing magnitude of pseudorapidity.^1^

The Inner Detector is used to measure the momentum of charged particles over a pseudorapidity interval of |*η*|<2.5. It consists of three subsystems: a pixel detector, a silicon microstrip tracker, and a transition-radiation straw-tube tracker. These detectors are located inside a solenoidal magnet that provides a 2 T axial field. The tracking efficiency as a function of transverse momentum (*p*_T_), averaged over all pseudorapidity, rises from 10 % at 100 MeV to around 86 % for *p*_T_ above a few GeV [5, 6]. The main application of the Inner Detector for luminosity measurements is to detect the primary vertices produced in inelastic *pp* interactions.

To provide efficient triggers at low instantaneous luminosity ($\mathcal {L} < 10^{33}~{\rm cm}^{-2}\,{\rm s}^{-1}$), ATLAS has been equipped with segmented scintillator counters, the Minimum Bias Trigger Scintillators (MBTS). Located at *z*=±365 cm from the nominal interaction point (IP), and covering a rapidity range 2.09<|*η*|<3.84, the main purpose of the MBTS system is to provide a trigger on minimum collision activity during a *pp* bunch crossing. Light emitted by the scintillators is collected by wavelength-shifting optical fibers and guided to photomultiplier tubes. The MBTS signals, after being shaped and amplified, are fed into leading-edge discriminators and sent to the trigger system. The MBTS detectors are primarily used for luminosity measurements in early 2010, and are no longer used in the 2011 data.

The Beam Conditions Monitor (BCM) consists of four small diamond sensors, approximately 1 cm^2^ in cross-section each, arranged around the beampipe in a cross pattern on each side of the IP, at a distance of *z*=±184 cm. The BCM is a fast device originally designed to monitor background levels and issue beam-abort requests when beam losses start to risk damaging the Inner Detector. The fast readout of the BCM also provides a bunch-by-bunch luminosity signal at |*η*|=4.2 with a time resolution of ≃0.7 ns. The horizontal and vertical pairs of BCM detectors are read out separately, leading to two luminosity measurements labelled BCMH and BCMV respectively. Because the acceptances, thresholds, and data paths may all have small differences between BCMH and BCMV, these two measurements are treated as being made by independent devices for calibration and monitoring purposes, although the overall response of the two devices is expected to be very similar. In the 2010 data, only the BCMH readout is available for luminosity measurements, while both BCMH and BCMV are available in 2011.

LUCID is a Cherenkov detector specifically designed for measuring the luminosity. Sixteen mechanically polished aluminium tubes filled with ${\rm C}_{4}{\rm F}_{10}$ gas surround the beampipe on each side of the IP at a distance of 17 m, covering the pseudorapidity range 5.6<|*η*|<6.0. The Cherenkov photons created by charged particles in the gas are reflected by the tube walls until they reach photomultiplier tubes (PMTs) situated at the back end of the tubes. Additional Cherenkov photons are produced in the quartz window separating the aluminium tubes from the PMTs. The Cherenkov light created in the gas typically produces 60–70 photoelectrons per incident charged particle, while the quartz window adds another 40 photoelectrons to the signal. If one of the LUCID PMTs produces a signal over a preset threshold (equivalent to ≃15 photoelectrons), a “hit” is recorded for that tube in that bunch crossing. The LUCID hit pattern is processed by a custom-built electronics card which contains Field Programmable Gate Arrays (FPGAs). This card can be programmed with different luminosity algorithms, and provides separate luminosity measurements for each LHC bunch crossing.

Both BCM and LUCID are fast detectors with electronics capable of making statistically precise luminosity measurements separately for each bunch crossing within the LHC fill pattern with no deadtime. These FPGA-based front-end electronics run autonomously from the main data acquisition system, and in particular are not affected by any deadtime imposed by the CTP.^2^

The Inner Detector vertex data and the MBTS data are components of the events read out through the data acquisition system, and so must be corrected for deadtime imposed by the CTP in order to measure delivered luminosity. Normally this deadtime is below 1 %, but can occasionally be larger. Since not every inelastic collision event can be read out through the data acquisition system, the bunch crossings are sampled with a random or minimum bias trigger. While the triggered events uniformly sample every bunch crossing, the trigger bandwidth devoted to random or minimum bias triggers is not large enough to measure the luminosity separately for each bunch pair in a given LHC fill pattern during normal physics operations. For special running conditions such as the *vdM* scans, a custom trigger with partial event readout has been introduced in 2011 to record enough events to allow bunch-by-bunch luminosity measurements from the Inner Detector vertex data.

In addition to the detectors listed above, further luminosity-sensitive methods have been developed which use components of the ATLAS calorimeter system. These techniques do not identify particular events, but rather measure average particle rates over longer time scales.

The Tile Calorimeter (TileCal) is the central hadronic calorimeter of ATLAS. It is a sampling calorimeter constructed from iron plates (absorber) and plastic tile scintillators (active material) covering the pseudorapidity range |*η*|<1.7. The detector consists of three cylinders, a central long barrel and two smaller extended barrels, one on each side of the long barrel. Each cylinder is divided into 64 slices in *ϕ* (modules) and segmented into three radial sampling layers. Cells are defined in each layer according to a projective geometry, and each cell is connected by optical fibers to two photomultiplier tubes. The current drawn by each PMT is monitored by an integrator system which is sensitive to currents from 0.1 nA to 1.2 mA with a time constant of 10 ms. The current drawn is proportional to the total number of particles interacting in a given TileCal cell, and provides a signal proportional to the total luminosity summed over all the colliding bunches present at a given time.

The Forward Calorimeter (FCal) is a sampling calorimeter that covers the pseudorapidity range 3.2<|*η*|<4.9 and is housed in the two endcap cryostats along with the electromagnetic endcap and the hadronic endcap calorimeters. Each of the two FCal modules is divided into three longitudinal absorber matrices, one made of copper (FCal-1) and the other two of tungsten (FCal-2/3). Each matrix contains tubes arranged parallel to the beam axis filled with liquid argon as the active medium. Each FCal-1 matrix is divided into 16 $\rm\phi$-sectors, each of them fed by four independent high-voltage lines. The high voltage on each sector is regulated to provide a stable electric field across the liquid argon gaps and, similar to the TileCal PMT currents, the currents provided by the FCal-1 high-voltage system are directly proportional to the average rate of particles interacting in a given FCal sector.

## Luminosity algorithms

This section describes the algorithms used by the luminosity-sensitive detectors described in Sect. 3 to measure the visible interaction rate per bunch crossing, *μ*_vis_. Most of the algorithms used do not measure *μ*_vis_ directly, but rather measure some other rate which can be used to determine *μ*_vis_.

ATLAS primarily uses event counting algorithms to measure luminosity, where a bunch crossing is said to contain an “event” if the criteria for a given algorithm to observe one or more interactions are satisfied. The two main algorithm types being used are EventOR (inclusive counting) and EventAND (coincidence counting). Additional algorithms have been developed using hit counting and average particle rate counting, which provide a cross-check of the linearity of the event counting techniques.

### Interaction rate determination

Most of the primary luminosity detectors consist of two symmetric detector elements placed in the forward (“A”) and backward (“C”) direction from the interaction point. For the LUCID, BCM, and MBTS detectors, each side is further segmented into a discrete number of readout segments, typically arranged azimuthally around the beampipe, each with a separate readout channel. For event counting algorithms, a threshold is applied to the analoge signal output from each readout channel, and every channel with a response above this threshold is counted as containing a “hit”.

In an EventOR algorithm, a bunch crossing is counted if there is at least one hit on either the A side or the C side. Assuming that the number of interactions in a bunch crossing can be described by a Poisson distribution, the probability of observing an OR event can be computed as 5 Here the raw event count *N*_OR_ is the number of bunch crossings, during a given time interval, in which at least one *pp* interaction satisfies the event-selection criteria of the OR algorithm under consideration, and *N*_BC_ is the total number of bunch crossings during the same interval. Solving for *μ*_vis_ in terms of the event counting rate yields:

6 $$ \mu_{\mathrm{vis}}^{\mathrm{OR}} = - \ln \biggl( 1- \frac{N_{\mathrm{OR}}}{N_{\mathrm{BC}}} \biggr). $$

In the case of an EventAND algorithm, a bunch crossing is counted if there is at least one hit on both sides of the detector. This coincidence condition can be satisfied either from a single *pp* interaction or from individual hits on either side of the detector from different *pp* interactions in the same bunch crossing. Assuming equal acceptance for sides A and C, the probability of recording an AND event can be expressed as 7 This relationship cannot be inverted analytically to determine $\mu _{\mathrm{vis}}^{\mathrm{AND}}$ as a function of *N*_AND_/*N*_BC_ so a numerical inversion is performed instead.

When *μ*_vis_≫1, event counting algorithms lose sensitivity as fewer and fewer events in a given time interval have bunch crossings with zero observed interactions. In the limit where *N*/*N*_BC_=1, it is no longer possible to use event counting to determine the interaction rate *μ*_vis_, and more sophisticated techniques must be used. One example is a *hit counting* algorithm, where the number of hits in a given detector is counted rather than just the total number of events. This provides more information about the interaction rate per event, and increases the luminosity at which the algorithm saturates.

Under the assumption that the number of hits in one *pp* interaction follows a Binomial distribution and that the number of interactions per bunch crossing follows a Poisson distribution, one can calculate the average probability to have a hit in one of the detector channels per bunch crossing as 8 where *N*_HIT_ and *N*_BC_ are the total numbers of hits and bunch crossings during a time interval, and *N*_CH_ is the number of detector channels. The expression above enables $\mu_{\mathrm{vis}}^{\mathrm{HIT}}$ to be calculated from the number of hits as

9 $$ \mu_\mathrm{vis}^{\mathrm{HIT}} = -\ln\biggl(1-\frac{N_\mathrm{HIT}}{N_\mathrm{BC}N_\mathrm{CH}}\biggr). $$

Hit counting is used to analyse the LUCID response (*N*_CH_=30) only in the high-luminosity data taken in 2011. The lower acceptance of the BCM detector allows event counting to remain viable for all of 2011. The binomial assumption used to derive Eq. (9) is only true if the probability to observe a hit in a single channel is independent of the number of hits observed in the other channels. A study of the LUCID hit distributions shows that this is not a correct assumption, although the data presented in Sect. 8 also show that Eq. (9) provides a good description of how $\mu_{\mathrm{vis}}^{\mathrm{HIT}}$ depends on the average number of hits.

An additional type of algorithm that can be used is a *particle counting* algorithm, where some observable is directly proportional to the number of particles interacting in the detector. These should be the most linear of all of the algorithm types, and in principle the interaction rate is directly proportional to the particle rate. As discussed below, the TileCal and FCal current measurements are not exactly particle counting algorithms, as individual particles are not counted, but the measured currents should be directly proportional to luminosity. Similarly, the number of primary vertices is directly proportional to the luminosity, although the vertex reconstruction efficiency is significantly affected by pile-up as discussed below.

### Online algorithms

The two main luminosity detectors used are LUCID and BCM. Each of these is equipped with customized FPGA-based readout electronics which allow the luminosity algorithms to be applied “online” in real time. These electronics provide fast diagnostic signals to the LHC (within a few seconds), in addition to providing luminosity measurements for physics use. Each colliding bunch pair can be identified numerically by a Bunch-Crossing Identifier (BCID) which labels each of the 3564 possible 25 ns slots in one full revolution of the nominal LHC fill pattern. The online algorithms measure the delivered luminosity independently in each BCID.

For the LUCID detector, the two main algorithms are the inclusive LUCID_EventOR and the coincidence LUCID_EventAND. In each case, a hit is defined as a PMT signal above a predefined threshold which is set lower than the average single-particle response. There are two additional algorithms defined, LUCID_EventA and LUCID_EventC, which require at least one hit on either the A or C side respectively. Events passing these LUCID_EventA and LUCID_EventC algorithms are subsets of the events passing the LUCID_EventOR algorithm, and these single-sided algorithms are used primarily to monitor the stability of the LUCID detector. There is also a LUCID_HitOR hit counting algorithm which has been employed in the 2011 running to cross-check the linearity of the event counting algorithms at high values of *μ*_vis_.

For the BCM detector, there are two independent readout systems (BCMH and BCMV). A hit is defined as a single sensor with a response above the noise threshold. Inclusive OR and coincidence AND algorithms are defined for each of these independent readout systems, for a total of four BCM algorithms.

### Offline algorithms

Additional offline analyses have been performed which rely on the MBTS and the vertexing capabilities of the Inner Detector. These offline algorithms use data triggered and read out through the standard ATLAS data acquisition system, and do not have the necessary rate capability to measure luminosity independently for each BCID under normal physics conditions. Instead, these algorithms are typically used as cross-checks of the primary online algorithms under special running conditions, where the trigger rates for these algorithms can be increased.

The MBTS system is used for luminosity measurements only for the data collected in the 2010 run before 150 ns bunch train operation began. Events are triggered by the L1_MBTS_1 trigger which requires at least one hit in any of the 32 MBTS counters (which is equivalent to an inclusive MBTS_EventOR requirement). In addition to the trigger requirement, the MBTS_Timing analysis uses the time measurement of the MBTS detectors to select events where the time difference between the average hit times on the two sides of the MBTS satisfies |*Δt*|<10 ns. This requirement is effective in rejecting beam-induced background events, as the particles produced in these events tend to traverse the detector longitudinally resulting in large values of |*Δt*|, while particles coming from the interaction point produce values of |*Δt*|≃0. To form a *Δt* value requires at least one hit on both sides of the IP, and so the MBTS_Timing algorithm is in fact a coincidence algorithm.

Additional algorithms have been developed which are based on reconstructing interaction vertices formed by tracks measured in the Inner Detector. In 2010, the events were triggered by the L1_MBTS_1 trigger. The 2010 algorithm counts events with at least one reconstructed vertex, with at least two tracks with *p*_T_>100 MeV. This “primary vertex event counting” (PrimVtx) algorithm is fundamentally an inclusive event-counting algorithm, and the conversion from the observed event rate to *μ*_vis_ follows Eq. (5).

The 2011 vertexing algorithm uses events from a trigger which randomly selects crossings from filled bunch pairs where collisions are possible. The average number of visible interactions per bunch crossing is determined by counting the number of reconstructed vertices found in each bunch crossing (Vertex). The vertex selection criteria in 2011 were changed to require five tracks with *p*_T_>400 MeV while also requiring tracks to have a hit in any active pixel detector module along their path.

Vertex counting suffers from nonlinear behaviour with increasing interaction rates per bunch crossing, primarily due to two effects: vertex masking and fake vertices. Vertex masking occurs when the vertex reconstruction algorithm fails to resolve nearby vertices from separate interactions, decreasing the vertex reconstruction efficiency as the interaction rate increases. A data-driven correction is derived from the distribution of distances in the longitudinal direction (*Δz*) between pairs of reconstructed vertices. The measured distribution of longitudinal positions (*z*) is used to predict the expected *Δz* distribution of pairs of vertices if no masking effect was present. Then, the difference between the expected and observed *Δz* distributions is related to the number of vertices lost due to masking. The procedure is checked with simulation for self-consistency at the sub-percent level, and the magnitude of the correction reaches up to +50 % over the range of pile-up values in 2011 physics data. Fake vertices result from a vertex that would normally fail the requirement on the minimum number of tracks, but additional tracks from a second nearby interaction are erroneously assigned so that the resulting reconstructed vertex satisfies the selection criteria. A correction is derived from simulation and reaches −10 % in 2011. Since the 2010 PrimVtx algorithm requirements are already satisfied with one reconstructed vertex, vertex masking has no effect, although a correction must still be made for fake vertices.

### Calorimeter-based algorithms

The TileCal and FCal luminosity determinations do not depend upon event counting, but rather upon measuring detector currents that are proportional to the total particle flux in specific regions of the calorimeters. These particle counting algorithms are expected to be free from pile-up effects up to the highest interaction rates observed in late 2011 (*μ*≃20).

The Tile luminosity algorithm measures PMT currents for selected cells in a region near |*η*|≈1.25 where the largest variations in current as a function of the luminosity are observed. In 2010, the response of a common set of cells was calibrated with respect to the luminosity measured by the LUCID_EventOR algorithm in a single ATLAS run. At the higher luminosities encountered in 2011, TileCal started to suffer from frequent trips of the low-voltage power supplies, causing the intermittent loss of current measurements from several modules. For these data, a second method is applied, based on the calibration of individual cells, which has the advantage of allowing different sets of cells to be used depending on their availability at a given time. The calibration is performed by comparing the luminosity measured by the LUCID_EventOR algorithm to the individual cell currents at the peaks of the 2011 *vdM* scan, as more fully described in Sect. 7.5. While TileCal does not provide an independent absolute luminosity measurement, it enables systematic uncertainties associated with both long-term stability and *μ*-dependence to be evaluated.

Similarly, the FCal high-voltage currents cannot be directly calibrated during a *vdM* scan because the total luminosity delivered in these scans remains below the sensitivity of the current-measurement technique. Instead, calibrations were evaluated for each usable HV line independently by comparing to the LUCID_EventOR luminosity for a single ATLAS run in each of 2010 and 2011. As a result, the FCal also does not provide an independently calibrated luminosity measurement, but it can be used as a systematic check of the stability and linearity of other algorithms. For both the TileCal and FCal analyses, the luminosity is assumed to be linearly proportional to the observed currents after correcting for pedestals and non-collision backgrounds.

## Luminosity calibration

In order to use the measured interaction rate *μ*_vis_ as a luminosity monitor, each detector and algorithm must be calibrated by determining its visible cross-section *σ*_vis_. The primary calibration technique to determine the absolute luminosity scale of each luminosity detector and algorithm employs dedicated *vdM* scans to infer the delivered luminosity at one point in time from the measurable parameters of the colliding bunches. By comparing the known luminosity delivered in the *vdM* scan to the visible interaction rate *μ*_vis_, the visible cross-section can be determined from Eq. (3).

To achieve the desired accuracy on the absolute luminosity, these scans are not performed during normal physics operations, but rather under carefully controlled conditions with a limited number of colliding bunches and a modest peak interaction rate (*μ*≲2). At $\sqrt{s} = 7~\mathrm{TeV}$, three sets of such scans were performed in 2010 and one set in 2011. This section describes the *vdM* scan procedure, while Sect. 6 discusses the systematic uncertainties on this procedure and summarizes the calibration results.

### Absolute luminosity from beam parameters

In terms of colliding-beam parameters, the luminosity $\mathcal{L}$ is defined (for beams colliding with zero crossing angle) as

10 $$ {\mathcal{L}} = n_{\mathrm{b}} f_{\mathrm{r}} n_1 n_2 \int{\hat{\rho} _1 (x,y)} \hat{\rho} _2 (x,y)\,dx\,dy $$

where *n*_b_ is the number of colliding bunch pairs, *f*_r_ is the machine revolution frequency (11245.5 Hz for the LHC), *n*_1_ *n*_2_ is the bunch population product, and $\hat{\rho}_{1(2)}(x,y)$ is the normalized particle density in the transverse (*x*–*y*) plane of beam 1 (2) at the IP. Under the general assumption that the particle densities can be factorized into independent horizontal and vertical components, ($\hat{\rho}(x,y)=\rho_{x}(x)\rho_{y}(y)$), Eq. (10) can be rewritten as

11 $$ {\mathcal{L}} = n_{\mathrm{b}} f_{\mathrm{r}} n_1 n_2 ~\varOmega_x (\rho_{x1}, \rho_{x2}) ~\varOmega_y (\rho_{y1}, \rho_{y2}) $$

where

$$\varOmega_x (\rho_{x1},\rho_{x2} ) = \int{ \rho_{x1} (x)}\rho_{x2} (x)\,dx $$

is the beam-overlap integral in the *x* direction (with an analogous definition in the *y* direction). In the method proposed by van der Meer [3] the overlap integral (for example in the *x* direction) can be calculated as

12 $$ \varOmega_x (\rho_{x1},\rho_{x2}) = \frac{{R_x (0)}}{{\int{R_x (\delta)\,d\delta} }}, $$

where *R*_*x*_(*δ*) is the luminosity (or equivalently *μ*_vis_)—at this stage in arbitrary units—measured during a horizontal scan at the time the two beams are separated by the distance *δ*, and *δ*=0 represents the case of zero beam separation.

Defining the parameter *Σ*_*x*_ as

13 $$ \varSigma_x = \frac{1}{{\sqrt{2\pi} }}\frac{{\int{R_x (\delta)\,d\delta} }}{{R_x (0)}}, $$

and similarly for *Σ*_*y*_, the luminosity in Eq. (11) can be rewritten as

14 $$ {\mathcal{L}} = \frac{{n_{\mathrm{b}} f_{\mathrm{r}} n_1 n_2 }}{{2\pi \varSigma_x \varSigma_y }}, $$

which enables the luminosity to be extracted from machine parameters by performing a *vdM* (beam-separation) scan. In the case where the luminosity curve *R*_*x*_(*δ*) is Gaussian, *Σ*_*x*_ coincides with the standard deviation of that distribution. Equation (14) is quite general; *Σ*_*x*_ and *Σ*_*y*_, as defined in Eq. (13), depend only upon the area under the luminosity curve, and make no assumption as to the shape of that curve.

### *vdM* scan calibration

To calibrate a given luminosity algorithm, one can equate the absolute luminosity computed using Eq. (14) to the luminosity measured by a particular algorithm at the peak of the scan curve using Eq. (3) to get

15 $$ \sigma_{\mathrm{vis}} =\mu^{\mathrm{MAX}}_{\mathrm{vis}} \frac{2\pi \varSigma_x \varSigma_y}{n_1 n_2}, $$

where $\mu^{\mathrm{MAX}}_{\mathrm{vis}}$ is the visible interaction rate per bunch crossing observed at the peak of the scan curve as measured by that particular algorithm. Equation (15) provides a direct calibration of the visible cross-section *σ*_vis_ for each algorithm in terms of the peak visible interaction rate $\mu^{\mathrm{MAX}}_{\mathrm{vis}}$, the product of the convolved beam widths *Σ*_*x*_ *Σ*_*y*_, and the bunch population product *n*_1_ *n*_2_. As discussed below, the bunch population product must be determined from an external analysis of the LHC beam currents, but the remaining parameters are extracted directly from the analysis of the *vdM* scan data.

For scans performed with a crossing angle, where the beams no longer collide head-on, the formalism becomes considerably more involved [7], but the conclusions remain unaltered and Eqs. (13)–(15) remain valid. The non-zero vertical crossing angle used for some scans widens the luminosity curve by a factor that depends on the bunch length, the transverse beam size and the crossing angle, but reduces the peak luminosity by the same factor. The corresponding increase in the measured value of *Σ*_*y*_ is exactly cancelled by the decrease in $\mu^{\mathrm{MAX}}_{\mathrm{vis}}$, so that no correction for the crossing angle is needed in the determination of *σ*_vis_.

One useful quantity that can be extracted from the *vdM* scan data for each luminosity method and that depends only on the transverse beam sizes, is the specific luminosity $\mathcal{L}_{\mathrm{spec}}$:

16 $$ \mathcal{L}_{\mathrm{spec}} = \mathcal{L}/(n_{\mathrm{b}} n_1 n_2) = \frac{f_{\mathrm{r}}}{2\pi\varSigma_x \varSigma_y}. $$

Comparing the specific luminosity values (i.e. the inverse product of the convolved beam sizes) measured in the same scan by different detectors and algorithms provides a direct check on the mutual consistency of the absolute luminosity scale provided by these methods.

### *vdM* scan data sets

The beam conditions during the dedicated *vdM* scans are different from the conditions in normal physics fills, with fewer bunches colliding, no bunch trains, and lower bunch intensities. These conditions are chosen to reduce various systematic uncertainties in the scan procedure.

A total of five *vdM* scans were performed in 2010, on three different dates separated by weeks or months, and an additional two *vdM* scans at $\sqrt{s} = 7~\mathrm{TeV}$ were performed in 2011 on the same day to calibrate the absolute luminosity scale. As shown in Table 2, the scan parameters evolved from the early 2010 scans where single bunches and very low bunch charges were used. The final set of scans in 2010 and the scans in 2011 were more similar, as both used close-to-nominal bunch charges, more than one bunch colliding, and typical peak *μ* values in the range 1.3–2.3.

**Table 2:** Summary of the main characteristics of the 2010 and 2011 *vdM* scans performed at the ATLAS interaction point. Scan directions are indicated by “H” for horizontal and “V” for vertical. The values of luminosity/bunch and *μ* are given for zero beam separation

Scan Number	I	II–III	IV–V	VII–IX
LHC Fill Number	1059	1089	1386	1783
Date	26 Apr., 2010	9 May, 2010	1 Oct., 2010	15 May, 2011
Scan Directions	1 H scan followed by 1 V scan	2 H scans followed by 2 V scans	2 sets of H plus V scans	3 sets of H plus V scans (scan IX offset)
Total Scan Steps per Plane	27 (±6*σ*_b_)	27 (±6*σ*_b_)	25 (±6*σ*_b_)	25 (±6*σ*_b_)
Scan Duration per Step	30 s	30 s	20 s	20 s
Bunches colliding in ATLAS & CMS	1	1	6	14
Total number of bunches per beam	2	2	19	38
Typical number of protons per bunch (×10^11^)	0.1	0.2	0.9	0.8
Nominal *β*-function at IP [*β* ^⋆^] (m)	2	2	3.5	1.5
Approx. transverse single beam size *σ*_b_ (μm)	45	45	57	40
Nominal half crossing angle (μrad)	0	0	±100	±120
Typical luminosity/bunch ()	4.5⋅10^−3^	1.8⋅10^−2^	0.22	0.38
*μ* (interactions/crossing)	0.03	0.11	1.3	2.3

Generally, each *vdM* scan consists of two separate beam scans, one where the beams are separated by up to ±6*σ*_b_ in the *x* direction keeping the beams centred in *y*, and a second where the beams are separated in the *y* direction with the beams centred in *x*, where *σ*_b_ is the transverse size of a single beam. The beams are moved in a certain number of scan steps, then data are recorded for 20–30 seconds at each step to obtain a statistically significant measurement in each luminosity detector under calibration. To help assess experimental systematic uncertainties in the calibration procedure, two sets of identical *vdM* scans are usually taken in short succession to provide two independent calibrations under similar beam conditions. In 2011, a third scan was performed with the beams separated by 160 μm in the non-scanning plane to constrain systematic uncertainties on the factorization assumption as discussed in Sect. 6.1.11.

Since the luminosity can be different for each colliding bunch pair, both because the beam sizes can vary bunch-to-bunch but also because the bunch population product *n*_1_ *n*_2_ can vary at the level of 10–20 %, the determination of *Σ*_*x*/*y*_ and the measurement of $\mu^{\mathrm{MAX}}_{\mathrm{vis}}$ at the scan peak must be performed independently for each colliding BCID. As a result, the May 2011 scan provides 14 independent measurements of *σ*_vis_ within the same scan, and the October 2010 scan provides 6. The agreement among the *σ*_vis_ values extracted from these different BCIDs provides an additional consistency check for the calibration procedure.

### *vdM* scan analysis

**Fig. 2:**
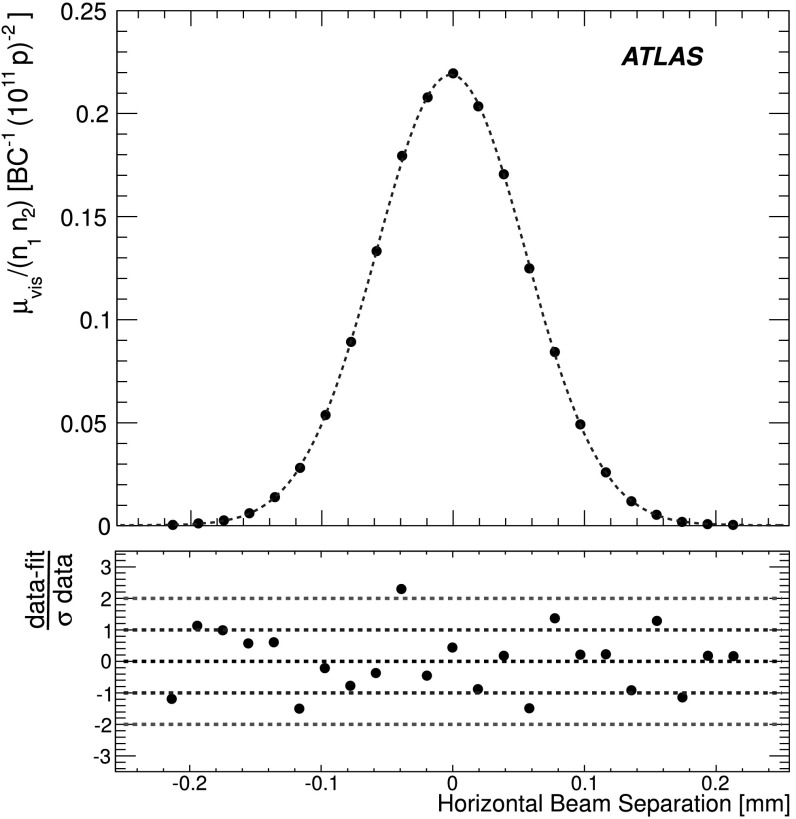
Specific visible interaction rate versus nominal beam separation for the BCMH_EventOR algorithm during scan VII in the horizontal plane for BCID 817. The residual deviation of the data from the Gaussian plus constant term fit, normalized at each point to the statistical uncertainty (*σ* data), is shown in the *bottom panel*

For each algorithm being calibrated, the *vdM* scan data are analysed in a very similar manner. For each BCID, the specific visible interaction rate *μ*_vis_/(*n*_1_ *n*_2_) is measured as a function of the “nominal” beam separation, i.e. the separation specified by the LHC control system for each scan step. The specific interaction rate is used so that the result is not affected by the change in beam currents over the duration of the scan. An example of the *vdM* scan data for a single BCID from scan VII in the horizontal plane is shown in Fig. 2.

The value of *μ*_vis_ is determined from the raw event rate using the analytic function described in Sect. 4.1 for the inclusive EventOR algorithms. The coincidence EventAND algorithms are more involved, and a numerical inversion is performed to determine *μ*_vis_ from the raw EventAND rate. Since the EventAND *μ* determination depends on $\sigma_{\mathrm {vis}}^{\mathrm{AND}}$ as well as $\sigma_{\mathrm{vis}}^{\mathrm{OR}}$, an iterative procedure must be employed. This procedure is found to converge after a few steps.

At each scan step, the beam separation and the visible interaction rate are corrected for beam–beam effects as described in Sect. 5.8. These corrected data for each BCID of each scan are then fitted independently to a characteristic function to provide a measurement of $\mu^{\mathrm{MAX}}_{\mathrm{vis}}$ from the peak of the fitted function, while *Σ* is computed from the integral of the function, using Eq. (13). Depending upon the beam conditions, this function can be a double Gaussian plus a constant term, a single Gaussian plus a constant term, a spline function, or other variations. As described in Sect. 6, the differences between the different treatments are taken into account as a systematic uncertainty in the calibration result.

**Fig. 3:**
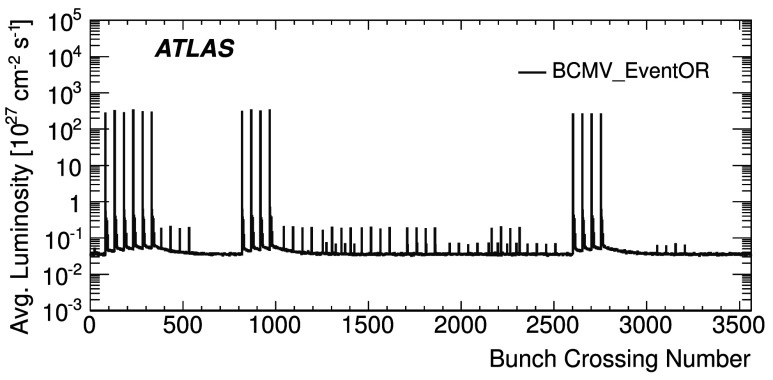
Average observed luminosity per BCID from BCMV_EventOR in the May 2011 *vdM* scan. In addition to the 14 large spikes in the BCIDs where two bunches are colliding, induced “afterglow” activity can also be seen in the following BCIDs. Single-beam background signals are also observed in BCIDs corresponding to unpaired bunches (24 in each beam)

One important difference in the *vdM* scan analysis between 2010 and 2011 is the treatment of the backgrounds in the luminosity signals. Figure 3 shows the average BCMV_EventOR luminosity as a function of BCID during the May 2011 *vdM* scan. The 14 large spikes around $\mathcal{L} \simeq3\times10^{29}\ \mathrm{cm}^{-2}\,\mathrm{s}^{-1}$ are the BCIDs containing colliding bunches. Both the LUCID and BCM detectors observe some small activity in the BCIDs immediately following a collision which tends to die away to some baseline value with several different time constants. This “afterglow” is most likely caused by photons from nuclear de-excitation, which in turn is induced by the hadronic cascades initiated by *pp* collision products. The level of the afterglow background is observed to be proportional to the luminosity in the colliding BCIDs, and in the *vdM* scans this background can be estimated by looking at the luminosity signal in the BCID immediately preceding a colliding bunch pair. A second background contribution comes from activity correlated with the passage of a single beam through the detector. This “single-beam” background, seen in Fig. 3 as the numerous small spikes at the 10^26^ cm^−2^ s^−1^ level, is likely a combination of beam-gas interactions and halo particles which intercept the luminosity detectors in time with the main beam. It is observed that this single-beam background is proportional to the bunch charge present in each bunch, and can be considerably different for beams 1 and 2, but is otherwise uniform for all bunches in a given beam. The single-beam background underlying a collision BCID can be estimated by measuring the single-beam backgrounds in unpaired bunches and correcting for the difference in bunch charge between the unpaired and colliding bunches. Adding the single-beam backgrounds measured for beams 1 and 2 then gives an estimate for the single-beam background present in a colliding BCID. Because the single-beam background does not depend on the luminosity, this background can dominate the observed luminosity response when the beams are separated.

In 2010, these background sources were accounted for by assuming that any constant term fitted to the observed scan curve is the result of luminosity-independent background sources, and has not been included as part of the luminosity integrated to extract *Σ*_*x*_ or *Σ*_*y*_. In 2011, a more detailed background subtraction is first performed to correct each BCID for afterglow and single-beam backgrounds, then any remaining constant term observed in the scan curve has been treated as a broad luminosity signal which contributes to the determination of *Σ*.

The combination of one *x* scan and one *y* scan is the minimum needed to perform a measurement of *σ*_vis_. The average value of $\mu^{\mathrm{MAX}}_{\mathrm{vis}}$ between the two scan planes is used in the determination of *σ*_vis_, and the correlation matrix from each fit between $\mu^{\mathrm{MAX}}_{\mathrm{vis}}$ and *Σ* is taken into account when evaluating the statistical uncertainty.

Each BCID should measure the same *σ*_vis_ value, and the average over all BCIDs is taken as the *σ*_vis_ measurement for that scan. Any variation in *σ*_vis_ between BCIDs, as well as between scans, reflects the reproducibility and stability of the calibration procedure during a single fill.

**Fig. 4:**
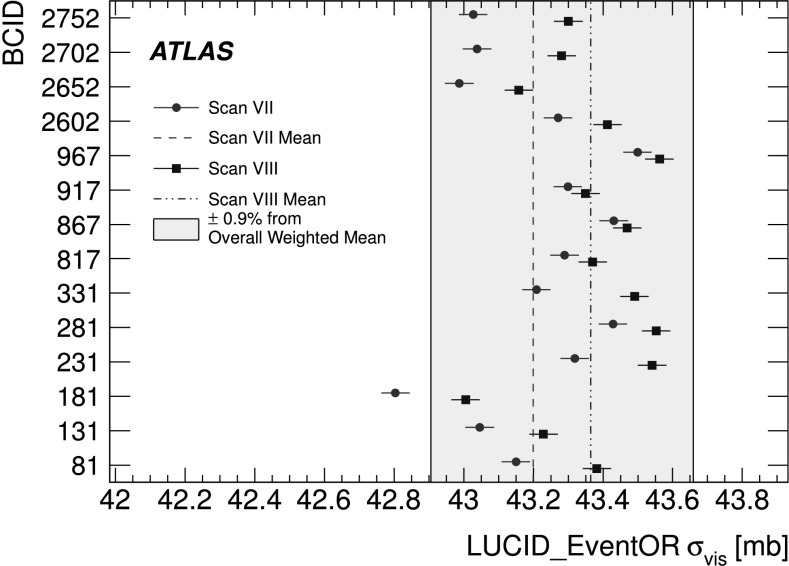
Measured *σ*_vis_ values for LUCID_EventOR by BCID for scans VII and VIII. The error bars represent statistical errors only. The *vertical lines* indicate the weighted average over BCIDs for scans VII and VIII separately. The *shaded band* indicates a ±0.9 % variation from the average, which is the systematic uncertainty evaluated from the per-BCID and per-scan *σ*_vis_ consistency

**Fig. 5:**
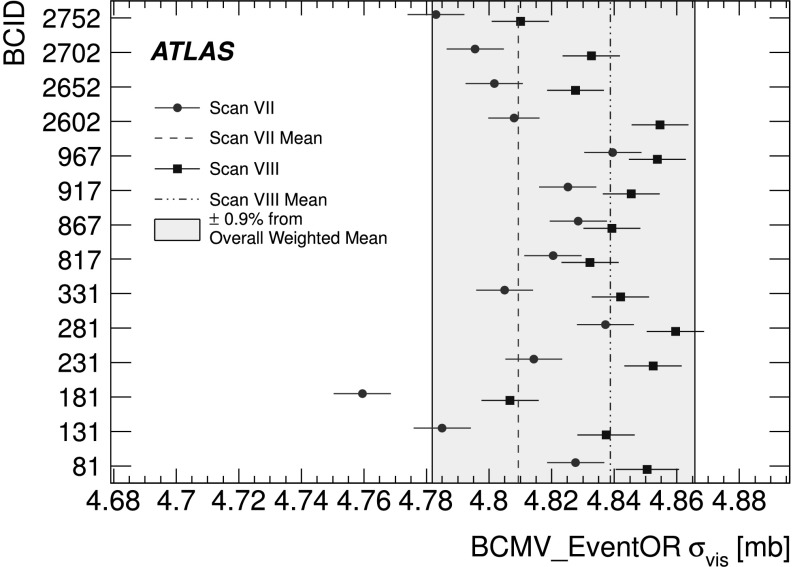
Measured *σ*_vis_ values for BCMV_EventOR by BCID for scans VII and VIII. The error bars represent statistical errors only. The *vertical lines* indicate the weighted average over BCIDs for Scans VII and VIII separately. The *shaded band* indicates a ±0.9 % variation from the average, which is the systematic uncertainty evaluated from the per-BCID and per-scan *σ*_vis_ consistency

Figure 4 shows the *σ*_vis_ values determined for LUCID_EventOR separately by BCID and by scan in the May 2011 scans. The RMS variation seen between the *σ*_vis_ results measured for different BCIDs is 0.4 % for scan VII and 0.3 % for scan VIII. The BCID-averaged *σ*_vis_ values found in scans VII and VIII agree to 0.5 % (or better) for all four LUCID algorithms. Similar data for the BCMV_EventOR algorithm are shown in Fig. 5. Again an RMS variation between BCIDs of up to 0.55 % is seen, and a difference between the two scans of up to 0.67 % is observed for the BCM_EventOR algorithms. The agreement in the BCM_EventAND algorithms is worse, with an RMS around 1 %, although these measurements also have significantly larger statistical errors.

Similar features are observed in the October 2010 scan, where the *σ*_vis_ results measured for different BCIDs, and the BCID-averaged *σ*_vis_ value found in scans IV and V agree to 0.3 % for LUCID_EventOR and 0.2 % for LUCID_EventAND. The BCMH_EventOR results agree between BCIDs and between the two scans at the 0.4 % level, while the BCMH_EventAND calibration results are consistent within the larger statistical errors present in this measurement.

### Internal scan consistency

The variation between the measured *σ*_vis_ values by BCID and between scans quantifies the stability and reproducibility of the calibration technique. Comparing Figs. 4 and 5 for the May 2011 scans, it is clear that some of the variation seen in *σ*_vis_ is not statistical in nature, but rather is correlated by BCID. As discussed in Sect. 6, the RMS variation of *σ*_vis_ between BCIDs within a given scan is taken as a systematic uncertainty in the calibration technique, as is the reproducibility of *σ*_vis_ between scans. The yellow band in these figures, which represents a range of ±0.9 %, shows the quadrature sum of these two systematic uncertainties. Similar results are found in the final scans taken in 2010, although with only 6 colliding bunch pairs there are fewer independent measurements to compare.

**Fig. 6:**
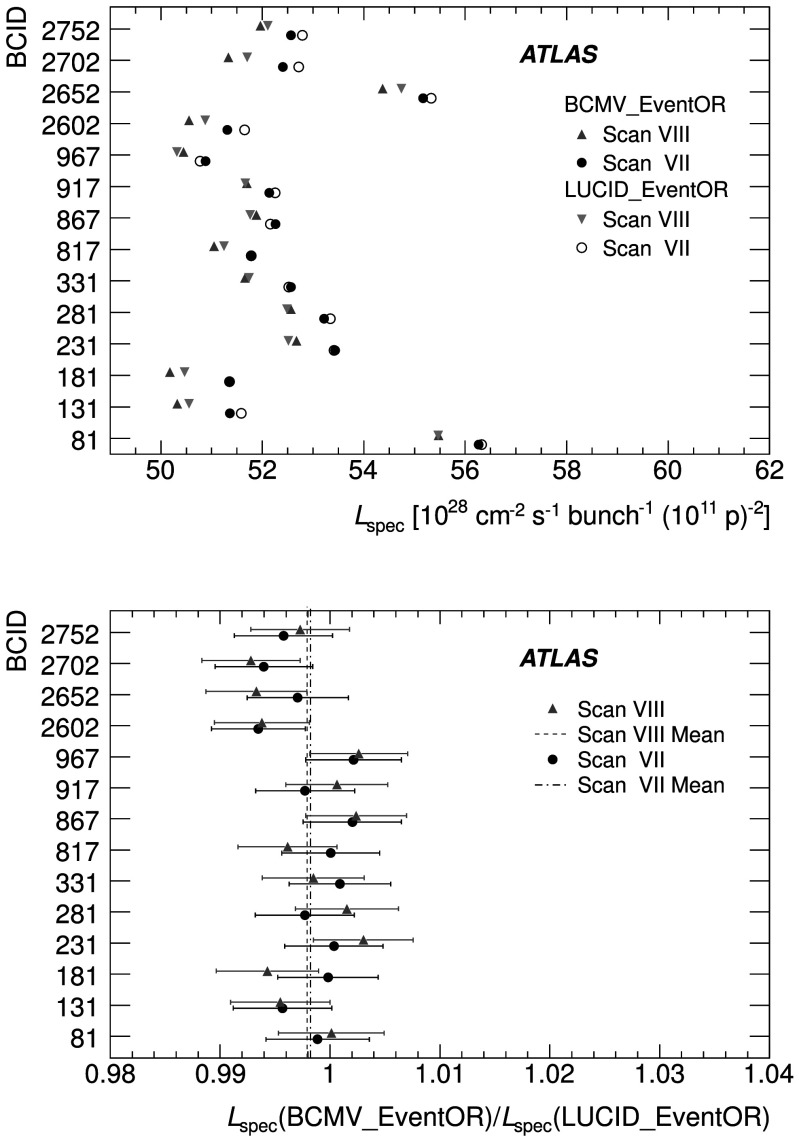
Specific luminosity determined by BCMV and LUCID per BCID for scans VII and VIII. The *figure on the top* shows the specific luminosity values determined by BCMV_EventOR and LUCID_EventOR, while the *figure on the bottom* shows the ratios of these values. The *vertical lines* indicate the weighted average over BCIDs for scans VII and VIII separately. The error bars represent statistical uncertainties only

Further checks can be made by considering the distribution of $\mathcal {L}_{\mathrm{\mathrm{spec}}}$ defined in Eq. (16) for a given BCID as measured by different algorithms. Since this quantity depends only on the convolved beam sizes, consistent results should be measured by all methods for a given scan. Figure 6 shows the measured $\mathcal{L}_{\mathrm{\mathrm {spec}}}$ values by BCID and scan for LUCID and BCMV algorithms, as well as the ratio of these values in the May 2011 scans. Bunch-to-bunch variations of the specific luminosity are typically 5–10 %, reflecting bunch-to-bunch differences in transverse emittance also seen during normal physics fills. For each BCID, however, all algorithms are statistically consistent. A small systematic reduction in $\mathcal{L}_{\mathrm{spec}}$ can be observed between scans VII and VIII, which is due to emittance growth in the colliding beams.

**Fig. 7:**
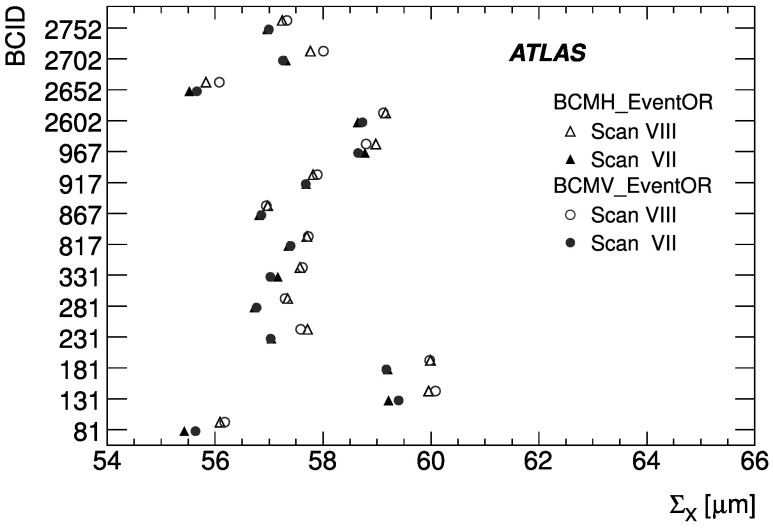
*Σ*_*x*_ determined by BCM_EventOR algorithms per BCID for scans VII and VIII. The statistical uncertainty on each measurement is approximately the size of the marker

**Fig. 8:**
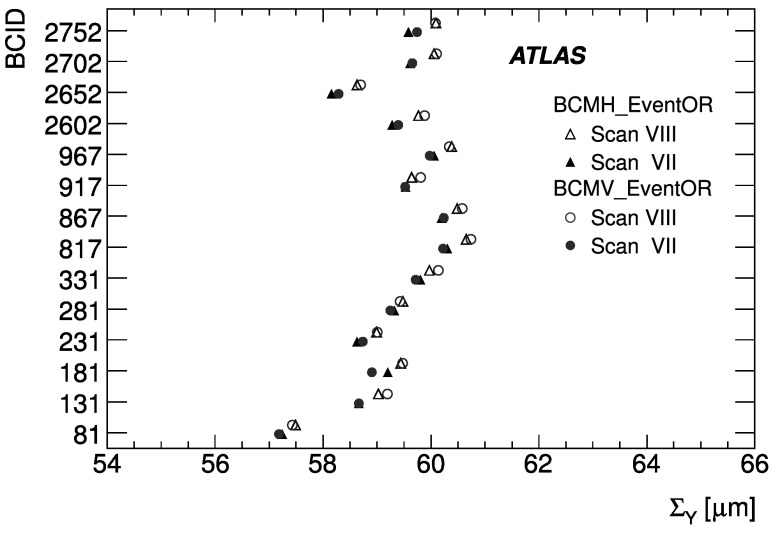
*Σ*_*y*_ determined by BCM_EventOR algorithms per BCID for scans VII and VIII. The statistical uncertainty on each measurement is approximately the size of the marker

**Fig. 9:**
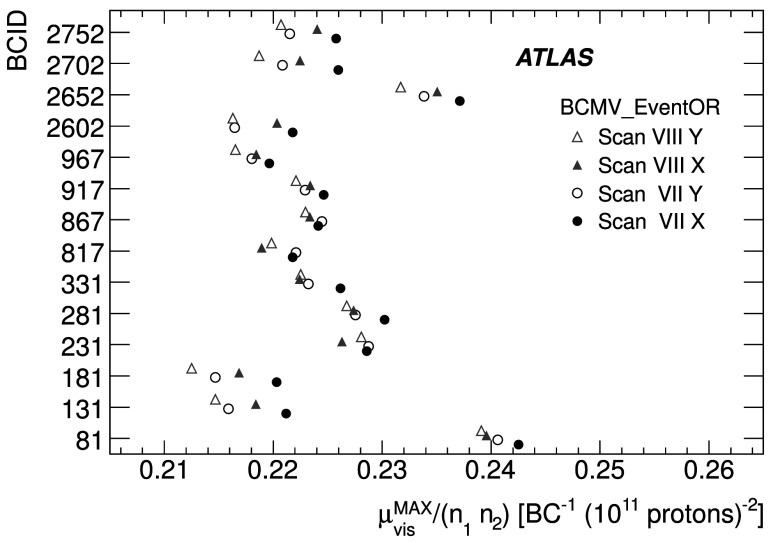
Peak specific interaction rate $\mu^{\mathrm{MAX}}_{\mathrm {vis}}/(n_{1} n_{2})$ determined by BCMV_EventOR per BCID for scans VII and VIII. The statistical uncertainty on each measurement is approximately the size of the marker

Figures 7 and 8 show the *Σ*_*x*_ and *Σ*_*y*_ values determined by the BCM algorithms during scans VII and VIII, and for each BCID a clear increase can be seen with time. This emittance growth can also be seen clearly as a reduction in the peak specific interaction rate $\mu_{\mathrm{vis}}^{\mathrm{MAX}}/(n_{1} n_{2})$ shown in Fig. 9 for BCMV_EventOR. Here the peak rate is shown for each of the four individual horizontal and vertical scans, and a monotonic decrease in rate is generally observed as each individual scan curve is recorded. The fact that the *σ*_vis_ values are consistent between scan VII and scan VIII demonstrates that to first order the emittance growth cancels out of the measured luminosity calibration factors. The residual uncertainty associated with emittance growth is discussed in Sect. 6.

### Bunch population determination

The dominant systematic uncertainty on the 2010 luminosity calibration, and a significant uncertainty on the 2011 calibration, is associated with the determination of the bunch population product (*n*_1_ *n*_2_) for each colliding BCID. Since the luminosity is calibrated on a bunch-by-bunch basis for the reasons described in Sect. 5.3, the bunch population per BCID is necessary to perform this calibration. Measuring the bunch population product separately for each BCID is also unavoidable as only a subset of the circulating bunches collide in ATLAS (14 out of 38 during the 2011 scan).

The bunch population measurement is performed by the LHC Bunch Current Normalization Working Group (BCNWG) and has been described in detail in Refs. [8, 9] for 2010 and Refs. [10–12] for 2011. A brief summary of the analysis is presented here, along with the uncertainties on the bunch population product. The relative uncertainty on the bunch population product (*n*_1_ *n*_2_) is shown in Table 3 for the *vdM* scan fills in 2010 and 2011.

**Table 3:** Systematic uncertainties on the determination of the bunch population product *n*_1_ *n*_2_ for the 2010 and 2011 *vdM* scan fills. The uncertainty on ghost charge and satellite bunches is included in the bunch-to-bunch fraction for scans I–V

Scan Number	I	II–III	IV–V	VII–VIII
LHC Fill Number	1059	1089	1386	1783
DCCT baseline offset	3.9 %	1.9 %	0.1 %	0.10 %
DCCT scale variation	2.7 %	2.7 %	2.7 %	0.21 %
Bunch-to-bunch fraction	2.9 %	2.9 %	1.6 %	0.20 %
Ghost charge and satellites	–	–	–	0.44 %
Total	5.6 %	4.4 %	3.1 %	0.54 %

The bunch currents in the LHC are determined by eight Bunch Current Transformers (BCTs) in a multi-step process due to the different capabilities of the available instrumentation. Each beam is monitored by two identical and redundant DC current transformers (DCCT) which are high-accuracy devices but do not have any ability to separate individual bunch populations. Each beam is also monitored by two fast beam-current transformers (FBCT) which have the ability to measure bunch currents individually for each of the 3564 nominal 25 ns slots in each beam. The relative fraction of the total current in each BCID can be determined from the FBCT system, but this relative measurement must be normalized to the overall current scale provided by the DCCT. Additional corrections are made for any out-of-time charge that may be present in a given BCID but not colliding at the interaction point.

The DCCT baseline offset is the dominant uncertainty on the bunch population product in early 2010. The DCCT is known to have baseline drifts for a variety of reasons including temperature effects, mechanical vibrations, and electromagnetic pick-up in cables. For each *vdM* scan fill the baseline readings for each beam (corresponding to zero current) must be determined by looking at periods with no beam immediately before and after each fill. Because the baseline offsets vary by at most ±0.8×10^9^ protons in each beam, the relative uncertainty from the baseline determination decreases as the total circulating currents go up. So while this is a significant uncertainty in scans I–III, for the remaining scans which were taken at higher beam currents, this uncertainty is negligible.

In addition to the baseline correction, the absolute scale of the DCCT must be understood. A precision current source with a relative accuracy of 0.1 % is used to calibrate the DCCT system at regular intervals, and the peak-to-peak variation of the measurements made in 2010 is used to set an uncertainty on the bunch current product of ±2.7 %. A considerably more detailed analysis has been performed on the 2011 DCCT data as described in Ref. [10]. In particular, a careful evaluation of various sources of systematic uncertainties and dedicated measurements to constrain these sources results in an uncertainty on the absolute DCCT scale in 2011 of 0.2 %.

Since the DCCT can measure only the total bunch population in each beam, the FBCT is used to determine the relative fraction of bunch population in each BCID, such that the bunch population product colliding in a particular BCID can be determined. To evaluate possible uncertainties in the bunch-to-bunch determination, checks are made by comparing the FBCT measurements to other systems which have sensitivity to the relative bunch population, including the ATLAS beam pick-up timing system. As described in Ref. [11], the agreement between the various determinations of the bunch population is used to determine an uncertainty on the relative bunch population fraction. This uncertainty is significantly smaller for 2011 because of a more sophisticated analysis, that exploits the consistency requirement that the visible cross-section be bunch-independent.

Additional corrections to the bunch-by-bunch fraction are made to correct for “ghost charge” and “satellite bunches”. Ghost charge refers to protons that are present in nominally empty BCIDs at a level below the FBCT threshold (and hence invisible), but still contribute to the current measured by the more precise DCCT. Satellite bunches describe out-of-time protons present in collision BCIDs that are measured by the FBCT, but that remain captured in an RF-bucket at least one period (2.5 ns) away from the nominally filled LHC bucket, and as such experience only long-range encounters with the nominally filled bunches in the other beam. These corrections, as well as the associated systematic uncertainties, are described in detail in Ref. [12].

### Length scale determination

Another key input to the *vdM* scan technique is the knowledge of the beam separation at each scan point. The ability to measure *Σ*_*x*/*y*_ depends upon knowing the absolute distance by which the beams are separated during the *vdM* scan, which is controlled by a set of closed orbit bumps^3^ applied locally near the ATLAS IP using steering correctors. To determine this beam-separation length scale, dedicated length scale calibration measurements are performed close in time to each *vdM* scan set using the same collision-optics configuration at the interaction point. Length scale scans are performed by displacing the beams in collision by five steps over a range of up to ±3*σ*_b_. Because the beams remain in collision during these scans, the actual position of the luminous region can be reconstructed with high accuracy using the primary vertex position reconstructed by the ATLAS tracking detectors. Since each of the four bump amplitudes (two beams in two transverse directions) depends on different magnet and lattice functions, the distance-scale calibration scans are performed so that each of these four calibration constants can be extracted independently. These scans have verified the nominal length scale assumed in the LHC control system at the ATLAS IP at the level of ±0.3 %.

### Beam–beam corrections

When charged-particle bunches collide, the electromagnetic field generated by a bunch in beam 1 distorts the individual particle trajectories in the corresponding bunch of beam 2 (and vice-versa). This so-called *beam–beam interaction* affects the scan data in two ways.

The first phenomenon, called *dynamic* *β* [13], arises from the mutual defocusing of the two colliding bunches: this effect is tantamount to inserting a small quadrupole at the collision point. The resulting fractional change in *β* ^∗^ (the value of the *β* function^4^ at the IP), or equivalently the optical demagnification between the LHC arcs and the collision point, varies with the transverse beam separation, sligthly modifying the collision rate at each scan step and thereby distorting the shape of the *vdM* scan curve.

Secondly, when the bunches are not exactly centred on each other in the *x*–*y* plane, their electromagnetic repulsion induces a mutual angular kick [15] that distorts the closed orbits by a fraction of a micrometer and modulates the actual transverse separation at the IP in a manner that depends on the separation itself. If left unaccounted for, these *beam–beam deflections* would bias the measurement of the overlap integrals in a manner that depends on the bunch parameters.

The amplitude and the beam-separation dependence of both effects depend similarly on the beam energy, the tunes^5^ and the unperturbed *β*-functions, as well as the bunch intensities and transverse beam sizes. The dynamic evolution of *β* ^∗^ during the scan is modelled using the MAD-X optics code [16] assuming bunch parameters representative of the May 2011 *vdM* scan (fill 1783), and then scaled using the measured intensities and convolved beam sizes of each colliding-bunch pair. The correction function is intrinsically independent of whether the bunches collide in ATLAS only, or also at other LHC interaction points [13]. The largest *β* ^∗^ variation during the 2011 scans is about 0.9 %.

The beam–beam deflections and associated orbit distortions are calculated analytically [17] assuming elliptical Gaussian beams that collide in ATLAS only. For a typical bunch, the peak angular kick during the 2011 scans is about ±0.5 μrad, and the corresponding peak increase in relative beam separation amounts to ±0.6 μm. The MAD-X simulation is used to validate this analytical calculation, and to verify that higher-order dynamical effects (such as the orbit shifts induced at other collision points by beam–beam deflections at the ATLAS IP) result in negligible corrections to the analytical prediction.

At each scan step, the measured visible interaction rate is rescaled by the ratio of the dynamic to the unperturbed bunch-size product, and the predicted change in beam separation is added to the nominal beam separation. Comparing the results of the scan analysis in Sect. 5.4 with and without beam–beam corrections for the 2011 scans, it is found that the visible cross-sections are increased by approximately 0.4 % from the dynamic-*β* correction and 1.0 % from the deflection correction. The two corrections combined amount to +1.4 % for 2011, and to +2.1 % for the October 2010 scans,^6^ reflecting the smaller emittances and slightly larger bunch intensities in that scan session.

### *vdM* scan results

The calibrated visible cross-section results for the *vdM* scans performed in 2010 and 2011 are shown in Tables 4 and 5. There were four algorithms which were calibrated in all five 2010 scans, while the BCMH algorithms were only available in the final two scans. The BCMV algorithms were not considered for luminosity measurements in 2010. Due to changes in the hardware or algorithm details between 2010 and 2011, the *σ*_vis_ values are not expected to be exactly the same in the two years.

**Table 4:** Visible cross-section measurements (in mb) determined from *vdM* scan data in 2011. Errors shown are statistical only

Scan Number	VII	VIII
Fill Number	1783	1783
LUCID_EventAND	13.660±0.003	13.726±0.003
LUCID_EventOR	43.20±0.01	43.36±0.01
LUCID_EventA	28.44±0.01	28.54±0.01
LUCID_EventC	28.48±0.01	28.60±0.01
BCMH_EventAND	0.1391±0.0004	0.1404±0.0004
BCMV_EventAND	0.1418±0.0004	0.1430±0.0004
BCMH_EventOR	4.762±0.002	4.792±0.003
BCMV_EventOR	4.809±0.003	4.839±0.003
Vertex (5 tracks)	39.00±0.02	39.12±0.02

**Table 5:** Visible cross-section measurements (in mb) determined from *vdM* scan data in 2010. Errors shown are statistical only

Scan Number	I	II	III	IV	V
Fill Number	1059	1089	1089	1386	1386
LUCID_EventAND	11.92±0.14	12.65±0.10	12.83±0.10	13.38±0.01	13.34±0.01
LUCID_EventOR	38.86±0.32	41.03±0.13	41.10±0.14	42.73±0.03	42.60±0.02
BCMH_EventAND				0.1346±0.0007	0.1341±0.0007
BCMH_EventOR				4.697±0.007	4.687±0.007
MBTS_Timing	48.3±0.3	50.2±0.2	49.9±0.2	52.4±0.2	52.3±0.2
PrimVtx	46.6±0.3	48.2±0.2	48.4±0.2	50.5±0.2	50.4±0.2

## Calibration uncertainties and results

This section outlines the systematic uncertainties which have been evaluated for the measurement of *σ*_vis_ from the *vdM* calibration scans for 2010 and 2011, and summarizes the calibration results. For scans I–III, the ability to make internal cross-checks is limited due to the presence of only one colliding bunch pair in these scans, and the systematic uncertainties for these scans are unchanged from those evaluated in Ref. [18]. Starting with scans IV and V, the redundancy from having multiple bunch pairs colliding has allowed a much more detailed study of systematic uncertainties.

The five different scans taken in 2010 have different systematic uncertainties, and the combination process used to determine a single *σ*_vis_ value is described in Sect. 6.2. For 2011, the two *vdM* scans are of equivalent quality, and the calibration results are simply averaged based on the statistical uncertainties. Tables 6 and 7 summarize the systematic uncertainties on the calibration in 2010 and 2011 respectively, while the combined calibration results are shown in Table 8.

**Table 6:** Relative systematic uncertainties on the determination of the visible cross-section *σ*_vis_ from *vdM* scans in 2010. The assumed correlations of these parameters between scans is also indicated

Scan Number	I	II–III	IV–V	
Fill Number	1059	1089	1386	
Beam centring	2 %	2 %	0.04 %	Uncorrelated
Beam-position jitter	–	–	0.3 %	Uncorrelated
Emittance growth and other non-reproducibility	3 %	3 %	0.5 %	Uncorrelated
Fit model	1 %	1 %	0.2 %	Partially Correlated
Length scale calibration	2 %	2 %	0.3 %	Partially Correlated
Absolute length scale	0.3 %	0.3 %	0.3 %	Correlated
Beam–beam effects	–	–	0.7 %	Uncorrelated
Transverse correlations	3 %	2 %	0.9 %	Partially Correlated
*μ* dependence	2 %	2 %	0.5 %	Correlated
Scan subtotal	5.6 %	5.1 %	1.5 %	
Bunch population product	5.6 %	4.4 %	3.1 %	Partially Correlated
Total	7.8 %	6.8 %	3.4 %

**Table 7:** Relative systematic uncertainties on the determination of the visible cross-section *σ*_vis_ from *vdM* scans in 2011

Scan Number	VI–VII
Fill Number	1783
Beam centring	0.10 %
Beam-position jitter	0.30 %
Emittance growth and other non-reproducibility	0.67 %
Bunch-to-bunch *σ*_vis_ consistency	0.55 %
Fit model	0.28 %
Background subtraction	0.31 %
Specific Luminosity	0.29 %
Length scale calibration	0.30 %
Absolute length scale	0.30 %
Beam–beam effects	0.50 %
Transverse correlations	0.50 %
*μ* dependence	0.50 %
Scan subtotal	1.43 %
Bunch population product	0.54 %
Total	1.53 %

**Table 8:** Best estimates of the visible cross-section determined from *vdM* scan data for 2010 and 2011. Total uncertainties are shown including the statistical component and the total systematic uncertainty taking all correlations into account. The 2010 and 2011 values are not expected to be consistent due to changes in the hardware for LUCID and BCM, and changes in the algorithm used for vertex counting

	Visible cross-section $\overline{\sigma}_{\mathrm{vis}}$ (mb)	
	2010	2011
LUCID_EventAND	13.3±0.5	13.7±0.2
LUCID_EventOR	42.5±1.5	43.3±0.7
LUCID_EventA		28.5±0.4
LUCID_EventC		28.5±0.4
BCMH_EventAND	0.134±0.005	0.140±0.002
BCMV_EventAND		0.142±0.002
BCMH_EventOR	4.69±0.16	4.78±0.07
BCMV_EventOR		4.82±0.07
MBTS_Timing	52.1±1.8	
PrimVtx	50.2±1.7	
Vertex (5 tracks)		39.1±0.6

### Calibration uncertainties

#### Beam centring

If the beams are not perfectly centred in the non-scanning plane at the start of a *vdM* scan, the assumption that the luminosity observed at the peak is equal to the maximum head-on luminosity is not correct. In the last set of 2010 scans and the 2011 scans, the beams were centred at the beginning of the scan session, and the maximum observed non-reproducibility in relative beam position at the peak of the fitted scan curve is used to determine the uncertainty. For instance, in the 2011 scan the maximum offset is 3 μm, corresponding to a 0.1 % error on the peak instantaneous interaction rate.

#### Beam-position jitter

At each step of a scan, the actual beam separation may be affected by random deviations of the beam positions from their nominal setting. The magnitude of this potential “jitter” has been evaluated from the shifts in relative beam centring recorded during the length-scale calibration scans described in Sect. 5.7, and amounts to aproximately 0.6 μm RMS. Very similar values are observed in 2010 and 2011. The resulting systematic uncertainty on *σ*_vis_ is obtained by randomly displacing each measurement point by this amount in a series of simulated scans, and taking the RMS of the resulting variations in fitted visible cross-section. This procedure yields a ±0.3 % systematic error associated with beam-positioning jitter during scans IV–VIII. For scans I–III, this is assumed to be part of the 3 % non-reproducibility uncertainty.

#### Emittance growth

The *vdM* scan formalism assumes that the luminosity and the convolved beam sizes *Σ*_*x*/*y*_ are constant, or more precisely that the transverse emittances of the two beams do not vary significantly either in the interval between the horizontal and the associated vertical scan, or within a single *x* or *y* scan.

Emittance growth between scans would manifest itself by a slight increase of the measured value of *Σ* from one scan to the next. At the same time, emittance growth would decrease the peak specific luminosity in successive scans (i.e. reduce the specific visible interaction rate at zero beam separation). Both effects are clearly visible in the 2011 May scan data presented in Sect. 5.5, where Figs. 7 and 8 show the increase in *Σ* and Fig. 9 shows the reduction in the peak interaction rate.

In principle, when computing the visible cross-section using Eq. (15), the increase in *Σ* from scan to scan should exactly cancel the decrease in specific interaction rate. In practice, the cancellation is almost complete: the bunch-averaged visible cross-sections measured in scans IV–V differ by at most 0.5 %, while in scans VII–VIII the values differ by at most 0.67 %. These maximum differences are taken as estimates of the systematic uncertainties due to emittance growth.

Emittance growth within a scan would manifest itself by a very slight distortion of the scan curve. The associated systematic uncertainty determined by a toy Monte Carlo study with the observed level of emittance growth was found to be negligible.

For scans I–III, an uncertainty of 3 % was determined from the variation in the peak specific interaction rate between successive scans. This uncertainty is assumed to cover both emittance growth and other unidentified sources of non-reproducibility. Variations of such magnitude were not observed in later scans.

#### Consistency of bunch-by-bunch visible cross-sections

The calibrated *σ*_vis_ value found for a given detector and algorithm should be a constant factor independent of machine conditions or BCID. Comparing the *σ*_vis_ values determined by BCID in Figs. 4 and 5, however, it is clear that there is some degree of correlation between these values: the scatter observed is not entirely statistical in nature. The RMS variation of *σ*_vis_ for each of the LUCID and BCM algorithms is consistently around 0.5 %, except for the BCM_EventAND algorithms, which have much larger statistical uncertainties. An additional uncertainty of ±0.55 % has been applied, corresponding to the largest RMS variation observed in either the LUCID or BCM measurements to account for this observed BCID dependence in 2011. For the 2010 scans, only scans IV–V have multiple BCIDs with collisions, and in those scans the agreement between BCIDs and between scan sessions was consistent with the statistical accuracy of the comparison. As such, no additional uncertainty beyond the 0.5 % derived for emittance growth was assigned.

#### Fit model

The *vdM* scan data in 2010 are analysed using a fit to a double Gaussian plus a constant background term, while for 2011 the data are first corrected for known backgrounds, then fitted to a single Gaussian plus constant term. Refitting the data with several different model assumptions including a cubic spline function and no constant term leads to different values of *σ*_vis_. The maximum variation between these different fit assumptions is used to set an uncertainty on the fit model.

#### Background subtraction

The importance of the background subtraction used in the 2011 *vdM* analysis is evaluated by comparing the visible cross-section measured by the BCM_EventOR algorithms when the detailed background subtraction is performed or not performed before fitting the scan curve. Half the difference (0.31 %) is adopted as a systematic uncertainty on this procedure. For scans IV–V, no dedicated background subtraction was performed and the uncertainty on the background treatment is accounted for in the fit model uncertainty, where one of the comparisons is between assuming the constant term results from luminosity-independent background sources compared to a luminosity-dependent signal.

#### Reference specific luminosity

The transverse convolved beam sizes *Σ*_*x*/*y*_ measured by the *vdM* scan are directly related to the specific luminosity defined in Eq. (16). Since this specific luminosity is determined by the beam parameters, each detector and algorithm should measure identical values from the scan curve fits.

For simplicity, the visible cross-section value extracted from a set of *vdM* scans for a given detector and algorithm uses the convolved beam sizes measured by that same detector and algorithm.^7^ As shown in Fig. 6, the values measured by LUCID_EventOR and BCM_EventOR are rather consistent within statistical uncertainties, although averaged over all BCIDs there may be a slight systematic difference between the two results. The difference observed between these two algorithms, after averaging over all BCIDs, results in a systematic uncertainty of 0.29 % related to the choice of specific luminosity value.

#### Length-scale calibration

The length scale of each scan step enters into the extraction of *Σ*_*x*/*y*_ and hence directly affects the predicted peak luminosity during a *vdM* scan. The length scale calibration procedure is described in Sect. 5.7 and results in a ±0.3 % uncertainty for scans IV–VIII. For scans I–III, a less sophisticated length scale calibration procedure was performed which was more sensitive to hysteresis effects and re-centring errors resulting in a correspondingly larger systematic uncertainty of 2 %.

#### Absolute length scale of the Inner Detector

The determination of the length scale relies on comparing the scan step requested by the LHC with the actual transverse displacement of the luminous centroid measured by ATLAS. This measurement relies on the length scale of the Inner Detector tracking system (primarily the pixel detector) being correct in measuring displacements of vertex positions away from the centre of the detector. An uncertainty on this absolute length scale was evaluated by analysing Monte Carlo events simulated using several different misaligned Inner Detector geometries. These geometries represent distortions of the pixel detector which are at the extreme limits of those allowed by the data-driven alignment procedure. Samples were produced with displaced interaction points to simulate the transverse beam displacements seen in a *vdM* scan. The variations between the true and reconstructed vertex positions in these samples give a conservative upper bound of ±0.3 % on the uncertainty on the determination of *σ*_vis_ due to the absolute length scale.

#### Beam–beam effects

For given values of the bunch intensity and transverse convolved beam sizes, which are precisely measured, the deflection-induced orbit distortion and the relative variation of *β* ^∗^ are both proportional to *β* ^∗^ itself; they also depend on the fractional tune. Assigning a ±20 % uncertainty on each *β*-function value at the IP and a ±0.02 upper limit on each tune variation results in a ±0.5 % (±0.7 %) uncertainty on *σ*_vis_ for 2011 (2010). This uncertainty is computed under the conservative assumption that *β*-function and tune uncertainties are correlated between the horizontal and vertical planes, but uncorrelated between the two LHC rings; it also includes a contribution that accounts for small differences between the analytical and simulated beam–beam-induced orbit distortions.

#### Transverse correlations

The *vdM* formalism outlined in Sect. 5.1 explicitly assumes that the particle densities in each bunch can be factorized into independent horizontal and vertical components such that the term 1/(2*πΣ*_*x*_ *Σ*_*y*_) in Eq. (14) fully describes the overlap integral of the two beams. If the factorization assumption is violated, the convolved beam width *Σ* in one plane is no longer independent of the beam separation *δ* in the other plane, although a straightforward generalization of the *vdM* formalism still correctly handles an arbitrary two-dimensional luminosity distribution as a function of the transverse beam separation (*δ*_*x*_,*δ*_*y*_), provided this distribution is known with sufficient accuracy.

Linear *x*–*y* correlations do not invalidate the factorization assumption, but they can rotate the ellipse which describes the luminosity distribution away from the *x*–*y* scanning planes such that the measured *Σ*_*x*_ and *Σ*_*y*_ values no longer accurately reflect the true convolved beam widths [19]. The observed transverse displacements of the luminous region during the scans from reconstructed event vertex data directly measure this effect, and a 0.1 % upper limit on the associated systematic uncertainty is determined. This uncertainty is comparable to the upper limit on the rotation of the luminous region derived during 2010 LHC operations from measurements of the LHC lattice functions by resonant excitation, combined with emittance ratios based on wire-scanner data [20].

More general, non-linear correlations violate the factorization assumption, and additional data are used to constrain any possible bias in the luminosity calibration from this effect. These data include the event vertex distributions, where both the position and shape of the three-dimensional luminous region are measured for each scan step, and the offset scan data from scan IX, where the convolved beam widths are measured with a fixed beam–beam offset of 160 μm in the non-scanning plane. Two different analyses are performed to determine a systematic uncertainty.

First, a simulation of the collision process, starting with single-beam profiles constructed from the sum of two three-dimensional Gaussian distributions with arbitrary widths and orientations, is performed by numerically evaluating the overlap integral of the bunches. This simulation, which allows for a crossing angle in both planes, is performed for each scan step to predict the geometry of the luminous region, along with the produced luminosity. Since the position and shape of the luminous region during a beam-separation scan varies depending on the single-beam parameters [21], the simulation parameters are adjusted to provide a reasonable description of the mean and RMS width of the luminous region observed at each scan step in the May 2011 scans VII–IX (including the offset scan). Luminosity profiles are then generated for simulated *vdM* scans using these tuned beam parameters, and analysed in the same fashion as the real *vdM* scan data, which assumes factorization. The impact of a small non-factorization in the single-beam distributions is determined from the difference between the ‘true’ luminosity from the simulated overlap integral at zero beam separation and the ‘measured’ luminosity from the luminosity profile fits. This difference is 0.1–0.2 % for the May 2011 scans, depending on the fitting model used. The number of events with vertex data recorded during the 2010 *vdM* scans is not sufficient to perform a similar analysis for those scans.

A second approach, which does not use the luminous region data, fits the observed luminosity distributions as a function of beam separation to a number of generalized, two-dimensional functions. These functions include non-factorizable functions constructed from multiple two-dimensional Gaussian distributions with possible rotations from the scan axes, and other functions where factorization between the scan axes is explicitly imposed. By performing a combined fit to the luminosity data in the two scan planes of scan VII, plus the two scan planes in the offset scan IX, the relative difference between the non-factorizable and factorizable functions is evaluated for 2011. The resulting fractional difference on *σ*_vis_ is 0.5 %. For 2010, no offset scan data are available, but a similar analysis performed on scans IV and V found a difference of 0.9 %.

The systematic uncertainty associated with transverse correlations is taken as the largest effect among the two approaches described above, to give an uncertainty of 0.5 % for 2011. For 2010, the 0.9 % uncertainty is taken as the difference between non-factorizable and factorizable fit models.

#### *μ* dependence

**Fig. 10:**
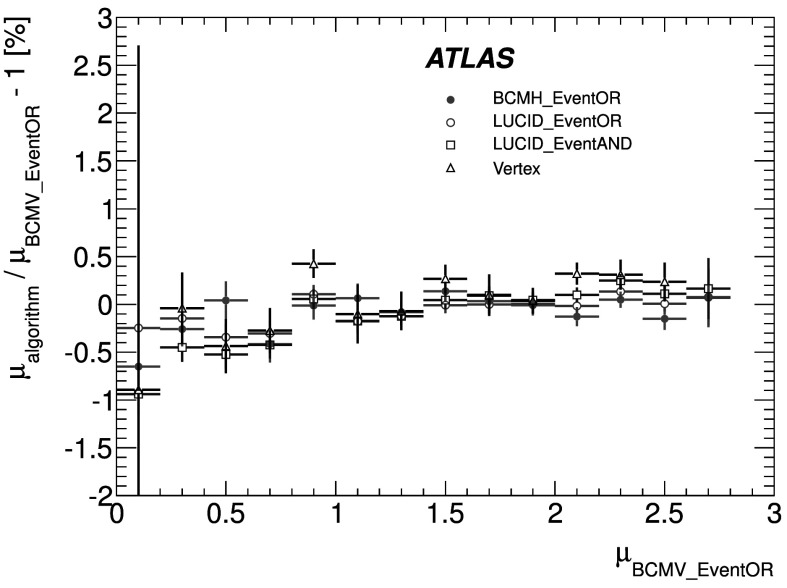
Fractional deviation in the average value of *μ* obtained using different algorithms with respect to the BCMV_EventOR value as a function of *μ* during scans VII–VIII

Scans IV–V were taken over a range of interactions per bunch crossing 0<*μ*<1.3 while scans VII–VIII covered the range 0<*μ*<2.6, so uncertainties on the *μ* correction can directly affect the evaluation of *σ*_vis_. Figure 10 shows the variation in measured luminosity as a function of *μ* between several algorithms and detectors in 2011, and on the basis of this agreement an uncertainty of ±0.5 % has been applied for scans IV–VIII.^8^

Scans I–III were performed with *μ*≪1 and so uncertainties in the treatment of the *μ*-dependent corrections are small. A ±2 % uncertainty was assigned, however, on the basis of the agreement at low *μ* values between various detectors and algorithms, which were described in Ref. [2].

#### Bunch-population product

The determination of this uncertainty has been described in Sect. 5.6 and the contributions are summarized in Table 3.

### Combination of 2010 scans

The five *vdM* scans in 2010 were taken under very different conditions and have very different systematic uncertainties. To combine the individual measurements of *σ*_vis_ from the five scans to determine the best calibrated $\overline{\sigma}_{\mathrm{vis}}$ value per algorithm, a Best Linear Unbiased Estimator (BLUE) technique has been employed taking into account both statistical and systematic uncertainties, and the appropriate correlations [23, 24]. The BLUE technique is a generalization of a *χ* ^2^ minimization, where for any set of measurements *x*_*i*_ of a physical observable *θ*, the best estimate of *θ* can be found by minimizing

17 $$ \chi^2 = (\mathbf{x} - \boldsymbol{\theta})^\mathrm{T} \mathbf{V}^{-1} (\mathbf {x} - \boldsymbol{\theta}) $$

where **V** ^−1^ is the inverse of the covariance matrix **V**, and ***θ*** is the product of the unit vector and *θ*.

Using the systematic uncertainties described above, including the correlations indicated in Table 6, a covariance matrix is constructed for each error source according to *V*_*ij*_=*σ*_*i*_ *σ*_*j*_ *ρ*_*ij*_ where *σ*_*i*_ is the uncertainty from a given source for scan *i*, and *ρ*_*ij*_ is the linear correlation coefficient for that error source between scans *i* and *j*. As there are a total of five *vdM* scans, a 5×5 covariance matrix is determined for each source of uncertainty. These individual covariance matrices are combined to produce the complete covariance matrix, along with the statistical uncertainty shown in Table 5. While in principle, each algorithm and detector indicated in Table 5 could have different systematic uncertainties, no significant sources of systematic uncertainty have been identified which vary between algorithms. As a result, a common systematic covariance matrix has been used in all combinations.

The best estimate of the visible cross-section $\overline{\sigma}_{\mathrm{vis}}$ for each luminosity method in 2010 is shown in Table 8 along with the uncertainty. Because the same covariance matrix is used in all combinations aside from the small statistical component, the relative weighting of the five scan points is almost identical for all methods. Here detailed results are given for the LUCID_EventOR combination. Because most of the uncorrelated uncertainties were significantly reduced from scans I–III to scans IV–V, the values from the last two *vdM* scans dominate the combination. Scans IV and V contribute a weight of 45 % each, while the other three scans make up the remaining 10 % of the weighted average value. The total uncertainty on the LUCID_EventOR combination represents a relative error of ±3.4 %, and is nearly identical to the uncertainty quoted for scans IV–V alone in Table 6. Applying the beam–beam corrections described in Sect. 5.8, which only affect scans IV–V in 2010, changes the best estimate of $\overline{\sigma}_{\mathrm{vis}}$ by +1.9 % compared to making no corrections to the 2010 calibrations.

**Fig. 11:**
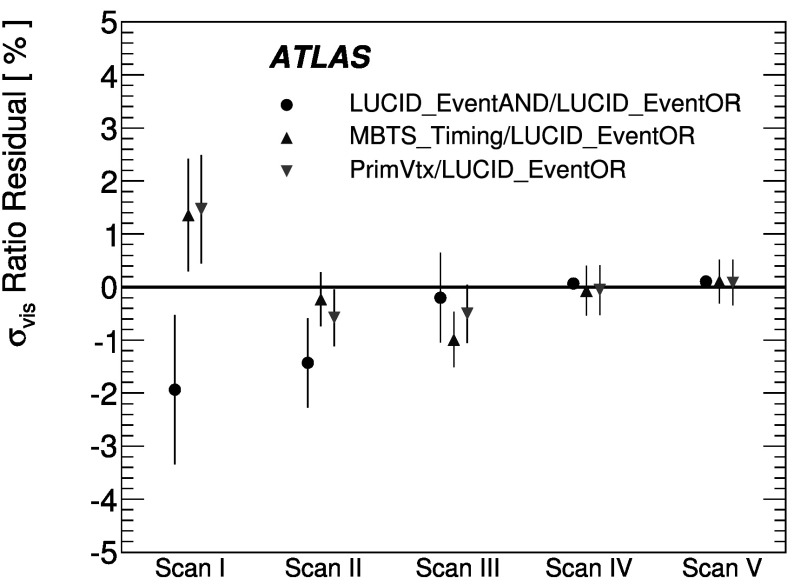
Residuals of the *σ*_vis_ ratios between algorithms for each scan in 2010 are shown as a relative deviation from the mean ratio based on $\overline{\sigma}_{\mathrm{vis}}$. Error bars represent statistical uncertainties only

Figure 11 shows the agreement among the algorithms within each scan in 2010 by plotting the deviations of the ratios $\sigma_{\mathrm{vis}}/\sigma_{\mathrm{vis}}(\mathrm{LUCID\_EventOR})$ for several algorithms from the mean value of these ratios, $\overline{\sigma }_{\mathrm{vis}}/\overline{\sigma}_{\mathrm{vis}}$(LUCID_EventOR). By construction, any variation between scans related to the bunch population product *n*_1_ *n*_2_ cancels out, and the remaining scatter reflects the variation between algorithms in measuring $\mu_{\mathrm{vis}}^{\mathrm{MAX}} \varSigma _{x} \varSigma_{y}$. The observed variation is mostly consistent with the statistical uncertainties, and the observed variation of up to ±2 % is consistent with the systematic uncertainty assigned to scans I–III for *μ* dependence. No evidence for any additional source of significant systematic uncertainty between the algorithms is apparent.

## Luminosity extrapolation

The $\overline{\sigma}_{\mathrm{vis}}$ values determined in Sect. 6 allow each calibrated algorithm to provide luminosity measurements over the course of the 2010 and 2011 runs. Several additional effects due to the LHC operating with a large number of bunches and large *μ* values must be considered for the 2011 data, however, and additional uncertainties related to the extrapolation of the *vdM* scan calibration to the complete data sample must be evaluated.

Several specific corrections are described in this section for the 2011 data, while more general uncertainties, related to the agreement and stability of the various luminosity methods applicable to both 2010 and 2011, are described in Sect. 8.

### 2011 hardware changes

Several changes were made to the readout chain of both the BCM and LUCID detectors before and during the early 2011 data-taking period.

During the 2010–2011 LHC winter shutdown, resistors on the BCM front-end boards were replaced to increase the dynamic range of the low-gain BCM signals used for beam-abort monitoring. While the adjustments were performed in a way that should have left the high-gain BCM signal (used for the luminosity measurement) unchanged, variations at the percent level remain possible. As a result, the BCM calibration in 2010 is not expected to be directly applicable to the 2011 data.

On 21 April 2011, the BCM thresholds were adjusted to place them at a better point in the detector response plateau. As this change was made during a period with stable beams, the ratio of the BCM luminosity to that of any other detector shows a clear step, which can be used to measure directly the relative change in *σ*_vis_ due to this adjustment. After the threshold change, the luminosity measured by BCMH_EventOR was observed to increase with respect to other detectors by +3.1 %, which implies that the *σ*_vis_ value for BCMH_EventOR decreased by this amount. For BCMV the equivalent luminosity change is +4.1 %. Since the 2011 *vdM* scan calibration happened after this date, for any BCM data taken before this threshold change, the *σ*_vis_ values applied have been scaled up accordingly from the 2011 calibrated values. The total change in *σ*_vis_ for BCMH_EventOR shown in Table 8 is +2.5 %, implying that over the 2010–2011 winter shutdown the BCMH_EventOR response changed by about +5.6 %.

During the LHC technical stop in early April 2011, the LUCID receiver cards were changed to improve the performance of the readout with 50 ns bunch spacing. Since this change was made during a period with no collisions, there is no direct measurement of the shift in LUCID calibration. Using data taken before and after the technical stop it can be estimated that the LUCID_EventOR *σ*_vis_ value increased by about 2–3 %. The total change in LUCID_EventOR calibration from 2010 to 2011 shown in Table 8 is +2.4 %, which indicates that the LUCID *σ*_vis_ calibration is consistent between 2010 and 2011 at a level of approximately 1 %.

Finally, on 30 July 2011, the radiator gas was removed from the LUCID Cherenkov tubes and the detector was operated for the rest of the 2011 physics run using only the Cherenkov signal from the quartz window. This reduction in detector efficiency was motivated by several factors, including the increasing interaction rate which was starting to saturate the LUCID_EventOR response when the detector was filled with gas, as well as the better stability and linearity observed without gas. The calibration of the LUCID luminosity measurements without gas was determined by comparing to the TileCal luminosity as described in Sect. 7.3.

### Backgrounds

As described in Sect. 5.4, both the LUCID and BCM detectors observe some small “afterglow” activity in the BCIDs immediately following a collision in normal physics operations. With a 2011 bunch spacing of 50 ns and a relatively large number of bunches injected into the LHC, this afterglow tends to reach a fairly stable equilibrium after the first few bunches in a train, and is observed to scale with the instantaneous luminosity.

**Fig. 12:**
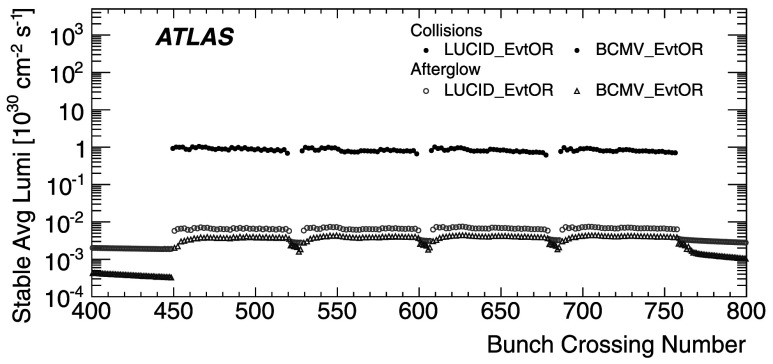
Observed luminosity averaged over the fill as a function of BCID for the LUCID_EventOR and BCMV_EventOR algorithms for a single LHC fill with 1042 colliding bunch pairs. On this scale the BCMV and LUCID luminosity values for colliding BCIDs are indistinguishable. The small “afterglow” luminosity comes in BCIDs where no bunches are colliding and is the result of induced activity seen in the detectors. Only 400 BCIDs are shown so that the details of the afterglow in the short and long gaps in the fill pattern can be seen more clearly

Figure 12 shows the luminosity as determined by LUCID_EventOR and BCMV_EventOR for a span of 400 BCIDs within a fill in June 2011 with 1042 colliding bunch pairs. The afterglow level can be seen to be roughly constant at the 1 % level for LUCID_EventOR and at the 0.5 % level for BCMV_EventOR during the bunch train, and dropping during gaps in the fill pattern.

To assess the effect of afterglow, the probability of an afterglow event must be combined with the Poisson probabilities outlined in Sect. 4.1 to obtain the correction to the observed *μ* value. For EventOR and HitOR algorithms, this correction is *μ*=*μ*_obs_−*μ*_bgd_ while for the EventAND algorithms a considerably more involved formula must be applied. To estimate *μ*_bgd_, the calibrated *μ* value observed in the BCID immediately preceding a collision has been used. Different estimates using the following BCID or the average of the preceding and following BCIDs produce negligibly different results.

This afterglow subtraction has been applied to all BCM and LUCID luminosity determinations. Since the afterglow level in the BCID immediately following a colliding bunch may be different from the level in the second BCID after a colliding bunch, BCIDs at the end of a bunch train have been used to evaluate any possible bias in the afterglow correction. It is observed that the simple afterglow subtraction over-corrects for the afterglow background in the BCMH_EventOR algorithm by approximately 0.2 %, although for the BCMV_EventOR algorithm the method works better. A systematic uncertainty of ±0.2 % is assigned to cover any possible bias on the BCMV_EventOR luminosity. The LUCID_EventOR algorithm is over-corrected by around 0.5 %, and this bias is removed by applying a constant scale factor to the LUCID luminosity measurements. A more detailed comparison, using luminosity data from a single-bunch run to construct an afterglow “template” which can be combined with any arbitrary bunch pattern to emulate the behavior in a train, yields consistent results.

Afterglow in 2010 was considerably less important due to the 150 ns bunch spacing, and the relatively short trains used that year. Afterglow is generally negligible in *vdM* scans due to the small number of colliding bunches and the large spacing between them.

The additional single-beam backgrounds observed by both BCM and LUCID are generally negligible during normal physics operations as these luminosity-independent backgrounds are tiny compared to the typical signal during physics operations. These backgrounds must be treated carefully, however, during *vdM* scans or other special beam tests which involve low-luminosity running.

### LUCID PMT current correction

Due to the increase in the total luminosity delivered by the LHC, both in terms of the number of bunches colliding and of the average number of interactions per bunch crossing, the LUCID PMTs in 2011 were operating in a regime where the average anodic PMT current is of order 10 μA, which has an observable effect on the PMT gain.

Uncorrected, this effect shows up both as an apparent *μ* dependence of the luminosity, since the PMT currents are highly correlated with the average *μ* during a fill, as well as a long-term time dependence in the LUCID luminosity value, since the number of colliding bunches steadily increased in 2011. The magnitude of this effect was of the order of 4 % on the LUCID_EventOR luminosity by the end of 2011.

The total anodic current summed over all LUCID tubes has been observed to produce a deviation of the luminosity measured by the various LUCID algorithms with respect to the TileCal value. A correction for this effect has been evaluated using a single ATLAS run with 1317 colliding bunches. TileCal is used as the reference, and a second-order polynomial is fitted to the ratio between the LUCID and TileCal luminosity, for all the algorithms, as a function of the total anodic PMT current. This PMT current correction has been applied to all LUCID data used to describe luminosity during physics operations.

The constant term of the fitted function, representing the extrapolation to zero PMT anodic current, provides the correction to be applied to the LUCID *vdM* calibration resulting from the removal of the radiator gas from the detector, as well as from any ageing-related variation in PMT gain to that point in time. As discussed in Sect. 4.4, the TileCal luminosity calibration is performed relative to LUCID_EventOR at the time of the *vdM* scan. As a result, the LUCID and TileCal luminosity measurements are implicitly tied to each other at one point in time, although any long-term variations away from that point are still significant. Similarly, any *μ* dependence between the LUCID and TileCal response is largely removed by this correction procedure, although comparisons to other detectors remain relevant.

### BCM calibration shifts

The BCM detectors are solid-state devices constructed from chemical vapour deposition diamonds to provide tolerance to high radiation levels. A well-known feature of such detectors is a tendency for the gain to increase under moderate irradiation levels up to a stable asymptotic value at high dose rates [25, 26]. This so-called “pumping” is generally ascribed to the filling of charge traps in the diamond sensors with continued irradiation until enough charge has been sent through the device to fill essentially all of the traps. Measurements of this effect in diamond samples outside ATLAS and the predicted fluences in the presence of LHC collisions predict that the diamonds should become fully pumped within tens of minutes when the ATLAS instantaneous luminosity is 10^33^ cm^−2^ s^−1^.

**Fig. 13:**
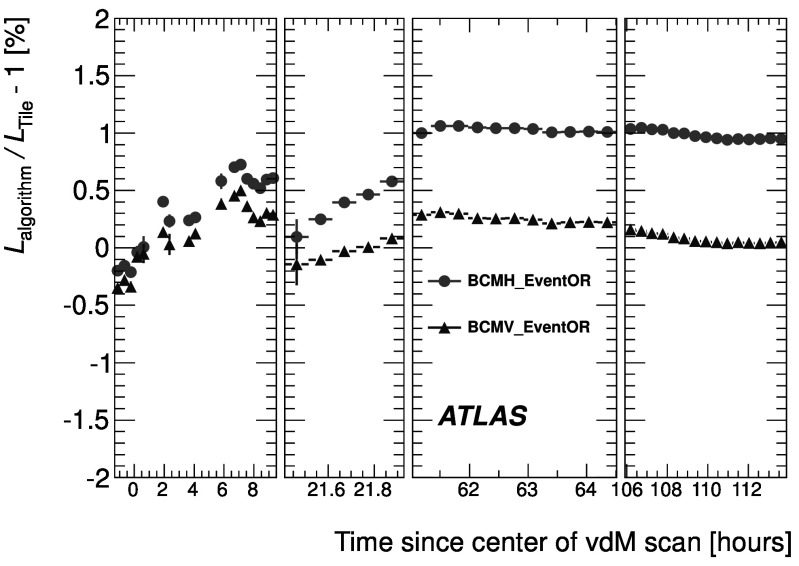
Fractional deviation of BCMH_EventOR and BCMV_EventOR luminosity values with respect to TileCal as a function of time since the May 2011 *vdM* scan. The TileCal luminosity scale is calibrated to LUCID_EventOR at the time of the *vdM* scan. The *vdM* scan was performed immediately following an LHC technical stop, when there had been no collisions for about 2 weeks

In the 2011 BCM data it has been observed that the apparent luminosity scale of the different sides of the BCM detectors tends to vary by up to about 1 % immediately after an extended period with no beam in the LHC. Figure 13 shows the fractional deviation of the BCMH_EventOR and BCMV_EventOR luminosity values from the luminosity measured by TileCal. The *vdM* calibration occurs near the start of the period shown in this figure, and a clear drift of the BCMH_EventOR luminosity scale is observed during the first fill and the start of the second fill, until settling at an asymptotically stable value. The drift of the BCMH_EventOR luminosity from the calibrated value is estimated to be +1.0 %, while the BCMV_EventOR luminosity is consistent with no significant net drift by the end of this time interval. Comparable shifts are observed in the BCM_EventAND luminosity scales. Similar patterns are observed after each LHC technical stop, a two or three week period during physics running, scheduled approximately every two months to allow for machine development and equipment maintenance. Within a couple of fills after each technical stop has ended and normal physics collisions have resumed, the BCM luminosity scale is observed to return, with rather good reproducibility, to the level recorded before the technical stop.

One interpretation of these data is that a small amount of annealing at the few percent level can occur during the technical stops. In the first few low-luminosity fills after a technical stop, some amount of “micro-pumping” takes place to refill these short-lifetime traps. The first fill shown in Fig. 13 is the *vdM* scan, which takes place right after the May 2011 technical stop. With an average luminosity around 3×10^30^ cm^−2^ s^−1^, this fill does not provide enough particle fluence through the BCM detectors to fully pump the short-lifetime traps. By the time of the third fill, where the luminosity reaches 4×10^32^ cm^−2^ s^−1^, the particle fluences since the technical stop are sufficient to return the detectors to their asymptotic response.

To account for this short-term change in the BCMH detector response, the BCMH luminosity scale has been corrected by the observed 1.0 % drift after the *vdM* scan. No correction has been applied to the BCMV_EventOR algorithm which is used to set the physics luminosity scale, but an additional systematic uncertainty of ±0.25 % has been applied as an estimate of the uncertainty due to this effect.

### TileCal calibration

As described in Sect. 4.4, the TileCal PMT currents from selected cells are calibrated with respect to the luminosity observed by the LUCID_EventOR algorithm at relatively low *μ* values. This current-based luminosity measurement is not absolutely calibrated, and does not provide bunch-by-bunch information, but is still a valuable cross-check of the stability of the other luminosity algorithms.

In the 2010 data, the total TileCal PMT current for a common group of cells is calibrated during a single LHC fill taken in October 2010. The calibration is performed by fitting the TileCal response as a function of the LUCID_EventOR luminosity over a range 50–100×10^30^ cm^−2^ s^−1^ with a first-order polynomial, where the constant term accounts for any pedestal or non-collision backgrounds present in the TileCal currents. This cross-calibrated luminosity value is then compared to LUCID_EventOR for all of the 2010 *pp* data where the luminosity was greater than 35×10^30^ cm^−2^ s^−1^. This luminosity represents the approximate threshold above which the luminosity-based current signal is large enough to be resolved. The RMS residual deviation between TileCal and LUCID is found to be about 0.2 % when comparing the average luminosity measured over a time range of about 2 minutes.

For the calibration method used in the 2011 data, a few cells around |*η*|=1.25 with the highest observed currents are compared to the LUCID_EventOR luminosity at the peak of the *vdM* scan. The TileCal pedestals are explicitly measured using data taken at the start of the fill before the beams are put into collision, and the pedestal-corrected TileCal currents are assumed to be directly proportional to the luminosity (with no constant offset). The LUCID luminosity at the peak of the *vdM* scan, which is itself calibrated by the scan at that point in time, is simply used to set the proportionality constant for each TileCal cell. These few calibrated cells are then compared to other TileCal cells in a fill shortly after the *vdM* scan when the luminosity is in the range 100–200×10^30^ cm^−2^ s^−1^ which is high enough to produce a reasonable current in all cells. The proportionality constants for these remaining cells are determined by comparing the pedestal-corrected currents to the luminosity measured by the subset of cells which were directly calibrated during the *vdM* scan. This two-stage calibration is necessary because the total luminosity during the *vdM* scan is too low to provide reasonable currents to all of the TileCal cells used to measure luminosity. The result is a TileCal calibration which is nearly independent of LUCID or any other detector in 2011.

The calibration of individual cells in 2011 allows all available cells to be used at any given time to provide a luminosity, which is important in 2011 due to an increasing number of tripped TileCal cells over the course of the year. Since the set of available cells can vary significantly over time, this method is more sensitive to the residual variations of the cell calibration constants. For the 2011 data, the RMS variation of the TileCal luminosity measurement is estimated to be about 0.5 % based on the agreement between individual cells and the typical number of calibrated cells available to make a measurement.

Additionally, the response of the TileCal PMTs showed variations in time related to the exposure of the detector to collisions. A downward drift of the mean PMT response was observed during data-taking periods, and an upward drift back to an asymptotically stable value was observed after a few days during a technical stop when there were no collisions. The typical size of this variation is around 1 %. This effect has been identified during calibration runs with a caesium-137 source that circulates among the TileCal cells and during laser calibration runs, where a laser signal is directly injected into the PMTs. Comparison of the luminosity measured by specific TileCal cells also confirms a time variation based on the rates of exposure seen by each individual cell. The TileCal laser calibration system is used to derive a global correction factor as a function of time based on the observed change in mean PMT response. This global correction improves the time stability of the TileCal luminosity, but as discussed further in Sect. 8.1 it does not remove the effect completely. Performing cell-by-cell corrections is unfeasible as the statistical error on the individual cell corrections would be too large.

### FCal calibration

Similarly, the FCal high-voltage (HV) currents are calibrated to one of the other detectors at one time to provide a luminosity measurement which can be used to check the stability of other methods. The FCal needs a higher instantaneous luminosity than TileCal (a minimum value around 1×10^32^ cm^−2^ s^−1^) to have a significant current signal. In order to check the validity of the calibration throughout the 2010 data-taking period, the calibrated FCal luminosity is compared to the LUCID_EventOR luminosity for a set of runs recorded during October 2010 when the luminosity was high enough for the FCal technique to work. The RMS residual variation between FCal and LUCID is found to be about 0.5 %. For 2011, a similar calibration was performed between FCal and BCMV_EventOR during a single run. The FCal HV lines are selected for luminosity determination based on their noise, and lines that are connected to shorted calorimeter electrodes are excluded. Individual HV currents are then compared to BCMV_EventOR during an LHC fill in September when the beams were purposely separated to provide a wide range of *μ* values in a short period of time. These so-called “*μ* scans” are also used to assess the *μ* dependence of various algorithms as described in Sect. 8.2. The *μ*-scan data provide the largest range of luminosities to calibrate the FCal current data accurately, and a linear fit is applied to extract calibration parameters for each FCal HV line. These calibrations are then applied to all measured HV currents in 2011 to provide a measured luminosity per HV line, and these individual measurements are averaged to produce a single FCal luminosity measurement.

## Luminosity stability

To produce the integrated luminosity values used in ATLAS physics analyses, a single algorithm is chosen to provide the central value for a certain range of time, with the remaining calibrated algorithms providing independent measurements to evaluate systematic uncertainties on the stability of these results. The LUCID_EventOR algorithm is primarily used in 2010 where the large visible cross-section makes it more sensitive to the relatively low luminosity delivered in that year. In 2011 the BCMV_EventOR algorithm is primarily used, due to the better relative stability of this detector compared to either BCMH or LUCID during the 2011 run.

The calibration of *σ*_vis_ is performed on only a few occasions (only once in 2011) and at a relatively low value of *μ* compared to the range of *μ* values routinely seen in physics operations, particularly in 2011 where peak values of *μ*≃20 for certain BCIDs were not uncommon. As discussed in Sect. 6.1.12, the number of interactions per bunch crossing (*μ*) is equivalent to the luminosity per bunch crossing and provides an intuitive unit to describe pile-up conditions.

Two additional sources of uncertainty are evaluated, which are related to the stability of the calibrated results when applied to the entire 2010 and 2011 data samples. The first is the long-term stability of each algorithm with respect to time, and the second is the linearity of the calibrated luminosity value with respect to the interaction rate *μ*. In each case, the agreement between all available detectors and algorithms is used to limit the possible systematic variation of the primary algorithm used to deliver physics luminosity results.

### Long-term stability

One key source of potential uncertainty is the assumption that the $\overline{\sigma}_{\mathrm{vis}}$ calibration determined in a set of *vdM* scans is stable across the entire 2010 or 2011 data set. Several effects could degrade the long-term stability of a given detector, including slow drifts in the detector response and sensitivity to varying LHC beam conditions, particularly the total number of colliding bunches. Because the number of colliding bunches increased rather monotonically during both the 2010 and 2011 data-taking periods, it is not possible to disentangle these two effects, so the tests of long-term stability should be viewed as covering both possibilities.

**Fig. 14:**
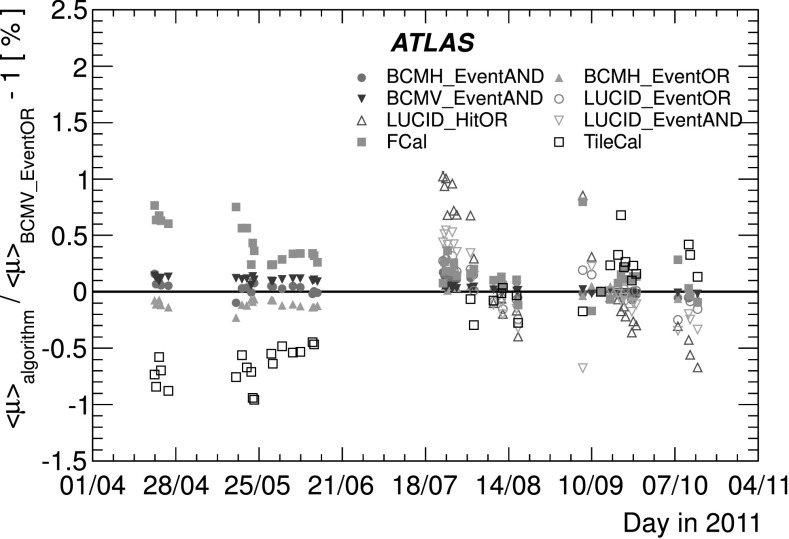
Fractional deviation of the mean interaction rate obtained using different algorithms from the BCMV_EventOR value as a function of time in 2011. Each point shows the mean deviation of the rate in a single run from the rate in a reference run taken in the middle of September. Statistical uncertainties per point are negligible

Figure 14 shows the interaction rate ratio of a given algorithm to the reference algorithm as a function of time in 2011. Each point shows the average number of interactions per bunch crossing measured by a particular algorithm divided by the number measured by BCMV_EventOR, averaged over one ATLAS run. The average number of interactions per bunch crossing, 〈*μ*〉, is the number of interactions per bunch *μ* averaged over all BCIDs with colliding bunch pairs, and must be used for any comparison with TileCal or FCal. The figure shows the relative variation of this ratio over time compared to a single fill in September which is used to provide a reference point, and comes approximately four months after the *vdM* scan in May. The variation seen on the left-hand side of this plot indicates the level of long-term stability from the *vdM* scan until this time in mid-September.

The various BCM algorithms are very stable with respect to each other, with agreement at the level of a few tenths of a percent over the entire 2011 run (the first few fills with low numbers of colliding bunches after each technical stop are not shown in this figure). This demonstrates the reproducibility of the BCM luminosity scale after each technical stop as discussed in Sect. 7.4. The LUCID data are shown only for the period of operation without gas from July onwards. Some variation at the level of ±0.5 % can be seen for the LUCID_Event algorithms, with somewhat larger variations observed for LUCID_HitOR. These variations are observed to be correlated with drifts in the PMT gains inferred from measurements of single-photon pulse-height distributions in the LUCID data.

The FCal luminosity scale is observed to change by about −0.5 % with respect to BCMV_EventOR from early to late 2011. Studies have shown that this variation is actually the result of a residual non-linearity in the FCal luminosity response. Since the average luminosity increased considerably from early to late 2011 due to the increase in the number of colliding bunches, this non-linearity with total luminosity manifests itself as an apparent drift on the time stability plot. The TileCal luminosity is observed to undergo a slow drift with respect to BCMV_EventOR at the level of 1 % over the course of 2011. In contrast to the FCal, this variation has been shown not to be dependent on luminosity, but rather is likely due to residual PMT gain variations which are not corrected by the TileCal laser calibration system.

Based on the observed variation with time between the various algorithms shown in Fig. 14, a systematic uncertainty on long-term stability, which includes any effects related to dependence on the number of colliding bunches or other operational conditions seen in the 2011 data, is set at ±0.7 %. Similar tests on the 2010 data show consistency at the level of ±0.5 %, where very good agreement is observed between the LUCID, BCM, TileCal, and FCal luminosity measurements.

### Interaction rate dependence

A final key cross-check is the level of agreement between the calibrated luminosity algorithms as a function of *μ*, the number of interactions per bunch crossing. In 2010, the measured values of *μ* in normal physics operations were in the range 0<*μ*<5, and a direct comparison of the four LUCID and BCMH algorithms over this range showed agreement at the ±0.5 % level. In 2011, the measured values of *μ* seen in physics data are considerably larger, with most data in the range 4<*μ*<20. The effects of pile-up increase at larger interaction rates, and it is important to verify that the various algorithms still provide an accurate and linear measurement of the luminosity up to the highest values of *μ* observed in the data.

**Fig. 15:**
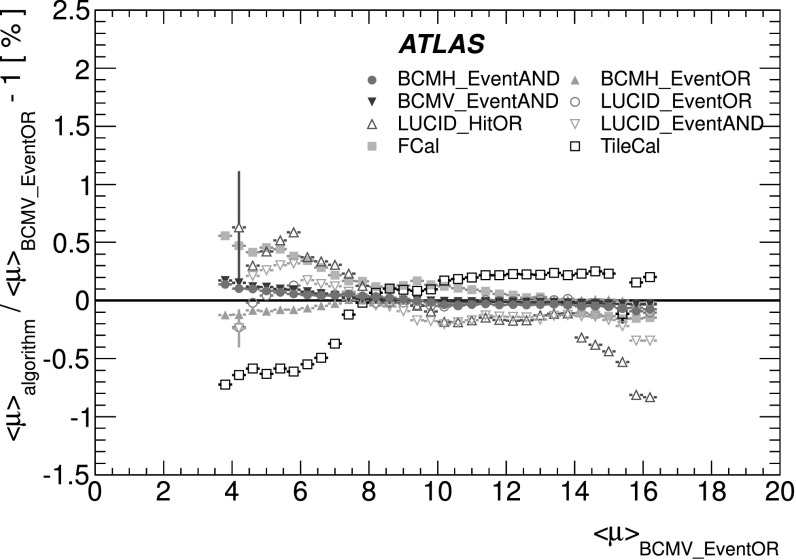
Fractional deviation of the average number of interactions per bunch crossing 〈*μ*〉 (averaged over BCIDs) obtained using different algorithms from the BCMV_EventOR value as a function of 〈*μ*〉. Statistical uncertainties are shown per point, but generally are negligible

A first way to assess the linearity is to take the data presented in Fig. 14 and calculate the interaction rate ratio as a function of the average number of interactions per bunch crossing 〈*μ*〉. This is shown in Fig. 15. Because the calorimeter methods measure only the interaction rate averaged over all colliding bunches 〈*μ*〉, the range of this comparison is smaller than the BCID-sensitive methods which test the full *μ* range. Since there is no absolute linearity reference available, the agreement between multiple algorithms with different acceptances and analysis methods is used to demonstrate consistency with each other, under the assumption that it is highly unlikely that they would all deviate from linearity in exactly the same way.

Again, since there is a ramp-up in the number of interactions per bunch crossing with time in 2011, issues with time stability are reflected in this figure as an apparent 〈*μ*〉 dependence. The large variation in TileCal is a good example, as the data with 〈*μ*〉<8 were recorded largely before the July technical stop, while the data with 〈*μ*〉>8 came mostly after this technical stop. The FCal variation appears to be a genuine non-linearity, although studies show that this is most accurately described as a dependence on total luminosity (not 〈*μ*〉). The LUCID_HitOR response varies by up to ±0.5 %, although this is also most likely explained by the variations seen in the time stability. The remaining algorithms all agree at the level of ±0.5 %, although this distribution does not test the linearity of the algorithms all the way down to the *vdM* calibration at *μ*≈2.

To improve the characterization of the *μ* dependence in the range 2<*μ*<10, without complications from long-term stability, a series of “*μ*-scans” was performed in 2011 to provide a direct measurement of the linearity of the various luminosity algorithms. The *μ*-scans are performed at the end of normal physics operations by separating the beams by ±5 *σ*_b_ in 19 steps, using the same procedure as employed in the *vdM* scans. Because this was done at the end of an LHC fill when the luminosity is fairly modest, and the entire scan can be performed in less than an hour, the cost of this procedure in terms of lost physics luminosity is much less than performing a *vdM* scan.

During these *μ*-scans, special triggers are used to collect large samples of events for the vertex-based luminosity algorithm from two specific BCIDs. In addition to the online algorithms, the TileCal and FCal current measurements also provide useful data during these scans.

**Fig. 16:**
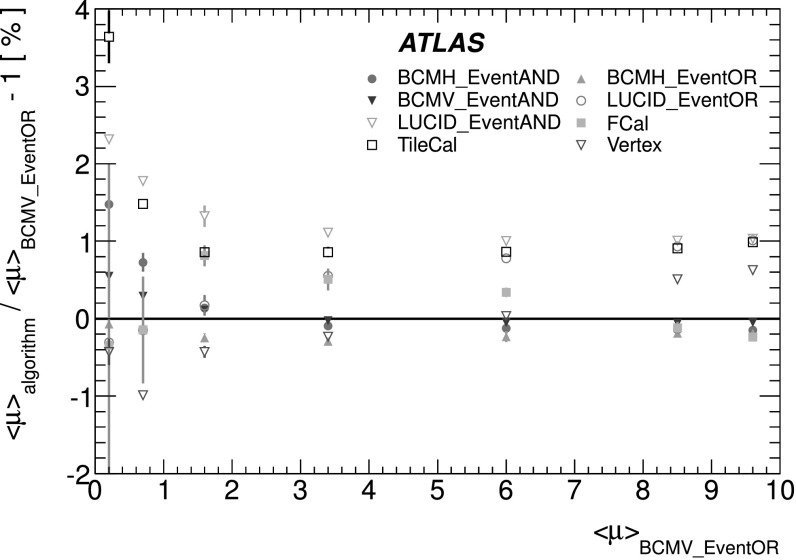
Fractional deviation of the average number of interactions per bunch crossing 〈*μ*〉 (averaged over BCIDs) obtained using different algorithms from the BCMV_EventOR value as a function of 〈*μ*〉. Data shown are taken during a *μ*-scan, where the beams are purposely separated to sample a large *μ* range under similar conditions. Statistical uncertainties are shown per point, but generally are negligible for 〈*μ*〉>2

Figure 16 shows the *μ*-scan data comparison for several algorithms. Because single-beam backgrounds become relatively more important as the beams are separated, the LUCID and BCM data were corrected for both afterglow and single-beam backgrounds using a procedure similar to that employed in the *vdM* scans.

The approximately constant offsets between algorithms are the result of drifts in the calibrated scales due to long-term stability. The linearity consistency is assessed by looking for a slope in the luminosity ratio with respect to the reference algorithm BCMV_EventOR. All of the algorithms show good linearity from the 〈*μ*〉 value where the *vdM* scan is performed (around 〈*μ*〉=2) up to the 〈*μ*〉 value observed in nominal physics operations (here around 〈*μ*〉=10). A deviation of around 1 % is observed in the FCal luminosity over this range, which is consistent with the dependence on total luminosity also observed in Fig. 15. The TileCal data agree very well with BCM, which is significant since the TileCal luminosity scale is cross-calibrated to LUCID_EventOR during the *vdM* scan taken four months earlier. The LUCID_EventOR data also agree with BCM at the ±0.5 % level, while LUCID_EventAND deviates by a few percent at the lowest luminosity values. This is interpreted as an imperfect subtraction of the single-beam background which is complicated by the presence of afterglow in this physics-based LHC filling pattern. Deviations of LUCID_EventAND are not observed at low luminosity in the *vdM* scan, shown in Fig. 10, where the background correction can be performed more accurately. The vertex counting data are also shown in Fig. 16 for the two BCIDs which were recorded with a special trigger during this time. The vertex luminosity increases by about 1 % over the range of this figure, which is consistent with the additional systematic uncertainties on the vertex counting technique. These uncertainties, related to the vertex masking and fake vertex corrections, grow with the interaction rate and are estimated to reach ±2 % by an interaction rate of *μ*=10.

**Fig. 17:**
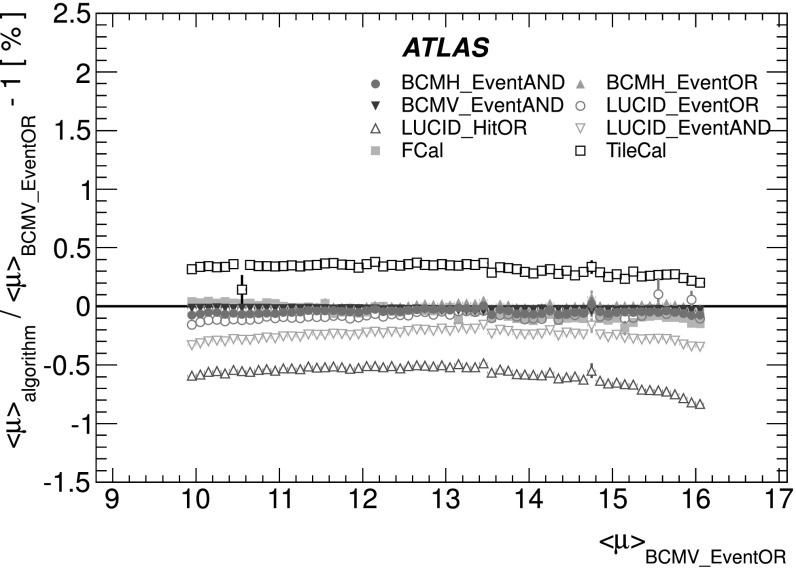
Fractional deviation of the average number of interactions per bunch crossing 〈*μ*〉 (averaged over BCIDs) obtained using different algorithms from the BCMV_EventOR value as a function of 〈*μ*〉. Data from only a single LHC fill are shown. Statistical uncertainties are shown per point, but generally are negligible

A final test of *μ* dependence is performed by comparing the luminosity ratio between algorithms as a function of 〈*μ*〉 for a single LHC fill. This comparison, shown in Fig. 17 for a fill in October 2011, provides a way to assess the linearity independently from any long-term stability effects up to the very highest *μ* values observed in 2011. Here the shapes of the curves are directly sensitive to variations in the linearity as a function of 〈*μ*〉, while the overall shifts of each algorithm up or down result from variations in the long-term stability. So while TileCal and LUCID_HitOR luminosity scales are both seen to deviate from BCMV_EventOR by up to 0.5 %, this variation is expected from the data shown in Fig. 14. Each algorithm shows a linear response with respect to BCMV_EventOR, with the largest variations observed for LUCID_HitOR at the 0.5 % level.

As a result of all the information available, a systematic uncertainty of ±0.5 % has been applied to account for any possible *μ* dependence in the extrapolation from the low-*μ* *vdM* scan calibration to the higher-*μ* physics data in 2011. More limited data were available in 2010, although the extrapolation range was significantly smaller (*μ*≤5). Similar comparisons for the 2010 data lead to an uncertainty due to a possible *μ* dependence of ±0.5 %.

### Total systematic uncertainty

Table 9 lists the contributions to the total systematic uncertainty on the luminosity scale provided for physics analyses in the 2010 and 2011 data samples. The bunch population product and other calibration uncertainties are related to the *vdM* scan calibration described in Sects. 5 and 6. The afterglow and BCM stability uncertainties are related to particular conditions in 2011 as described in Sect. 7. The long-term stability and *μ* dependence uncertainties are both related to extrapolating the *vdM* calibration to the entire 2010 and 2011 data samples as described in Sect. 8. The single largest improvement between 2010 and 2011 has come from a better understanding of the bunch population product during the *vdM* scan.

**Table 9:** Relative uncertainty on the calibrated luminosity scale broken down by source. The *vdM* scan calibration uncertainty has been separated into the uncertainty on the bunch population product and the uncertainties from all other sources

Uncertainty Source	$\delta\mathcal{L}/\mathcal{L}$	
	2010	2011
Bunch Population Product	3.1 %	0.5 %
Other *vdM*		
Calibration Uncertainties	1.5 %	1.4 %
Afterglow Correction		0.2 %
BCM Stability		0.2 %
Long-Term Stability	0.5 %	0.7 %
*μ* Dependence	0.5 %	0.5 %
Total	3.5 %	1.8 %

## Conclusions

The luminosity scales determined by the ATLAS Collaboration for 2010 and 2011 have been calibrated based on data from dedicated beam-separation scans, also known as van der Meer (*vdM*) scans. Systematic uncertainties on the absolute luminosity calibration have been evaluated. For the 2010 calibrations, the uncertainty is dominated by the understanding of the bunch charge product, while for 2011 the uncertainty is mostly due to the accuracy of the *vdM* calibration procedure. Additional uncertainties are evaluated to assess the stability of the calibrated luminosity scale over time and over variation in operating conditions, most notably the number of interactions per bunch crossing. The combination of these systematic uncertainties results in a final uncertainty on the ATLAS luminosity scale during *pp* collisions at $\sqrt{s} = 7~\mathrm{TeV}$ of $\delta\mathcal{L}/ \mathcal{L} = \pm3.5~\%$ for the 47 pb^−1^ of data delivered to ATLAS in 2010 and $\delta\mathcal{L}/ \mathcal{L} = \pm 1.8~\%$ for the 5.5 fb^−1^ delivered in 2011. These results include explicit corrections for beam–beam effects in the *vdM* calibration scans that were not understood until late in the luminosity analysis and were therefore not applied to the luminosity scale used in any ATLAS publication prior to July of 2013. Consequently, the luminosity scale used in previous ATLAS results should be scaled down by 1.9 % in 2010 and 1.4 % in 2011.

## Footnotes

1 ATLAS uses a right-handed coordinate system with its origin at the nominal interaction point (IP) in the centre of the detector, and the *z*-axis along the beam line. The *x*-axis points from the IP to the centre of the LHC ring, and the *y*-axis points upwards. Cylindrical coordinates (*r*,*ϕ*) are used in the transverse plane, *ϕ* being the azimuthal angle around the beam line. The pseudorapidity is defined in terms of the polar angle *θ* as *η*=−lntan(*θ*/2).

2 The CTP inhibits triggers (causing deadtime) for a variety of reasons, but especially for several bunch crossings after a triggered event to allow time for the detector readout to conclude. Any new triggers which occur during this time are ignored.

3 A closed orbit bump is a local distortion of the beam orbit that is implemented using pairs of steering dipoles located on either side of the affected region. In this particular case, these bumps are tuned to offset the trajectory of either beam parallel to itself at the IP, in either the horizontal or the vertical direction.

4 The *β* function describes the single-particle motion and determines the variation of the beam envelope along the beam orbit. It is calculated from the focusing properties of the magnetic lattice (see for example Ref. [14]).

5 The tune of a storage ring is defined as the betatron phase advance per turn, or equivalently as the number of betatron oscillations over one full ring circumference.

6 For 2010, the correction is computed for scans IV and V only, because the bunch intensities during the earlier scans are so low as to make beam–beam effects negligible.

7 An exception is the BCM_EventAND algorithms, for which the visible cross-section is computed using the convolved beam sizes measured by the corresponding, higher-rate BCM_EventOR algorithm, thereby providing slightly improved statistical accuracy.

8 The number of interactions per bunch crossing (*μ*) is determined from the luminosity per bunch crossing as $\mu= \mathcal{L}\, \sigma_{\mathrm{inel}}/f_{\mathrm{r}}$ where the inelastic cross-section is assumed to be *σ*_inel_=71.5 mb. This value of *σ*_inel_ comes from a phenomenological model implemented in PYTHIA6.4 [22] which was found to be consistent with the early luminosity calibrations in 2010 [2]. This cross-section is only used to present the luminosity data in terms of a more intuitive quantity, and does not enter into the luminosity determination in any way.
